# Assertive Community Treatment and ACT-Derived Outreach Models: A Scoping Review of Adaptations, Fidelity and Outcomes

**DOI:** 10.1007/s10597-026-01607-8

**Published:** 2026-03-26

**Authors:** Soraya Vega-Martínez, Manuel Pabón-Carrasco, Germán Bola-Saiz, Xabier Marichalar-Mendia, Saloa Unanue-Arza

**Affiliations:** 1 Department of Public Health, University of the Basque Country, Leioa, Spain; 2 Bizkaia Mental Health Network, Assertive Community Treatment (ACT) Team, Buenavista Health Centre, Osakidetza, Portugalete, Biscay, Spain; 3 Research Group PAIDICTS1050, “Complex Care, Chronicity and Health Outcomes”, Faculty of Nursing, Physiotherapy and Podiatry, University of Seville, Seville, Spain; 4 Bizkaia Mental Health Network, Zamudio Hospital, Osakidetza, Biscay, Spain; 5 Department of Nursing I, University of the Basque Country, Leioa, Spain; 6 Research Group: GIU21/042, University of the Basque Country, Leioa, Spain; 7 Clinical nursing and community health, BioCruces Health research Institute, Barakaldo, Spain

**Keywords:** Mental disorders, Mentally Ill persons, Community mental health services, Scoping review

## Abstract

Severe Mental Illness (SMI) represents a significant public health challenge associated with long-term disability, premature mortality, and high social and health care costs. Assertive Community Treatment (ACT) is an intensive community-based model for people with SMI who have difficulty engaging with conventional services and are at high risk of repeated hospitalizations. Over time, this model has been adapted to meet diverse needs, contexts, and populations. To map and synthesize evidence on ACT and ACT-derived outreach models, including adaptations, fidelity-related characteristics, comparator conditions, and outcomes. Following Joanna Briggs Institute methodology and PRISMA-ScR guidelines, we searched Medline (PubMed), Web of Science, Cochrane Library, Scopus, CINAHL, PsycInfo, ProQuest, IBECS, and LILACS without year restrictions. Two reviewers independently assessed eligibility, with disagreements resolved by a third reviewer. The protocol was registered in the Open Science Framework (DOI: 10.17605/OSF.IO/DPNYT). We included 129 studies (1990–2023), comprising randomized controlled trials, descriptive studies, and quasi-experimental designs. Evidence came predominantly from high-income countries and included diverse populations. Alongside classic ACT/PACT, adaptations included Flexible ACT, early psychosis community intervention, forensic and homelessness-focused models, and integrated dual-diagnosis approaches. Implementation features, fidelity reporting, comparator services, and outcome measurement varied, particularly for recovery-oriented outcomes. ACT and ACT-derived outreach services represent a continuum of intensive community mental health care. Reduced inpatient service use and improved engagement were commonly reported, whereas quality of life, social inclusion, vocational outcomes, and recovery were more variable. Better reporting of intervention characteristics, fidelity indicators, and comparator conditions is needed.

**Supplementary Information:**

The online version contains supplementary material available at 10.1007/s10597-026-01607-8.

## Introduction

Severe mental illness (SMI) encompasses serious, persistent, and often long-term psychiatric conditions with a profound impact on individual functioning, including schizoaffective disorders, major depressive disorder with psychotic features, certain highly disabling personality disorders, and schizophrenia, which affects approximately one in every 200 adults (National Institute of Mental Health [NIMH], 1987; World Health Organization [WHO], 2019, 2022a). Although SMI is frequently operationalized using diagnosis-based criteria, contemporary frameworks also emphasize the role of functional impairment, disability, and service needs, which may vary across settings and health care systems (Pina et al., 2024).

SMI has been identified as one of the leading causes of disability worldwide (WHO, 2022b, 2022c). Individuals living with SMI have a life expectancy that is 10 to 20 years shorter than that of the general population, primarily due to untreated physical comorbidities and barriers in accessing health care services (WHO, 2022b). From an economic perspective, its burden is substantial, driven by indirect costs related to productivity loss, health care expenditures, and social security (WHO, 2022c; OECD & European Union, 2018). Importantly, recovery-oriented approaches recognize that people living with SMI can pursue and achieve meaningful recovery, autonomy, and social participation, and recommend avoiding language that implies hopelessness or permanence (Mental Health Coordinating Council, 2022; Psychiatric Rehabilitation Association [PRA], 2003). This scenario highlights the urgent need for effective interventions that not only reduce healthcare system costs but also promote psychosocial recovery and community integration among patients (WHO, 2022c).

Assertive Community Treatment (ACT), developed as the Program of Assertive Community Treatment (PACT), is an intensive, team-based community mental health model intended to support people with SMI in living in the community as an alternative to long-term inpatient treatment. (Marx et al., 1973; Stein & Test, 1980). Over time, ACT evolved from primarily facilitating community living following deinstitutionalization to sustaining community tenure by preventing avoidable hospitalizations, ensuring continuity of support, and advancing recovery-oriented goals (Stein & Test, 1980). This model is characterized by intensive, community-based services delivered by multidisciplinary teams with shared caseload responsibility, low staff-to-patient ratios, assertive outreach and in vivo service delivery, frequent high-intensity contacts, continuity of care, and 24/7 crisis availability, providing proactive, flexible, and individualized treatment, support, and rehabilitation tailored to each patient’s needs (Marx et al., 1973; Stein & Test, 1980).

ACT originated in the 1970 s when Mary Ann Test, Arnold Marx, and Leonard Stein developed the model at the Mendota Mental Health Institute in Madison, Wisconsin, United States (Marx et al., 1973; Stein & Test, 1980). Its implementation emerged as a response to the “revolving door” phenomenon, a recurrent issue among patients with schizophrenia and other severe psychotic disorders who experienced repeated hospitalizations without achieving sustained stability in the community (Phillips et al., 2001; Stein & Test, 1980). Early evaluations showed that reorganizing hospital-based teams to deliver coordinated, in vivo support in clients’ natural settings, rather than relying on inpatient-centered models, was associated with shorter hospital stays, fewer relapses, and improved autonomy in daily living (Marx et al., 1973). Building on these findings, ACT was increasingly recognized not only as a clinical intervention but also as a transformative service innovation that addressed fragmented post-deinstitutionalization systems by establishing a fixed point of responsibility for comprehensive and continuous community support (Rochefort, 2019). As the model expanded beyond its original setting, ACT evolved into a flexible set of outreach-based community services, raising enduring tensions between maintaining fidelity to core ingredients and adapting structures to local resources, workforce capacity, and policy environments (Rochefort, 2019). This evolution marked a turning point in mental health service delivery, laying the groundwork for integrated, coordinated, and person-centered community approaches that offered a structured alternative to repeated hospitalization and supported sustained community tenure (Phillips et al., 2001; Williamson, 2002). Consistent with this international diffusion, ACT has been adopted and adapted across multiple health care systems in North America, Europe, and Oceania, including countries such as the United States, Canada, Australia, the United Kingdom, Spain, Norway, Sweden, and the Netherlands, among others. Across these contexts, the model has been operationalized through a range of ACT-derived outreach service configurations, including Flexible ACT in the Netherlands, Mobile Intensive Rehabilitation Teams (MIRT) in Australia, and Assertive Outreach teams in the United Kingdom. These adaptations have typically emerged in response to population needs and service system constraints, shaped by factors such as resource availability, workforce training, and the level of integration between mental health and social care services (Mueser et al., 1998). In this review, we use the term ACT-derived adaptations to refer to outreach-based multidisciplinary community teams that preserve core ACT ingredients (e.g., low caseload/high-intensity contact, shared team responsibility, in vivo service delivery, and continuity of support), while allowing structured modifications in intensity, population focus, or service configuration according to local contexts.

Several comprehensive systematic reviews have examined ACT and intensive case management interventions for individuals with SMI, primarily focusing on effectiveness outcomes such as psychiatric hospitalization, service utilization, and clinical indicators. Among these, the most influential and up-to-date synthesis is the Cochrane systematic review by Dieterich et al. (2017), which evaluated intensive case management for SMI and reported heterogeneous findings depending on context, comparator services, and model fidelity.

However, the purpose of the present review is not to estimate intervention effects. Instead, we aimed to map the breadth and evolution of ACT and ACT-derived outreach service models across countries and health care systems, describing how these models have been implemented, adapted, and operationalized over more than three decades. Methodological guidance indicates that scoping reviews are particularly appropriate when the objective is to clarify key concepts and definitions, examine how research has been conducted, identify key characteristics related to a concept, and provide an overview of the volume and nature of the evidence base, especially when there is substantial heterogeneity in interventions and study designs (Munn et al., 2018).

Given the widespread implementation of ACT as a community-based approach for individuals with SMI, the time elapsed since its inception, and the diversity of contexts in which it has been applied, a scoping review is timely to map the extent, nature, and evolution of the evidence on ACT and ACT-derived outreach models, and to characterize how these services have been implemented and adapted across settings over time.

## Materials and Methods

### Study Design

A scoping review was conducted with the aim of providing a comprehensive overview of the available evidence, identifying key concepts, research trends, and the current state of knowledge regarding ACT (Pollock et al., 2021). The process followed the methodological framework of the Joanna Briggs Institute (Peters et al., 2020), ensuring a structured and systematic identification, selection, and analysis of the literature.

The presentation of findings adhered to the PRISMA extension for Scoping Reviews (Preferred Reporting Items for Systematic Reviews and Meta-Analyses), thereby guaranteeing transparency in data extraction and reporting (Tricco et al., 2018). Article eligibility was independently assessed by two reviewers (S.V-M and S.U-A) to ensure methodological rigor and consensus-based decision-making. Discrepancies were resolved through discussion and, when necessary, by consulting a third reviewer (M.P-C), an expert in mental health and research methodology. The review protocol was registered in the Open Science Framework (DOI: 10.17605/OSF.IO/DPNYT).

### Research Question and Eligibility Criteria

The guiding research question for this scoping review was: What evidence exists regarding the use, implementation, and reported outcomes of ACT in individuals diagnosed with SMI? The Population–Concept–Context (PCC) framework was employed, as recommended by the Joanna Briggs Institute, to formulate exploratory questions that facilitate the identification and synthesis of available evidence while ensuring clarity in inclusion and exclusion criteria (Peters et al., 2020).

### Population

This review included studies involving individuals diagnosed with severe mental illness (SMI), also referred to as severe and persistent mental illness (SPMI). As originally conceptualized by the U.S. National Institute of Mental Health (NIMH), SMI refers to a heterogeneous group of severe psychiatric disorders of prolonged duration, associated with varying levels of disability and social dysfunction, and requiring coordinated support across multiple health and social sectors and community resources (NIMH, 1987). In practice, SMI has traditionally been operationalized using three core dimensions: (1) a qualifying diagnostic category, (2) a long-term course (persistence over time), and (3) functional impairment (NIMH, 1987). Although conceptual debates remain, this framework continues to be widely cited in the literature (Zumstein & Riese, 2020). Importantly, contemporary recovery-oriented and rights-based frameworks emphasize that a long-term course should not be interpreted as inevitable chronicity or a hopeless prognosis, and that many people living with SMI can achieve meaningful recovery, autonomy, and social participation when supported through person-centered, community-based systems of care (World Health Organization, 2021; Mental Health Coordinating Council, 2022).

The diagnostic criterion encompasses conditions characterized by distorted perception of reality, behavior incongruent with the social context, severe affective disturbances, or profoundly impaired interpersonal functioning. These include non-organic psychotic disorders, major affective disorders, and highly disabling personality disorders (Donald & Stajduhar, 2019; NIMH, 1987). According to the 11th Revision of the International Classification of Diseases (ICD-11) (WHO, 2019), the following diagnostic categories are included: 6A20 Schizophrenia; 6A21 Schizoaffective disorders; 6A22 Schizotypal disorder; 6A24 Delusional disorder; 6A2Y Other specified schizophrenia or primary psychotic disorders; 6A2Z Schizophrenia or other primary psychotic disorders, unspecified; 6A60 Bipolar type I disorder; 6A61 Bipolar type II disorder; 6A70.4 Major depressive episode, single, severe with psychotic symptoms; 6A71 Recurrent depressive disorder; 6B20 Obsessive-compulsive disorder; and 6D10 Personality disorder.

Given that ICD-11 came into force in early 2022, we mapped these diagnostic categories to the previous ICD-10 classification (WHO, 1992), as many studies may still use ICD-10 terminology (Table 1).

**Table 1:** Comparison of ICD-11 and ICD-10 diagnostic categories

ICD-11	ICD-10
6A20 Schizophrenia	F20 Schizophrenia
6A21 Schizoaffective disorder	F21 Schizotypal disorder
6A22 Schizotypal disorder	F25 Schizoaffective disorder
6A24 Delusional disorder	F22 Delusional disorder
6A2Y Other specified schizophrenia or other primary psychotic disorders	F28 Other non–substance-induced psychotic disorders not due to known physiological conditions
6A2Z Schizophrenia or other primary psychotic disorders, unspecified	F29 Unspecified non–substance-induced psychosis not due to known physiological conditions
6A60 Bipolar type I disorder	F31 Bipolar disorder
6A61 Bipolar type II disorder	F31 Bipolar disorder
6A70.4 Major depressive disorder, single episode, severe with psychotic symptoms	F32.3 Major depressive disorder, single episode, severe with psychotic symptoms
6A71 Recurrent depressive disorder	F33 Major depressive disorder, recurrent
6B20 Obsessive-compulsive disorder	F42 Obsessive-compulsive disorder
6D10 Personality disorder	F60 Specific personality disorders

Source: World health organization, 2019; World health organization, 1992. Table developed by the authors

Regarding the duration of illness, this dimension includes disorders with a clinical course of two years or longer, characterized by progressive functional deterioration over the preceding six months despite remission of symptoms (NIMH, 1987). Duration criteria also encompass individuals who have required, on more than one occasion, psychiatric care of greater intensity than standard outpatient services (e.g., community mental health centers), as well as those who have received long-term continuous residential support (excluding hospitalizations) that may have disrupted their everyday life circumstances (NIMH, 1987).However, contemporary community mental health systems increasingly adopt prevention- and early intervention-oriented approaches, extending intensive outreach and coordinated supports to individuals in earlier phases of illness or at high risk of relapse and functional decline, with the aim of preventing long-term disability and reducing the likelihood of a prolonged trajectory of service dependence (World Health Organization, 2021). In addition, some ACT-derived or integrated community models explicitly address complex comorbidity profiles, including substance use disorders, through integrated approaches to co-occurring conditions (Penzenstadler et al., 2019). In certain settings, assertive outreach approaches have also been extended to populations with neurodevelopmental conditions (e.g., learning disability) where these contribute to high support needs and functional impairment (Prakash et al., 2007).

The final defining feature concerns the disabling potential of severe and recurrent disorders that cause significant functional impairment in major life activities. Continuous or intermittent presence of at least two of the following criteria is required (NIMH, 1987): unemployment, sheltered employment, or markedly limited work skills and/or a poor employment history; reliance on public financial assistance and need for help to obtain it; difficulty establishing or maintaining a support network; need for assistance with basic or instrumental activities of daily living (e.g., hygiene, nutrition, or money management); and socially inappropriate behavior requiring intervention from mental health or judicial systems.

For this review, studies that focused exclusively on individuals with substance use disorders or intellectual disabilities were excluded. Only studies in which most participants met predefined criteria or operational indicators of SMI (e.g., diagnosis combined with long-term functional impairment and high service needs, or eligibility for intensive community-based services) were included.

### Concept

Studies were included if they examined services described by the authors as ACT or ACT-derived outreach models for individuals with SMI in community settings. Articles primarily evaluating fidelity or adherence to ACT implementation models were excluded. Specifically, we excluded studies that exclusively focused on the development or validation of fidelity instruments or on implementation/process evaluations that did not report program characteristics and outcomes relevant to mapping ACT delivery. However, we did not exclude studies that reported key ACT elements (e.g., caseload ratio, multidisciplinary team composition, contact intensity, 24/7 coverage) or that used fidelity assessments as part of describing the intervention.

### Context

Studies conducted in community service settings were included, encompassing outpatient services, mobile mental health units, and intervention teams operating within patients’ daily environments and across various levels of the health care system. The key inclusion criterion was a focus on providing community-based services and supports outside traditional hospital settings. Studies based exclusively in hospital, institutional, or correctional environments without direct linkage to community mental health services were excluded.

### Identification of Relevant Studies

This review included primary studies employing quantitative, qualitative, or mixed-methods designs, covering both experimental and non-experimental research. Secondary sources were also identified. Only studies available in full text were eligible for inclusion and analysis. Research protocols and conference abstracts were excluded. The review was restricted to publications available in English and Spanish.

### Information Sources and Search Strategy

From the outset, Medical Subject Headings (MeSH) were used to design the search strategy, complemented by free-text terms in both English and Spanish, based on the previously described Population–Concept–Context (PCC) framework. The databases searched included Medline (PubMed), Web of Science (WoS), Cochrane Library, Scopus, CINAHL, PsycInfo, ProQuest, IBECS, and LILACS.

The search strategies were adapted to the syntax and indexing of each database and are detailed in Supplementary Table S1. No publication date restrictions were applied. The search was conducted between 26 January 2024 and 11 May 2024. Only studies published in English or Spanish, regardless of their country of origin, were included.

### Study selection

After completing the search in each database, all retrieved information was managed using the reference manager Zotero^®^ (Corporation for Digital Scholarship, 2023), a tool used to organize bibliographic references and documents. Duplicate records were removed, and the remaining references were then exported to a Microsoft Excel^®^ spreadsheet (Microsoft Corporation, 2018) to facilitate organization and analysis.

**Fig. 1:**
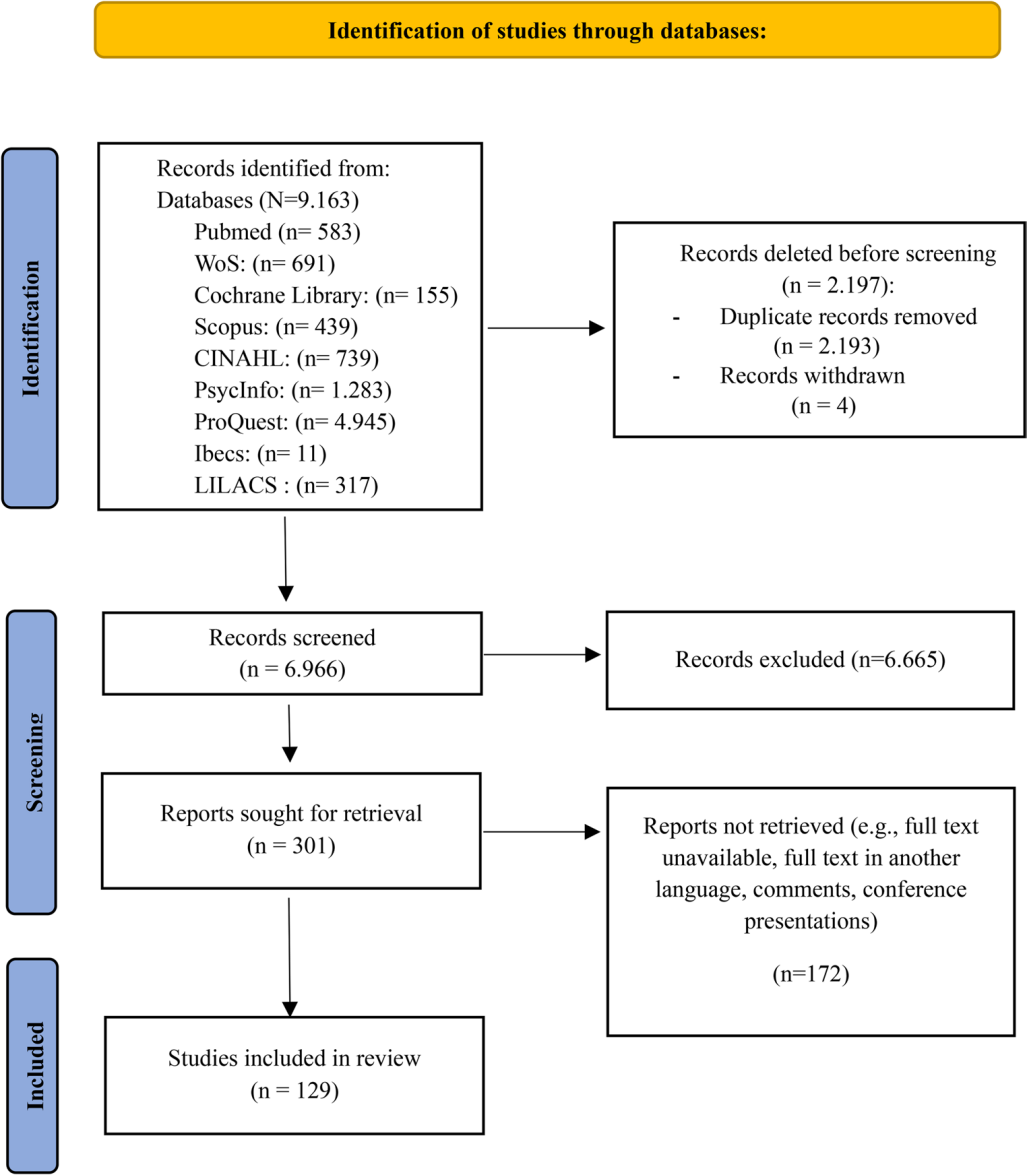
PRISMA flowchart for article selection. **Footnote:** A *record* refers to a bibliographic entry retrieved from a database search (e.g., title/abstract). A *report* refers to a full-text publication (e.g., journal article, thesis, or conference paper). A *study* refers to a single unique investigation; multiple reports may originate from the same study

The screening and selection process was independently performed by pairs of reviewers. Titles and abstracts were first screened to exclude studies that did not include relevant keywords or did not meet the predefined inclusion criteria. Full-text articles of potentially eligible studies were then assessed to confirm eligibility. Discrepancies were resolved through discussion and consensus, and any unresolved disagreements were reviewed by a third member of the research team. The complete selection process, along with the reasons for exclusion, is presented in Fig. 1, following the PRISMA-ScR model (Tricco et al., 2018).

When multiple reports referred to the same underlying study, they were linked and counted as a single study in the final inclusion.

### Data Extraction and Analysis

A structured data extraction form was developed following the methodological recommendations of the Joanna Briggs Institute for conducting scoping reviews (Tricco et al., 2018). The table included the following variables: study title/reference, authors, year of publication, country of origin, study design and ACT-model classification, participant characteristics (sample size, diagnosis, age, and sex), inclusion criteria, comparison groups (if applicable), residential/setting context, and data collection period. Outcomes/measures assessed and the main study findings were also extracted. In addition, implementation-related variables were collected to map ACT and ACT-derived models based on core ACT ingredients, including caseload ratio, multidisciplinary team composition, in vivo/community-based care, 24/7 availability, duration/continuity of support, fidelity tools/scores, and key model characteristics.

## Results

A total of 9,163 records were identified through electronic database searches (Fig. 1). After removing 2,197 records, including 2,193 duplicates and 4 withdrawn studies replaced by newer publications, 6,966 records remained for screening. Of the 301 full-text articles assessed for eligibility, 172 were excluded because the full text was unavailable, published in a language other than English or Spanish, or did not meet the inclusion criteria. Ultimately, 129 studies were included in the review. These studies were derived from a set of published articles, some of which reported more than one study.

The results are summarized in two main supplementary tables (Supplementary Tables S2 and S3). Table S2 reports the general characteristics of the included studies, including reference/year/country, study design and ACT model classification, participant characteristics (N, diagnosis, age, and sex), comparison groups (if applicable), inclusion criteria, residential/setting context, data collection period, outcomes/measures, and key findings. Table S3 maps the implementation characteristics of ACT and ACT-derived models based on core ACT ingredients, including caseload ratio, multidisciplinary team composition, in vivo/community-based care, 24/7 availability, duration/continuity of support, fidelity tools/scores (e.g., Dartmouth Assertive Community Treatment Scale (DACTS), Tool for Measurement of Assertive Community Treatment (TMACT), or other instruments), and key model characteristics.

The included studies were published between 1990 and 2023, with a marked increase in publications from 2010 onward, particularly between 2010 and 2016 (*n* = 69). In the past five years (2019–2023), 25 studies were published. Most studies were conducted in English-speaking and Northern European countries, primarily the United States (*n* = 54), the United Kingdom (*n* = 18), Denmark (*n* = 15), Canada (*n* = 15) and the Netherlands (*n* = 15).

A broad methodological diversity was observed. The majority were observational studies (*n* = 69), mainly retrospective cohort and prospective longitudinal designs. Quasi-experimental studies (*n* = 25) included those with matched or historical controls, as well as uncontrolled trials. A considerable number were randomized controlled trials (*n* = 39). Twelve studies conducted cost-effectiveness analyses (10 RCTs and 2 quasi-experimental studies). Additionally, systematic reviews (*n* = 5) (Abufarsakh et al., 2023; Dieterich et al., 2017; Smith et al., 2007; Vanderlip et al., 2017; Vijverberg et al., 2017) and meta-analyses (*n* = 7) (Burns, 2010; Burns et al., 2007; Coldwell et al., 2007; Goulet et al., 2022; Herdelin et al., 1999; Nordén et al., 2012; Randall et al., 2015) were included.

Data collection periods ranged from 1979 to 2021, encompassing both short-term (6–12 months) and extended follow-ups of up to 10 years (Killaspy et al., 2014) or 20 years (Hansen et al., 2023). Several studies featured multi-phase follow-ups at 12 months (Karow et al., 2020), 24 months (Clausen et al., 2020), or 36 months (Botha et al., 2014), while others employed retrospective comparisons of pre- and post-ACT implementation periods. Studies were conducted across diverse geographic and social contexts, including urban settings (*n* = 115), as well as rural, remote/frontier, and mixed environments. A total of 93 studies included comparison groups, most frequently contrasting ACT with standard community care, although not all studies incorporated control groups due to methodological constraints.

The inclusion criteria for participants generally required adults aged 18–65 years who met predefined criteria or operational indicators of SMI and who were enrolled in or eligible for ACT programs. Many studies also required one or more of the following: frequent psychiatric hospitalizations, poor treatment adherence, impaired social functioning, or risk of social exclusion. Broader inclusion criteria in some studies extended to homelessness or precarious housing, co-occurring substance use disorders, history of ineffective treatment in standard services, and need for intensive community-based services. Other studies applied more specific criteria, such as minimum treatment duration, defined functional impairment levels, residence in a specific geographic area, or exclusion of individuals with intellectual disabilities or serious medical conditions. Studies involving adolescent populations (*n* = 6) focused on severe psychiatric disorders, high-risk behaviors, and persistent refusal of outpatient treatment (Baier et al., 2013; Daubney et al., 2021; Mantzouranis et al., 2019). Notably, definitions and operationalization of SMI and eligibility for ACT/FACT-type services vary across health systems, and in some countries, SMI is conceptualized less as a diagnostic label and more as an indication for integrated, multidisciplinary community services (Westen et al., 2022, 2025).

Most studies (*n* = 123) included adult participants with schizophrenia, bipolar disorder, or other psychotic disorders, with sample sizes ranging from 10 to 6,030 participants and variable gender distributions. Six studies involved adolescent populations (Ahrens et al., 2007; Mantzouranis et al., 2019; Baier et al., 2013; Daubney et al., 2021; Vijverberg et al., 2017; Rohenkohl et al., 2022). Regarding subgroups, 36 studies included homeless individuals, 11 included participants with forensic or judicial backgrounds, 5 focused on people with active substance use, and 3 addressed first-episode psychosis.

A wide range of ACT model implementations was observed, dominated by the classic ACT model based on Stein and Test (*n* = 40), followed by established adaptations such as Flexible ACT (*n* = 7), OPUS (*n* = 6), and Forensic ACT (*n* = 6). Integrated models such as Housing First with ACT (*n* = 8) were also identified, along with locally adapted versions such as Assertive Outreach (*n* = 10), Hamburg Model of Integrated Care (ACCESS-ACT, *n* = 4), and other variants including ACT-Elderly, Dual-Diagnosis ACT, Rural ACT, MIRT, Training in Community Living, and Intensive Assertive Community Intervention Team (*n* = 8). These models differed substantially in team size, caseload, intensity of contact, outreach capacity, and recovery orientation, and should not be interpreted as equivalent to the classic ACT model. Rather, they represented a spectrum of services drawing selectively on ACT principles and adapted to local contexts, populations, and system constraints (Supplementary Table S3).

Common elements across models included intensive and flexible community-based intervention, multidisciplinary services, unlimited individualized follow-up, and 24/7 service availability. Many programs reported high contact frequency (up to two home or community visits per day), low staff-to-patient ratios (typically 1:6 to 1:15), daily (or at minimum several-times-per-week) team meetings, and interventions such as psychoeducation, social skills training, and family support. Recovery-oriented principles were increasingly embedded and explicitly reported across ACT and ACT-derived models, including integration of housing resources and vocational rehabilitation support.

Multidisciplinary teams, whose composition varied according to country, care model, and program version, were responsible for delivering ACT interventions. Typically, teams comprised psychiatrists, mental health nurses, social workers, clinical psychologists, occupational therapists, rehabilitation specialists, vocational counselors, and peer support workers with lived experience. Some models also included primary care physicians, psychiatric consultants, addiction specialists, family therapists, physiotherapists, case managers, and administrative or coordination staff. In certain cases, teams incorporated forensic mental health therapists, dietitians, or community support workers, reflecting a highly adaptable structure tailored to service user needs and available resources.

A wide variety of standardized instruments were used to measure outcomes, including the Global Assessment of Functioning (GAF) (*n* = 36), Brief Psychiatric Rating Scale (BPRS) (*n* = 35), and Positive and Negative Syndrome Scale (PANSS) (*n* = 27) for psychiatric symptoms; the Health of the Nation Outcome Scales (HoNOS) (*n* = 16) for clinical and social status; the Manchester Short Assessment of Quality of Life (MANSA) (*n* = 9); and the Clinical Global Impression (CGI) (*n* = 9). Additional tools included structured clinical interviews, self-report questionnaires, and administrative records on hospitalization number and duration, treatment adherence, suicide risk, social functioning, quality of life, satisfaction, community integration, and cost-effectiveness.

Outcomes were variably reported across studies and settings. Regarding hospital service utilization, several studies described reductions in admissions and inpatient days and fewer emergency department visits, whereas others reported limited or no differences compared with control conditions, particularly where usual care was intensive or where implementation fidelity and population profiles differed. Improvements were also reported for treatment adherence, quality of life, and psychosocial functioning (MANSA, GAF, HoNOS). Some studies additionally reported reductions in psychiatric symptom severity (BPRS, PANSS) and improved clinical/functional stability (CGI, HoNOS).

Patient and family satisfaction with treatment, assessed through self-report measures, was commonly reported as high. Several studies also reported benefits in community integration and housing stability, particularly among vulnerable groups such as people experiencing homelessness, youth, individuals with dual diagnoses, and those with forensic histories.

From an economic perspective, ACT was frequently reported as cost-effective, with reductions in hospitalization-related costs and more efficient use of resources in some settings, although several studies noted initial increases in costs due to the model’s intensive follow-up and low staff-to-patient ratios. Overall, findings were not uniform across outcomes, as some studies reported no significant changes in social or occupational functioning or community integration, suggesting that the impact of ACT and ACT-derived models may vary depending on context, population characteristics, comparator intensity, and implementation fidelity.

## Discussion

This scoping review analyzed and synthesized evidence from 129 studies published between 1990 and 2023, providing a broad overview of ACT implementation over more than three decades. One of the main findings was the marked methodological diversity across studies, ranging from long-term randomized controlled trials to descriptive and quasi-experimental designs. Such variability, combined with differences in how the model has been implemented across settings, limits the ability to draw consistent and fully generalizable conclusions applicable to all community mental health systems, an issue previously highlighted in earlier reviews (Abufarsakh et al., 2023; Dieterich et al., 2017; Smith & Newton, 2007; Vanderlip et al., 2017).

In terms of implementation contexts, most studies were conducted in high-income countries (primarily English-speaking and Northern European settings) and focused on metropolitan or regional catchment areas. This concentration may shape the available evidence base, potentially limiting transferability to settings with different resource levels, service structures, and sociocultural determinants of care. It therefore reinforces the need for research in more diverse cultural, geographic, and economic contexts (Dieterich et al., 2017; Patel et al., 2007).

Since its origins in Madison, Wisconsin (Stein & Test, 1980), ACT has maintained several core principles that are well established in the literature: multidisciplinary intervention, intensive and continuous community-based care, low staff-to-patient ratios, and round-the-clock availability (Allness & Knoedler, 1998; Bond & Salyers, 2004; Burns & Firn, 2002; Marx et al., 1973; Phillips et al., 2001). Although multiple adaptations have emerged, some integrating psychosocial and rehabilitative components or targeting specific populations such as the OPUS program for early psychosis in youth (Jørgensen et al., 2000), fidelity to the original model has been variable. This variability likely contributes to the heterogeneity of outcomes across settings and complicates direct comparisons between studies and service configurations (Burns et al., 2007; Dieterich et al., 2017; Munch Nielsen et al., 2023).

Across most programs, core professional roles consistently included mental health nurses, psychiatrists, and social workers, reflecting the model’s multidisciplinary, team-based roots (Marx et al., 1973) and its later evolution toward a more holistic, recovery- and person-centered approach to care (Dieterich et al., 2017). Many teams were complemented by occupational therapists, clinical psychologists, peer workers, and vocational specialists, whose involvement often depended on available resources and the specific needs of the populations served (WHO, 2022c). These included youth in early psychosis programs (Randall et al., 2015), individuals experiencing homelessness (Coldwell & Bender, 2007), individuals with co-occurring substance use disorders (Abufarsakh et al., 2023), forensic populations (Goulet et al., 2022; Tomar et al. 2020), and older adults (Stobbe et al., 2014).

A key contribution of this review is the conceptual framing of ACT as a continuum rather than a single homogeneous intervention. We distinguish between classic/high-fidelity ACT teams aligned with the original PACT model and structured ACT-derived adaptations tailored to local service ecosystems and specific populations (e.g., OPUS early intervention, Housing First with ACT support, forensic ACT/FACT approaches, youth-ACT, and modified ACT models in resource-limited contexts). This distinction is important because many contemporary “ACT” implementations no longer correspond strictly to classic ACT ingredients (e.g., 24/7 coverage, shared caseload, or very low staff-to-patient ratios), and outcomes should therefore be interpreted in relation to the service configuration actually delivered rather than the model label alone (Burns et al., 2007; Dieterich et al., 2017).

Regarding key outcomes, many studies reported improvements in treatment adherence and reductions in the duration of psychiatric hospitalization, outcomes aligned with the model’s foundational rationale and therefore anticipated in well, implemented teams (Arahanthabailu et al., 2022; Coetzee et al., 2022; Daubney et al., 2021; Doré-Gauthier et al., 2020; Schöttle et al., 2019; Thoegersen et al., 2019; Vidal et al., 2020). These patterns are broadly consistent with prior reviews describing ACT’s primary impact on hospital service utilization and community tenure (Bond & Drake, 2015; Burns et al., 2007; Dieterich et al., 2017), in line with principles outlined in early ACT research (Marx et al., 1973; Stein & Test, 1980). However, outcomes related to quality of life, social integration, and vocational reintegration remained heterogeneous and less consistently reported across studies (Clausen et al., 2020; Dieterich et al., 2017; Rohenkohl et al., 2022). Importantly, these domains are also more likely to be shaped by structural determinants (e.g., housing, welfare systems, labour-market access) that extend beyond clinical team inputs, which may partly explain inconsistent findings across settings.

The apparent effectiveness of ACT appears increasingly context-dependent, particularly as comparator conditions have evolved over time. Several included studies compared ACT not only against treatment as usual (TAU), but also against established community mental health teams (CMHT), intensive case management (ICM), assertive outreach services, or FACT models. These active comparators may attenuate detectable between-group differences, especially in less fragmented healthcare systems where baseline service intensity is higher. Outcomes should therefore be interpreted cautiously and considered in light of both comparator intensity and model fidelity (Bond & Drake, 2015; Burns et al., 2007; Dieterich et al., 2017; Firn et al., 2013; Vanderlip et al., 2017).

The heterogeneity observed in quality-of-life, social inclusion, and vocational outcomes may partly reflect the structural complexity and disadvantage of the populations targeted by ACT and ACT-derived outreach services. Across the included literature, eligibility criteria commonly prioritized individuals with high service needs, impaired functioning, and repeated hospital use, frequently combined with social exclusion (e.g., housing instability, limited support networks, justice involvement, or co-occurring substance use). For example, 36 studies explicitly focused on individuals experiencing homelessness, illustrating the extension of ACT principles toward housing stability and community integration in highly marginalized groups (Coldwell & Bender, 2007; Stergiopoulos et al., 2015). Eleven studies examined forensic or justice-involved populations, where outcomes are shaped not only by clinical needs but also by legal supervision, stigma, and continuity-of-care barriers (Lamberti et al. 2017a, b; Morrissey et al. 2007).

With respect to integrated dual-diagnosis components, 5 studies included participants with co-occurring substance use problems, although terminology and operationalization varied considerably across countries and time periods. This variability reflects broader international differences in how SMI is defined and the extent to which addiction is addressed as an integral component of severe mental health needs. Adaptations such as integrated dual-diagnosis ACT may therefore be understood as pragmatic evolutions of the original model in response to complex, real-world service demands (Abufarsakh et al., 2023; Tranberg et al., 2024).

Specialized adaptations such as Forensic ACT explicitly integrate mental health treatment with criminal justice supervision. Randomized and quasi-experimental studies have reported associations with fewer arrests, reduced time spent in jail, and lower rates of psychiatric hospitalization, although these findings should be interpreted in light of study design limitations, selection effects, and contextual factors (Cusack et al. 2010; Lamberti et al. 2017a, b; Marquant et al. 2018b).

Similarly, Veterans Affairs ACT-like programs (e.g., Mental Health Intensive Case Management) retain core ACT ingredients while incorporating enhanced coordination with physical health and social benefit systems. Reported outcomes include reductions in hospitalization and improved engagement, although these effects may be influenced by the integrated nature of the broader healthcare system in which they are embedded (Mohamed et al., 2010; Slade et al., 2013).

Additionally, three studies focused on individuals experiencing a first episode of psychosis, a key group in both research and clinical practice given the importance of early intervention for longer-term trajectories. Including this population highlights ACT’s adaptability, but outcomes from early-intervention variants should not be treated as equivalent to classic ACT due to differences in duration, staffing models, and recovery targets (Bertelsen et al. 2008; Hastrup et al. 2013; Nordentoft et al. 2015a, b).

In this regard, the OPUS programme represents a particularly relevant adaptation, combining modified ACT principles with structured psychosocial components (e.g., family intervention and social skills training) and a time-limited early-intervention philosophy. While OPUS is not equivalent to classic ACT, its inclusion illustrates how ACT-derived intensive outreach has been reconfigured to support engagement, relapse prevention, and recovery trajectories in younger populations. These findings reinforce the need to separate “classic ACT” effects from outcomes observed in early-intervention outreach variants that operate with different duration, staffing models, and recovery targets (Jørgensen et al. 2000; Bertelsen et al. 2008; Nordentoft et al. 2015a, b).

Beyond OPUS, youth-ACT and adolescent-focused mobile intensive teams represent another emerging direction of ACT-derived care, reflecting the broader shift toward stage-specific interventions and functional recovery goals earlier in the illness course. Although the evidence remains limited, existing syntheses suggest potential benefits in symptom severity, functioning, and reduced inpatient use in youth populations, consistent with ACT principles but requiring dedicated evaluation frameworks and more robust comparative designs (Vijverberg et al., 2017).

Flexible Assertive Community Treatment (FACT), originally introduced in the Netherlands as a hybrid adaptation of classic ACT/PACT, enables step-up and step-down intensity within the same multidisciplinary team according to service users’ needs (van Veldhuizen, 2007). Over the past decade, FACT has expanded across several countries and has been implemented within diverse community mental health systems, with growing attention to its cross-national development, implementation, and model clarification (Tang et al., 2025). Emerging evidence indicates that outcomes may be closely linked to implementation quality and fidelity monitoring. In Denmark, a quasi-experimental comparison found no superiority of FACT over ACT in personal and social functioning; patients treated by FACT teams demonstrated lower functioning at follow-up, although no differences were observed in client satisfaction or working alliance (Munch Nielsen et al., 2023). The authors cautioned that these findings should be interpreted carefully due to baseline differences, attrition, and the absence of fidelity assessment. Conversely, observational studies in Norway and Austria reported reductions in total and involuntary inpatient days, readmissions, and related costs following FACT implementation (Brekke et al., 2025; Tholen et al., 2024). Collectively, these findings suggest that variability in outcomes may reflect differences in fidelity, local adaptation, and system integration rather than inherent superiority or inferiority of FACT as a model. Recent discussions increasingly frame FACT as an evolving implementation framework whose effectiveness may depend on structured fidelity monitoring and contextual optimisation (Westen et al., 2025). Accordingly, future research should prioritize fidelity-informed and component-level evaluations, moving beyond binary model comparisons (ACT vs. FACT) to examine how implementation processes, adaptation, and quality monitoring influence outcomes within recovery-oriented systems.

A major methodological issue identified in this review was substantial variability in outcome measures, limiting cross-study comparability. Differences in instruments used to assess similar domains, inconsistent follow-up time points, and variable reporting of comparator intensity complicate synthesis and interpretation. These findings reinforce previous calls for greater standardization and clearer international consensus on outcome frameworks in community mental health research (Bond & Drake, 2015; Dieterich et al., 2017).

Finally, fidelity assessment represents a critical interpretive dimension. Inconsistent fidelity reporting and structural deviations across teams labelled as “ACT” complicate interpretation of outcome variability and limit attribution of observed outcomes to specific ACT components. Mapping implementation characteristics alongside reported outcomes provides added context for interpretation but cannot fully resolve residual confounding arising from population differences, system-level factors, and study design limitations.

### Strengths and Limitations

Among the main strengths of this review is the inclusion of evidence spanning several decades of ACT implementation, enabling a comprehensive examination of a well-established yet evolving model. The use of a scoping review methodology was particularly appropriate for mapping and synthesizing accumulated findings across time and diverse healthcare and social contexts, especially given the heterogeneity in study designs and outcome measures.

An additional strength is that this review does not conceptualize ACT as a single uniform intervention but instead maps classic/high-fidelity ACT alongside ACT-derived adaptations and service variants (e.g., OPUS/early psychosis outreach, homelessness-focused programs, forensic adaptations, and integrated dual-diagnosis components). This approach reflects contemporary service realities and supports a more nuanced interpretation of outcomes based on the characteristics of services actually delivered rather than intervention labels alone. By linking implementation features with reported outcomes, this review adds contextual depth to the interpretation of heterogeneous findings.

However, several limitations should be acknowledged. ACT has evolved substantially over time, from early medically oriented approaches toward more recovery-oriented, person-centered, and rights-based community care, and evaluation priorities have shifted accordingly. We did not conduct a decade-stratified synthesis of outcomes, which might have provided additional insight into temporal changes in implementation focus and the emergence of recovery-oriented indicators. Future reviews could address this by analyzing models and outcomes by decade and explicitly distinguishing early ACT evidence from contemporary adaptations.

The interpretability of comparative findings is also limited by substantial heterogeneity in comparator conditions. Several studies compared ACT not only with treatment as usual (TAU), but also with active and well-established services (e.g., CMHT, ICM, assertive outreach teams, or FACT/Flexible ACT models). Such variability likely attenuates detectable between-group differences in more developed systems and complicates cross-study interpretation, reinforcing the need to consider outcomes in relation to comparator intensity and service ecosystems.

Not all potentially relevant studies identified during screening could be retrieved in full text despite searching multiple databases and repositories. Additionally, the search was restricted to publications in English and Spanish, which may have resulted in the exclusion of relevant studies published in other languages. This language restriction may contribute to geographic and cultural bias within the evidence base.

Furthermore, heterogeneity in study designs, outcome definitions, follow-up durations, and measurement tools constrained the identification of consistent patterns across domains. As a scoping review, this study did not include formal risk-of-bias assessment or quantitative synthesis, limiting the ability to estimate effect sizes or establish comparative effectiveness. Fidelity reporting was inconsistent, and in many studies the structural characteristics of teams labelled as “ACT” were incompletely described, restricting precise evaluation of implementation quality. Finally, most available evidence originated from high-income countries, particularly English-speaking and Northern European settings, limiting the representativeness and generalizability of findings to low-resource or culturally diverse contexts.

### Future Research Directions

Based on the findings of this review, several priorities for future research can be identified.

First, multicenter and international studies examining ACT and ACT-derived outreach services in diverse economic, healthcare, and sociocultural contexts are needed to better assess transferability, feasibility, and context-specific adaptation requirements. Expanding research beyond high-income, predominantly Western settings would enhance the representativeness of the evidence base and clarify how structural and system-level factors influence implementation and outcomes.

Second, longitudinal research employing standardized, comparable, and transparently reported outcome measures should be encouraged to facilitate clearer interpretation of service user trajectories across clinical, functional, social, and recovery-oriented domains. The development and adoption of core outcome sets for community mental health services could strengthen cross-study comparability and reduce heterogeneity in reporting practices.

Third, future studies should provide detailed and transparent descriptions of both intervention models and comparator conditions. This includes clearly specifying whether programs correspond to classic/high-fidelity ACT or structured adaptations (e.g., FACT/Flexible ACT, OPUS/early intervention models, forensic ACT variants, Housing First with ACT support), and systematically reporting key structural components such as staffing ratios, shared caseload arrangements, outreach intensity, 24/7 availability, integration with housing and addiction services, and the use of fidelity tools (e.g., DACTS, TMACT, or equivalent measures). Such reporting would support component-level analyses and more precise interpretation of outcome variability.

Fourth, recovery should be explicitly conceptualized and operationalized as a central outcome domain in contemporary evaluations. Future research should distinguish between clinical recovery (e.g., symptom reduction and stability) and personal or functional recovery (e.g., autonomy, empowerment, hope, meaningful social roles, participation, and citizenship). Greater conceptual clarity and measurement consistency in recovery-oriented outcomes would improve international comparability and align evaluation frameworks with rights-based and person-centered community mental health strategies.

Finally, further research is warranted on adapted ACT versions targeting specific populations, including children and adolescents, individuals with limited social support networks, justice-involved populations, people experiencing homelessness, and individuals with co-occurring substance use disorders. Carefully designed comparative and implementation studies in these subgroups may contribute to the refinement of context-responsive and inclusive models of intensive community-based care, while clarifying which components are most relevant for specific service-user profiles.

## Conclusion

ACT is widely described in the literature as a reference framework for intensive community-based mental health care. This scoping review suggests that, over time, ACT has evolved from classic high-fidelity configurations toward a broader continuum of ACT-derived outreach services and structured adaptations developed to respond to diverse populations and service contexts.

Considerable variation was observed in model implementation, comparator conditions, team composition, and adherence to core ACT ingredients. These findings suggest that outcomes should be interpreted in relation to the specific service configuration delivered rather than inferred solely from the “ACT” designation.

The range of models described in this review includes flexible and early-intervention configurations, as well as specialized ACT adaptations targeting specific and structurally disadvantaged populations, including forensic services and veteran-focused ACT-like programs. Within these configurations, observed outcomes appear to be influenced not only by clinical processes but also by the degree of integration with legal, housing, and broader healthcare systems.

From a contemporary perspective, recovery-based and rights-based frameworks increasingly shape community mental health priorities. Future evaluations would benefit from explicitly incorporating recovery as a core outcome domain and from examining how fidelity to key ACT principles (multidisciplinary in vivo care, continuity, proactive outreach and intensity) can be balanced with necessary contextual adaptation within evolving service systems.

## Supplementary Information

Below is the link to the electronic supplementary material.

ESM 1 Supplementary Material (DOCX 179 KB)

## Data Availability

No datasets were generated or analysed during the current study.

## References

[CR1] Aagaard, J., & Kølbæk, P. (2016). Predictors of clinical outcome of Assertive Community Treatment (ACT) in a rural area in Denmark: An observational study with a two-year follow-up. *Community Mental Health Journal,**52*(8), 908–913. 10.1007/s10597-015-9908-y 26143244 10.1007/s10597-015-9908-y

[CR2] Aagaard, J., & Müller-Nielsen, K. (2011). Clinical outcome of assertive community treatment (ACT) in a rural area in Denmark: A case-control study with a 2-year follow-up. *Nordic Journal of Psychiatry,**65*(5), 299–305. 10.3109/08039488.2010.544405 21174491 10.3109/08039488.2010.544405

[CR3] Aagaard, J., Tuszewski, B., & Kølbæk, P. (2017). Does assertive community treatment reduce the use of compulsory admissions? *Archives of Psychiatric Nursing,**31*(6), 641–646. 10.1016/j.apnu.2017.07.008 29179833 10.1016/j.apnu.2017.07.008

[CR4] Abufarsakh, B., Kappi, A., Pemberton, K. M., Williams, L. B., & Okoli, C. T. C. (2023). Substance use outcomes among individuals with severe mental illnesses receiving assertive community treatment: A systematic review. *International Journal of Mental Health Nursing,**32*(3), 704–726. 10.1111/inm.13103 36534491 10.1111/inm.13103

[CR5] Ahrens, C., Frey, J., Knoedler, W. H., & Senn-Burke, S. C. (2007). Effect of PACT on inpatient psychiatric treatment for adolescents with severe mental illness: A preliminary analysis. *Psychiatric Services (Washington, D.C.),**58*(11), 1486–1488. 10.1176/ps.2007.58.11.1486 17978262 10.1176/ps.2007.58.11.1486

[CR6] Albert, N., Melau, M., Jensen, H., Emborg, C., Jepsen, J. R., Fagerlund, B., Gluud, C., Mors, O., Hjorthøj, C., & Nordentoft, M. (2017). Five years of specialised early intervention versus two years of specialised early intervention followed by three years of standard treatment for patients with a first episode psychosis: Randomised, superiority, parallel group trial in Denmark (OPUS II). *BMJ (Clinical Research Ed.),**356*, Article i6681. 10.1136/bmj.i6681 28082379 10.1136/bmj.i6681 PMC5228538

[CR7] Allness, D. J., & Knoedler, W. H. (1998). *The PACT model of community-based treatment for persons with severe and persistent mental illnesses: A manual for PACT start-up*. NAMI Campaign to End Discrimination, NAMI Anti Stigma Foundation.

[CR8] Arahanthabailu, P., Purohith, A. N., Kanakode, R., Praharaj, S. K., Bhandary, R. P., & Venkata Narasimha Sharma, P. S. (2022). Modified assertive community treatment program for patients with schizophrenia: Effectiveness and perspectives of service consumers from a South Indian setting. *Asian Journal of Psychiatry,**73*, Article 103102. 10.1016/j.ajp.2022.103102 35452965 10.1016/j.ajp.2022.103102

[CR11] Aubry, T., Bourque, J., Goering, P., Crouse, S., Veldhuizen, S., LeBlanc, S., Cherner, R., Bourque, P. É., Pakzad, S., & Bradshaw, C. (2019). A randomized controlled trial of the effectiveness of Housing First in a small Canadian City. *BMC Public Health,**19*(1), Article 1154. 10.1186/s12889-019-7492-8 31438912 10.1186/s12889-019-7492-8 PMC6704672

[CR10] Aubry, T., Goering, P., Veldhuizen, S., Adair, C. E., Bourque, J., Distasio, J., Latimer, E., Stergiopoulos, V., Somers, J., Streiner, D. L., & Tsemberis, S. (2016). A multiple-city RCT of housing first with Assertive Community Treatment for homeless Canadians with serious mental illness. *Psychiatric Services,**67*(3), 275–281. 10.1176/appi.ps.201400587 26620289 10.1176/appi.ps.201400587

[CR9] Aubry, T., Tsemberis, S., Adair, C. E., Veldhuizen, S., Streiner, D., Latimer, E., Sareen, J., Patterson, M., McGarvey, K., Kopp, B., Hume, C., & Goering, P. (2015). One-year outcomes of a randomized controlled trial of housing first with ACT in five Canadian cities. *Psychiatric Services,**66*(5), 463–469. 10.1176/appi.ps.201400167 25639993 10.1176/appi.ps.201400167

[CR12] Bahji, A., Stephenson, C., & Mazhar, M. N. (2024). Navigating the complex intersection of substance use and psychiatric disorders: The case for integrated care. *Journal of Clinical Medicine,**13*(4), Article 999. 10.3390/jcm13040999 38398311 10.3390/jcm13040999 PMC10889170

[CR13] Baier, V., Favrod, J., Ferrari, P., Koch, N., & Holzer, L. (2013). Early tailored assertive community case management for hard-to-engage adolescents suffering from psychiatric disorders: An exploratory pilot study. *Early Intervention in Psychiatry,**7*(1), 94–99. 10.1111/j.1751-7893.2012.00380.x 22765257 10.1111/j.1751-7893.2012.00380.x

[CR14] Bertelsen, M., Jeppesen, P., Petersen, L., Thorup, A., Øhlenschlaeger, J., le Quach, P., Christensen, T. Ø., Krarup, G., Jørgensen, P., & Nordentoft, M. (2008). Five-year follow-up of a randomized multicenter trial of intensive early intervention vs standard treatment for patients with a first episode of psychotic illness: The OPUS trial. *Archives of general psychiatry*, *65*(7), 762–771. ttps://doi.org/10.1001/archpsyc.65.7.762 18606949 10.1001/archpsyc.65.7.762

[CR15] Bodén, R., Sundström, J., Lindström, E., Wieselgren, I. M., & Lindström, L. (2010). Five-year outcome of first-episode psychosis before and after the implementation of a modified assertive community treatment programme. *Social Psychiatry and Psychiatric Epidemiology,**45*(6), 665–674. 10.1007/s00127-009-0108-3 19652896 10.1007/s00127-009-0108-3

[CR16] Bond, G. R., & Drake, R. E. (2015). The critical ingredients of assertive community treatment. *World Psychiatry,**14*(2), 240–242. 10.1002/wps.20234 26043344 10.1002/wps.20234 PMC4471983

[CR19] Bond, G. R., McDonel, E. C., Miller, L. D., & Pensec, M. (1991). Assertive community treatment and reference groups: An evaluation of their effectiveness for young adults with serious mental illness and substance abuse problems. *Psychosocial Rehabilitation Journal,**15*(2), 31–43. 10.1037/h0095785

[CR17] Bond, G. R., & Salyers, M. P. (2004). Prediction of outcome from the Dartmouth assertive community treatment fidelity scale. *CNS Spectrums,**9*(12), 937–942. 10.1017/s1092852900009792 15616478 10.1017/s1092852900009792

[CR18] Bond, G. R., Witheridge, T. F., Dincin, J., Wasmer, D., Webb, J., & De Graaf-Kaser, R. (1990). Assertive community treatment for frequent users of psychiatric hospitals in a large city: A controlled study. *American Journal of Community Psychology,**18*(6), 865–891. 10.1007/BF00938068 2091459 10.1007/BF00938068

[CR20] Bonsack, C., Adam, L., Haefliger, T., Besson, J., & Conus, P. (2005). Difficult-to-engage patients: A specific target for time-limited assertive outreach in a Swiss setting. *Canadian Journal of Psychiatry. Revue Canadienne De Psychiatrie,**50*(13), 845–850. 10.1177/070674370505001307 16483119 10.1177/070674370505001307

[CR22] Botha, U. A., Koen, L., Galal, U., Jordaan, E., & Niehaus, D. J. (2014). The rise of assertive community interventions in South Africa: A randomized control trial assessing the impact of a modified assertive intervention on readmission rates; a three year follow-up. *BMC Psychiatry,**14*, Article 56. 10.1186/1471-244X-14-56 24571621 10.1186/1471-244X-14-56 PMC3974055

[CR21] Botha, U. A., Koen, L., Joska, J. A., Hering, L. M., & Oosthuizen, P. P. (2010). Assessing the efficacy of a modified assertive community-based treatment programme in a developing country. *BMC Psychiatry,**10*, Article 73. 10.1186/1471-244X-10-73 20843301 10.1186/1471-244X-10-73 PMC2945974

[CR23] Brekke, E., Lamu, A. N., Angeles, R. C., Clausen, H., & Landheim, A. S. (2025). Changes in inpatient mental health treatment and related costs before and after flexible assertive community treatment: A naturalistic observational cohort study. *BMC Psychiatry,**25*, Article 164. 10.1186/s12888-025-06614-9 40001041 10.1186/s12888-025-06614-9 PMC11852869

[CR24] Burns, T. (2010). The rise and fall of assertive community treatment? *International Review of Psychiatry,**22*(2), 130–137. 10.3109/09540261003661841 20504053 10.3109/09540261003661841

[CR26] Burns, T., Catty, J., Dash, M., Roberts, C., Lockwood, A., & Marshall, M. (2007). Use of intensive case management to reduce time in hospital in people with severe mental illness: Systematic review and meta-regression. *BMJ,**335*(7615), Article 336. 10.1136/bmj.39251.599259.55 17631513 10.1136/bmj.39251.599259.55 PMC1949434

[CR25] Burns, T., & Firn, M. (2002). *Assertive outreach in mental health: A manual for practitioners*. Oxford University Press.

[CR27] Calsyn, R. J., Morse, G. A., Klinkenberg, W. D., Trusty, M. L., & Allen, G. (1998). The impact of assertive community treatment on the social relationships of people who are homeless and mentally ill. *Community Mental Health Journal*(6). 10.1023/a:1018711001348 10.1023/a:1018711001348 9833199

[CR28] Carton, A. D., Young, M. S., & Kelly, K. M. (2010). Changes in sources and perceived quality of social supports among formerly homeless persons receiving assertive community treatment services. *Community Mental Health Journal,**46*(2), 156–163. 10.1007/s10597-009-9185-8 19263220 10.1007/s10597-009-9185-8

[CR29] Chandler, D., & Spicer, G. (2002). Capitated assertive community treatment program savings: System implications. *Administration and Policy in Mental Health,**30*(1), 3–19. 10.1023/A:1021254615844 12546253 10.1023/a:1021254615844

[CR30] Chandler, D., Spicer, G., Wagner, M., & Hargreaves, W. (1999). Cost-effectiveness of a capitated assertive community treatment program. *Psychiatric Rehabilitation Journal,**22*(4), 327–336. 10.1037/h0095219

[CR31] Chetty, A., van der Westhuizen, D., Botha, U., & Koen, L. (2023). Integrated vs non-integrated treatment outcomes in dual diagnosis: A comparative review. *BMC Psychiatry,**23*(1), Article 276. 10.1186/s12888-023-04632-237081388

[CR32] Chow, W., Shiida, M., Shiida, T., Hirosue, A., Law, S., Leszcz, M., & Sadavoy, J. (2011). Adapting ACT to serve culturally diverse communities: A comparison of a Japanese and a Canadian ACT team. *Psychiatric Services (Washington, D.C.),**62*(8), 971–974. 10.1176/ps.62.8.pss6208_0971 21807841 10.1176/ps.62.8.pss6208_0971

[CR34] Clarke, G. N., Herinckx, H. A., Kinney, R. F., Paulson, R. I., Cutler, D. L., Lewis, K., & Oxman, E. (2000). Psychiatric hospitalizations, arrests, emergency room visits, and homelessness of clients with serious and persistent mental illness: Findings from a randomized trial of two ACT programs vs. usual care. *Mental Health Services Research,**2*(3), 155–164. 10.1023/a:1010141826867 11256724 10.1023/a:1010141826867

[CR33] Clark, R. E., Teague, G. B., Ricketts, S. K., Bush, P. W., Xie, H., McGuire, T. G., Drake, R. E., McHugo, G. J., Keller, A. M., & Zubkoff, M. (1998). Cost-effectiveness of assertive community treatment versus standard case management for persons with co-occurring severe mental illness and substance use disorders. *Health Services Research,**33*(5 Pt 1), 1285–1308. 9865221 PMC1070317

[CR36] Clausen, H., Landheim, A., Odden, S., Šaltytė Benth, J., Heiervang, K. S., Stuen, H. K., Killaspy, H., & Ruud, T. (2016a). Hospitalization of high and low inpatient service users before and after enrollment into Assertive Community Treatment teams: A naturalistic observational study. *International Journal of Mental Health Systems,**10*, 14. 26933446 10.1186/s13033-016-0052-z PMC4772328

[CR35] Clausen, H., Landheim, A., Odden, S., Heiervang, K. S., Stuen, H. K., Killaspy, H., Šaltytė Benth, J., & Ruud, T. (2015). Associations between quality of life and functioning in an Assertive Community Treatment population. *Psychiatric Services,**66*(11), 1249–1252. 10.1176/appi.ps.201400376 26234328 10.1176/appi.ps.201400376

[CR37] Clausen, H., Ruud, T., Odden, S., Šaltytė Benth, J., Heiervang, K. S., Stuen, H. K., Killaspy, H., Drake, R. E., & Landheim, A. (2016b). Hospitalisation of severely mentally ill patients with and without problematic substance use before and during Assertive Community Treatment: An observational cohort study. *BMC Psychiatry,**16*, 125. 27145937 10.1186/s12888-016-0826-5 PMC4855443

[CR38] Clausen, H., Ruud, T., Odden, S., Benth, J. Š, Heiervang, K. S., Stuen, H. K., & Landheim, A. (2020). Improved rehabilitation outcomes for persons with and without problematic substance use after 2 years with assertive community treatment-A prospective study of patients with severe mental illness in 12 Norwegian ACT teams. *Frontiers in Psychiatry,**11*, Article 607071. 10.3389/fpsyt.2020.607071 33424668 10.3389/fpsyt.2020.607071 PMC7785822

[CR39] Coetzee, D., Koen, L., Niehaus, D., & Botha, U. (2022). Descriptive outcomes for a cohort of high-frequency psychiatric service users in the Western Cape, South Africa after 10 years. *The South African journal of psychiatry : SAJP : the journal of the Society of Psychiatrists of South Africa,**28*, Article 1821. 10.4102/sajpsychiatry.v28i0.1821 35747340 10.4102/sajpsychiatry.v28i0.1821 PMC9210150

[CR40] Coldwell, C. M., & Bender, W. S. (2007). The effectiveness of assertive community treatment for homeless populations with severe mental illness: A meta-analysis. *American Journal of Psychiatry,**164*(3), 393–399. 10.1176/ajp.2007.164.3.393 17329462 10.1176/ajp.2007.164.3.393

[CR41] Commander, M., Sashidharan, S., Rana, T., & Ratnayake, T. (2005). North Birmingham assertive outreach evaluation. Patient characteristics and clinical outcomes. *Social Psychiatry and Psychiatric Epidemiology,**40*(12), 988–993. 10.1007/s00127-005-0989-8 16341614 10.1007/s00127-005-0989-8

[CR42] Corporation for Digital Scholarship. (2023). *Zotero (Version 6.0) [Computer software]*. Roy Rosenzweig Center for History and New Media. https://www.zotero.org

[CR43] Cusack, K. J., Morrissey, J. P., Cuddeback, G. S., Prins, A., & Williams, D. M. (2010). Criminal justice involvement, behavioral health service use, and costs of forensic assertive community treatment: A randomized trial. *Community Mental Health Journal,**46*(4), 356–363. 10.1007/s10597-010-9299-z 20217230 10.1007/s10597-010-9299-z PMC2895013

[CR44] Dahlan, R., Midin, M., & Sidi, H. (2013). Remission of symptoms among schizophrenia patients receiving assertive community treatment (ACT) in Malaysia: One year follow-up. *Sains Malaysiana*, *42*(3), 389–397.

[CR45] Daubney, M. F. X., Raeburn, N., Blackman, K., Jeffries, H., & Healy, K. L. (2021). Outcomes of assertive community treatment for adolescents with complex mental health problems who are difficult to engage. *Journal of Child and Family Studies,**30*(2), 502–516. 10.1007/s10826-020-01882-3

[CR46] de Bruijn, E., Jochems, E. C., Wierdsma, A. I., & Voskes, Y. (2023). The overlooked part of flexible assertive community treatment-A retrospective study on factors related to discharge from FACT for clients with a psychotic disorder. *Community Mental Health Journal,**59*(7), 1313–1320. 10.1007/s10597-023-01115-z 37086300 10.1007/s10597-023-01115-z PMC10447266

[CR48] Dekker, J., Wijdenes, W., Koning, Y. A., Gardien, R., Hermandes-Willenborg, L., & Nusselder, H. (2002). Assertive community treatment in Amsterdam. *Community Mental Health Journal,**38*(5), 425–434. 10.1023/a:1019816613834 12236412 10.1023/a:1019816613834

[CR47] de Pérez León, X. Y., Amodei, N., Hoffman, T. J., Martinez, R., Trevino, M., & Medina, D. (2011). Real world implementation of an adapted ACT model with minority and non minority homeless men. *International Journal of Mental Health and Addiction,**9*(6), 591–605. 10.1007/s11469-010-9287-0

[CR49] Dieterich, M., Irving, C. B., Bergman, H., Khokhar, M. A., Park, B., & Marshall, M. (2017). Intensive case management for severe mental illness. *Cochrane Database of Systematic Reviews,**2017*(1), Article CD007906. 10.1002/14651858.CD007906.pub3 10.1002/14651858.CD007906.pub3 PMC6472672 28067944

[CR50] Dincin, J., Wasmer, D., Witheridge, T. F., Sobeck, L., Cook, J., & Razzano, L. (1993). Impact of assertive community treatment on the use of state hospital inpatient bed-days. *Psychiatric Services,**44*(9), 833–838. 10.1176/ps.44.9.833 10.1176/ps.44.9.833 8225294

[CR51] Dixon, L., Weiden, P., Torres, M., & Lehman, A. (1997). Assertive community treatment and medication compliance in the homeless mentally ill. *American Journal of Psychiatry,**154*(9), 1302–1304. 10.1176/ajp.154.9.1302 9286194 10.1176/ajp.154.9.1302

[CR52] Donald, E. E., & Stajduhar, K. I. (2019). A scoping review of palliative care for persons with severe persistent mental illness. *Palliative & Supportive Care,**17*(4), 479–487. 10.1017/S1478951519000087 30887934 10.1017/S1478951519000087

[CR53] Doré-Gauthier, V., Miron, J. P., Jutras-Aswad, D., Ouellet-Plamondon, C., & Abdel-Baki, A. (2020). Specialized assertive community treatment intervention for homeless youth with first episode psychosis and substance use disorder: A 2-year follow-up study. *Early Intervention in Psychiatry,**14*(2), 203–210. 10.1111/eip.12846 31274239 10.1111/eip.12846

[CR54] Drake, R. E., McHugo, G. J., Clark, R. E., Teague, G. B., Xie, H., Miles, K., & Ackerson, T. H. (1998). Assertive community treatment for patients with co-occurring severe mental illness and substance use disorder: A clinical trial. *American Journal of Orthopsychiatry,**68*(2), 201–215. 10.1037/h0080330 9589759 10.1037/h0080330

[CR56] Drukker, M., Laan, W., Dreef, F., Driessen, G., Smeets, H., & Van Os, J. (2014). Can assertive community treatment remedy patients dropping out of treatment due to fragmented services? *Community Mental Health Journal,**50*(4), 454–459. 10.1007/s10597-013-9652-0 24178633 10.1007/s10597-013-9652-0

[CR55] Drukker, M., van Os, J., Sytema, S., Driessen, G., Visser, E., & Delespaul, P. (2011). Function assertive community treatment (FACT) and psychiatric service use in patients diagnosed with severe mental illness. *Epidemiology and Psychiatric Sciences,**20*(3), 273–278. 10.1017/s2045796011000369 21922970 10.1017/s2045796011000369

[CR57] Essock, S. M., Frisman, L. K., & Kontos, N. J. (1998). Cost-effectiveness of assertive community treatment teams. *American Journal of Orthopsychiatry,**68*(2), 179–190. 10.1037/h0080328 9589757 10.1037/h0080328

[CR58] Essock, S. M., Mueser, K. T., Drake, R. E., Covell, N. H., McHugo, G. J., Frisman, L. K., Kontos, N. J., Jackson, C. T., Townsend, F., & Swain, K. (2006). Comparison of ACT and standard case management for delivering integrated treatment for co-occurring disorders. *Psychiatric Services (Washington, D.C.),**57*(2), 185–196. 10.1176/appi.ps.57.2.185 16452695 10.1176/appi.ps.57.2.185

[CR59] Fam, J., Lee, C., Lim, B. L., & Lee, K. K. (2007). Assertive Community Treatment (ACT) in Singapore: A 1-year follow-up study. *Annals of the Academy of Medicine, Singapore,**36*(6), 409–412. 17597965

[CR60] Fekete, D. M., Bond, G. R., McDonel, E. C., Salyers, M., Chen, A., & Miller, L. (1998). Rural assertive community treatment: A field experiment. *Psychiatric Rehabilitation Journal,**21*(4), 371–379. 10.1037/h0095286

[CR61] Firn, M., Hindhaugh, K., Hubbeling, D., Davies, G., Jones, B., & White, S. J. (2013). A dismantling study of assertive outreach services: Comparing activity and outcomes following replacement with the FACT model. *Social Psychiatry and Psychiatric Epidemiology,**48*(6), 997–1003. 10.1007/s00127-012-0602-x 23086585 10.1007/s00127-012-0602-x

[CR62] Firn, M., White, S. J., Hubbeling, D., & Jones, B. (2016). The replacement of assertive outreach services by reinforcing local community teams: A four-year observational study. *Journal of Mental Health,**27*(1), 4–9. 10.3109/09638237.2016.1139073 26850124 10.3109/09638237.2016.1139073

[CR63] Gold, P. B., Jones, D. R., Macias, C., Bickman, L., Hargreaves, W. A., & Frey, J. (2012). A four-year retrospective study of Assertive Community Treatment: Change to more frequent, briefer client contact. *Bulletin of the Menninger Clinic,**76*(4), 314–328. 10.1521/bumc.2012.76.4.314 23244525 10.1521/bumc.2012.76.4.314 PMC4636011

[CR64] Goulet, M.-H., Dellazizzo, L., Lessard-Deschênes, C., Lesage, A., Crocker, A. G., & Dumais, A. (2022). Effectiveness of forensic assertive community treatment on forensic and health outcomes: A systematic review and meta-analysis. *Criminal Justice and Behavior,**49*(6), 838–852. 10.1177/00938548211061489

[CR65] Hackman, A. L., & Stowell, K. R. (2009). Transitioning clients from assertive community treatment to traditional mental health services. *Community Mental Health Journal,**45*(1), 1–5. 10.1007/s10597-008-9179-y 19130222 10.1007/s10597-008-9179-y

[CR66] Hambridge, J. A., & Rosen, A. (1994). Assertive community treatment for the seriously mentally ill in suburban Sydney: A programme description and evaluation. *Australian and New Zealand Journal of Psychiatry,**28*(3), 438–445. 10.3109/00048679409075871 7893238 10.3109/00048679409075871

[CR67] Hamernik, E., & Pakenham, K. I. (1999). Assertive community treatment for persons with severe mental disorders: A controlled treatment outcome study. *Behaviour Change,**16*(4), 259–268. 10.1375/bech.16.4.259

[CR68] Hamilton, I., Lloyd, C., Bland, J. M., & Savage Grainge, A. (2015). The impact of assertive outreach teams on hospital admissions for psychosis: A time series analysis. *Journal of Psychiatric and Mental Health Nursing,**22*(7), 484–490. 10.1111/jpm.12239 26118395 10.1111/jpm.12239

[CR69] Hansen, H. G., Starzer, M., Nilsson, S. F., Hjorthøj, C., Albert, N., & Nordentoft, M. (2023). Clinical recovery and long-term association of specialized early intervention services vs treatment as usual among individuals with first-episode schizophrenia spectrum disorder: 20-year follow-up of the OPUS trial. *JAMA Psychiatry,**80*(4), 371–379. 10.1001/jamapsychiatry.2022.5164 36811902 10.1001/jamapsychiatry.2022.5164 PMC9947803

[CR70] Harvey, C., Killaspy, H., Martino, S., White, S., Priebe, S., Wright, C., & Johnson, S. (2011). A comparison of the implementation of assertive community treatment in Melbourne, Australia and London, England. *Epidemiology and Psychiatric Sciences,**20*(2), 151–161. 10.1017/s2045796011000230 21714362 10.1017/s2045796011000230

[CR71] Hastrup, L. H., & Aagaard, J. (2015). Costs and outcome of assertive community treatment (ACT) in a rural area in Denmark: 4-year register-based follow-up. *Nordic Journal of Psychiatry,**69*(2), 110–117. 10.3109/08039488.2014.936500 25131794 10.3109/08039488.2014.936500

[CR72] Hastrup, L. H., Kronborg, C., Bertelsen, M., Jeppesen, P., Jorgensen, P., Petersen, L., Thorup, A., Simonsen, E., & Nordentoft, M. (2013). Cost-effectiveness of early intervention in first-episode psychosis: Economic evaluation of a randomised controlled trial (the OPUS study). *British Journal of Psychiatry,**202*(1), 35–41. 10.1192/bjp.bp.112.112300 10.1192/bjp.bp.112.112300 23174515

[CR73] Herdelin, A. C., & Scott, D. L. (1999). Experimental studies of the program of assertive community treatment (PACT): A meta-analysis. *Journal of Disability Policy Studies,**10*(1), 53–89. 10.1177/104420739901000105

[CR74] Herinckx, H. A., Kinney, R. F., Clarke, G. N., & Paulson, R. I. (1997). Assertive community treatment versus usual care in engaging and retaining clients with severe mental illness. *Psychiatric Services (Washington, D.C.),**48*(10), 1297–1306. 10.1176/ps.48.10.1297 9323749 10.1176/ps.48.10.1297

[CR75] Horiuchi, K., Nisihio, M., Oshima, I., Ito, J., Matsuoka, H., & Tsukada, K. (2006). The quality of life among persons with severe mental illness enrolled in an assertive community treatment program in Japan: 1-year follow-up and analyses. *Clinical Practice and Epidemiology in Mental Health : CP & EMH,**2*, Article 18. 10.1186/1745-0179-2-18 16875508 10.1186/1745-0179-2-18 PMC1550713

[CR76] Huguelet, P., Koellner, V., Boulguy, S., Nagalingum, K., Amani, S., Borras, L., & Perroud, N. (2012). Effects of an assertive community program in patients with severe mental disorders and impact on their families. *Psychiatry and Clinical Neurosciences,**66*(4), 328–336. 10.1111/j.1440-1819.2012.02337.x 22624738 10.1111/j.1440-1819.2012.02337.x

[CR77] Ito, J., Oshima, I., Nishio, M., & Kuno, E. (2009). Initiative to build a community-based mental health system including assertive community treatment for people with severe mental illness in Japan. *American Journal of Psychiatric Rehabilitation,**12*(3), 247–260. 10.1080/15487760903066404

[CR78] Ito, J., Oshima, I., Nishio, M., Sono, T., Suzuki, Y., Horiuchi, K., Niekawa, N., Ogawa, M., Setoya, Y., Hisanaga, F., Kouda, M., & Tsukada, K. (2011). The effect of Assertive Community Treatment in Japan. *Acta Psychiatrica Scandinavica,**123*(5), 398–401. 10.1111/j.1600-0447.2010.01636.x 21070193 10.1111/j.1600-0447.2010.01636.x

[CR79] Jerrell, J. M. (1995). Toward managed care for persons with severe mental illness: Implications from a cost-effectiveness study. *Health Affairs (Project Hope),**14*(3), 197–207. 10.1377/hlthaff.14.3.197 7498892 10.1377/hlthaff.14.3.197

[CR80] Jones, A. (2002). Assertive community treatment: Development of the team, selection of clients, and impact on length of hospital stay. *Journal of Psychiatric and Mental Health Nursing,**9*, 261–270. 10.1046/j.1365-2850.2002.00465.x 12060369 10.1046/j.1365-2850.2002.00465.x

[CR81] Jørgensen, P., Nordentoft, M., Abel, M. B., Gouliaev, G., Jeppesen, P., & Kassow, P. (2000). Early detection and assertive community treatment of young psychotics: The Opus Study Rationale and design of the trial. *Social Psychiatry and Psychiatric Epidemiology,**35*(7), 283–287. 10.1007/s001270050240 11016522 10.1007/s001270050240

[CR82] Kane, C. F., & Blank, M. B. (2004). NPACT: Enhancing programs of assertive community treatment for the seriously mentally ill. *Community Mental Health Journal,**40*(6), 549–559. 10.1007/s10597-004-6128-2 15672693 10.1007/s10597-004-6128-2

[CR84] Karow, A., Brettschneider, C., Helmut König, H., Correll, C. U., Schöttle, D., Lüdecke, D., Rohenkohl, A., Ruppelt, F., Kraft, V., Gallinat, J., & Lambert, M. (2020). Better care for less money: Cost-effectiveness of integrated care in multi-episode patients with severe psychosis. *Acta Psychiatrica Scandinavica,**141*(3), 221–230. 10.1111/acps.13139 31814102 10.1111/acps.13139

[CR83] Karow, A., Reimer, J., König, H. H., Heider, D., Bock, T., Huber, C., Schöttle, D., Meister, K., Rietschel, L., Ohm, G., Schulz, H., Naber, D., Schimmelmann, B. G., & Lambert, M. (2012). Cost-effectiveness of 12-month therapeutic assertive community treatment as part of integrated care versus standard care in patients with schizophrenia treated with quetiapine immediate release (ACCESS trial). *The Journal of Clinical Psychiatry,**73*(3), e402–e408. 10.4088/JCP.11m06875 22490266 10.4088/JCP.11m06875

[CR85] Kenny, D. A., Calsyn, R. J., Morse, G. A., Klinkenberg, W. D., Winter, J. P., & Trusty, M. L. (2004). Evaluation of treatment programs for persons with severe mental illness: Moderator and mediator effects. *Evaluation Review,**28*(4), 294–324. 10.1177/0193841X04264701 15245622 10.1177/0193841X04264701

[CR86] Killaspy, H., Bebbington, P., Blizard, R., Johnson, S., Nolan, F., Pilling, S., & King, M. (2006). The REACT study: Randomised evaluation of assertive community treatment in north London. *BMJ,**332*(7545), 815–820. 10.1136/bmj.38773.518322.7C 16543298 10.1136/bmj.38773.518322.7C PMC1432213

[CR87] Killaspy, H., Kingett, S., Bebbington, P., Blizard, R., Johnson, S., Nolan, F., Pilling, S., & King, M. (2009). Randomised evaluation of assertive community treatment: 3-year outcomes. *The British Journal of Psychiatry : the Journal of Mental Science,**195*(1), 81–82. 10.1192/bjp.bp.108.059303 19567902 10.1192/bjp.bp.108.059303

[CR88] Killaspy, H., Mas-Expósito, L., Marston, L., & King, M. (2014). Ten year outcomes of participants in the REACT (Randomised Evaluation of Assertive Community Treatment in North London) study. *BMC Psychiatry,**14*, Article 296. 10.1186/s12888-014-0296-6 25342641 10.1186/s12888-014-0296-6 PMC4210468

[CR89] Kim, T. W., Jeong, J. H., Kim, Y. H., Kim, Y., Seo, H. J., & Hong, S. C. (2015). Fifteen-month follow up of an assertive community treatment program for chronic patients with mental illness. *BMC Health Services Research,**15*, Article 388. 10.1186/s12913-015-1058-y 26376978 10.1186/s12913-015-1058-y PMC4573673

[CR92] Kortrijk, H. E., Kamperman, A. M., & Mulder, C. L. (2014). Changes in individual needs for care and quality of life in Assertive Community Treatment patients: An observational study. *BMC Psychiatry,**14*, Article 306. 10.1186/s12888-014-0306-8 25403357 10.1186/s12888-014-0306-8 PMC4236799

[CR90] Kortrijk, H. E., Mulder, C. L., Roosenschoon, B. J., & Wiersma, D. (2010). Treatment outcome in patients receiving assertive community treatment. *Community Mental Health Journal,**46*(4), 330–336. 10.1007/s10597-009-9257-9 19847646 10.1007/s10597-009-9257-9 PMC2910892

[CR91] Kortrijk, H. E., Mulder, C. L., van der Gaag, M., & Wiersma, D. (2012). Symptomatic and functional remission and its associations with quality of life in patients with psychotic disorder in Assertive Community Treatment teams. *Comprehensive Psychiatry,**53*(8), 1174–1180. 10.1016/j.comppsych.2012.05.001 22738674 10.1016/j.comppsych.2012.05.001

[CR93] Kortrijk, H., Schaefer, B., van Weeghel, J., Mulder, C. L., & Kamperman, A. (2019). Trajectories of patients with severe mental illness in two-year contact with Flexible Assertive Community Treatment teams using Routine Outcome Monitoring data: An observational study. *PLoS One,**14*(1), Article e0207680. 10.1371/journal.pone.0207680 30625133 10.1371/journal.pone.0207680 PMC6326457

[CR94] Kreindler, S. A., & Coodin, S. (2010). Housing histories of assertive community treatment clients: Program impacts and factors associated with residential stability. *Canadian Journal of Psychiatry,**55*(3), 150–156. 10.1177/070674371005500306 20370965 10.1177/070674371005500306

[CR95] Lafave, H. G., de Souza, H. R., & Gerber, G. J. (1996). Assertive community treatment of severe mental illness: A Canadian experience. *Psychiatric Services (Washington, D.C.),**47*(7), 757–759. 10.1176/ps.47.7.757 8807692 10.1176/ps.47.7.757

[CR97] Lamberti, J. S., Weisman, R. L., Cerulli, C., Williams, G. C., Jacobowitz, D. B., Mueser, K. T., Marks, P. D., Strawderman, R. L., Harrington, D., Lamberti, T. A., & Caine, E. D. (2017a). A randomized controlled trial of the Rochester Forensic Assertive Community Treatment Model. *Psychiatric Services,**68*(10), 1016–1024. 10.1176/appi.ps.201600329 28566028 10.1176/appi.ps.201600329 PMC7369621

[CR98] Lamberti, J. S., Weisman, R. L., Schwarzkopf, S. B., Price, N., Ashton, R. M., Trompeter, J., & Waford, R. N. (2017b). The role of probation in forensic assertive community treatment. *Psychiatric Services,**68*(4), 374–380. 10.1176/appi.ps.201600150 10.1176/appi.ps.62.4.418 PMC3098459 21459994

[CR96] Lambert, M., Bock, T., Schöttle, D., Golks, D., Meister, K., Rietschel, L., Bussopulos, A., Frieling, M., Schödlbauer, M., Burlon, M., Huber, C. G., Ohm, G., Pakrasi, M., Chirazi-Stark, M. S., Naber, D., & Schimmelmann, B. G. (2010). Assertive community treatment as part of integrated care versus standard care: A 12-month trial in patients with first- and multiple-episode schizophrenia spectrum disorders treated with quetiapine immediate release (ACCESS trial). *Journal of Clinical Psychiatry,**71*(10), 1313–1323. 10.4088/JCP.09m05113yel 20361911 10.4088/JCP.09m05113yel

[CR99] Lang, M., Davidson, L., Bailey, P., & Levine, M. (1999). Clinicians’ and clients’ perspectives on the impact of assertive community treatment. *Psychiatric Services,**50*(10), 1331–1340. 10.1176/ps.50.10.1331 10506303 10.1176/ps.50.10.1331

[CR100] Latimer, E. A., Rabouin, D., Cao, Z., Ly, A., Powell, G., Aubry, T., Distasio, J., Hwang, S. W., Somers, J. M., Bayoumi, A. M., Mitton, C., Moodie, E. E. M., Goering, P. N., At Home/Chez Soi Investigators. (2020). Cost-effectiveness of housing first with assertive community treatment: Results from the Canadian At Home/Chez Soi Trial. *Psychiatric Services (Washington, D.C.),**71*(10), 1020–1030. 10.1176/appi.ps.202000029 32838679 10.1176/appi.ps.202000029

[CR101] Lee, C. C., Liem, S. K., Leung, J. S., Wong, K., Yuen, S. K., Lee, W. L., Lo, W. L., & Yip, N. H. (2015a). Assertive community treatment for psychiatric patients with frequent hospitalisation. *Hong Kong medical journal = Xianggang yi xue za zhi*, *21 Suppl 2*, 37–40. 25852101

[CR102] Lee, C. C., Liem, S. K., Leung, J., Young, V., Wu, K., Wong, K. K., Yuen, S. K., Lee, W. F., Leung, T., Shum, M., Kwong, P., & Lo, W. (2015b). From deinstitutionalization to recovery-oriented assertive community treatment in Hong Kong: What we have achieved. *Psychiatry Research*, *228*(3), 243–250. ttps://doi.org/10.1016/j.psychres.2015.05.106 26168932 10.1016/j.psychres.2015.05.106

[CR103] Lehman, A. F., Dixon, L. B., Kernan, E., DeForge, B. R., & Postrado, L. T. (1997). A randomized trial of assertive community treatment for homeless persons with severe mental illness. *Archives Of General Psychiatry,**54*(11), 1038–1043. 10.1001/archpsyc.1997.01830230076011 9366661 10.1001/archpsyc.1997.01830230076011

[CR104] Lehman, A. F., Dixon, L., Hoch, J. S., Deforge, B., Kernan, E., & Frank, R. (1999). Cost-effectiveness of assertive community treatment for homeless persons with severe mental illness. *The British Journal Of Psychiatry : The Journal Of Mental Science,**174*, 346–352. 10.1192/bjp.174.4.346 10533554 10.1192/bjp.174.4.346

[CR105] Liem, S. K., & Lee, C. C. (2013). Effectiveness of assertive community treatment in Hong Kong among patients with frequent hospital admissions. *Psychiatric Services (Washington, D.C.),**64*(11), 1170–1172. 10.1176/appi.ps.201200421 24185540 10.1176/appi.ps.201200421

[CR107] Low, L., Tan, Y. Y., Lim, B. L., Poon, W. C., & Lee, C. (2013). Effectiveness of assertive community management in Singapore. *Annals of the Academy of Medicine, Singapore,**42*(3), 125–132. 23604501

[CR106] López-Santiago, J., Blas, L. V., & Gómez, M. (2012). Effectiveness of an assertive community treatment program for patients with severe mental disorder. *Revista De Psicopatología Y Psicología Clínica,**17*(1), 1–10. 10.5944/rppc.vol.17.num.1.2012.10365

[CR108] Luo, X., Law, S. F., Wang, X., Shi, J., Zeng, W., Ma, X., Chow, W., Liu, S., Zhao, W., Liu, X., Yao, S., & Phillips, M. R. (2019). Effectiveness of an Assertive Community Treatment program for people with severe schizophrenia in mainland China - a 12-month randomized controlled trial. *Psychological Medicine,**49*(6), 969–979. 10.1017/S0033291718001629 29962366 10.1017/S0033291718001629

[CR109] Mantzouranis, G., Baier, V., Holzer, L., Urben, S., & Villard, E. (2019). Clinical significance of assertive community treatment among adolescents. *Social Psychiatry and Psychiatric Epidemiology,**54*(4), 445–453. 10.1007/s00127-018-1613-z 30310946 10.1007/s00127-018-1613-z

[CR110] Manuel, J. I., Covell, N. H., Jackson, C. T., & Essock, S. M. (2011). Does assertive community treatment increase medication adherence for people with co-occurring psychotic and substance use disorders? *Journal of the American Psychiatric Nurses Association,**17*(1), 51–56. 10.1177/1078390310395586 21659294 10.1177/1078390310395586 PMC4164343

[CR111] Marquant, T., Sabbe, B., van Nuffel, M., & Goethals, K. (2018a). Characteristics of offenders with schizophrenia spectrum disorders in forensic psychiatry: A review. *International Journal of Law and Psychiatry*, *57*, 40–51. ttps://doi.org/10.1016/j.ijlp.2018.01.004

[CR112] Marquant, T., Sabbe, B., Van Nuffel, M., Verelst, R., & Goethals, K. (2018b). Forensic Assertive Community Treatment in a continuum of care for male internees in Belgium: Results after 33 months. *Community Mental Health Journal,**54*(1), 58–65. 28791496 10.1007/s10597-017-0153-4

[CR113] Marx, A. J., Test, M. A., & Stein, L. I. (1973). Extrohospital management of severe mental illness: Feasibility and effects on social functioning. *Archives of General Psychiatry,**29*(4), 505–511. 10.1001/archpsyc.1973.01750340033005 4748311 10.1001/archpsyc.1973.04200040051009

[CR114] McCrone, P., Killaspy, H., Bebbington, P., Johnson, S., Nolan, F., Pilling, S., & King, M. (2009). The REACT study: Cost-effectiveness analysis of assertive community treatment in north London. *Psychiatric Services (Washington, D.C.),**60*(7), 908–913. 10.1176/ps.2009.60.7.908 19564220 10.1176/ps.2009.60.7.908

[CR115] McFarlane, W. R., Dushay, R. A., Stastny, P., Deakins, S. M., & Link, B. (1996). A comparison of two levels of family-aided assertive community treatment. *Psychiatric Services (Washington, D.C.),**47*(7), 744–750. 10.1176/ps.47.7.744 8807689 10.1176/ps.47.7.744

[CR116] McGrew, J. H., Bond, G. R., Dietzen, L., McKasson, M., & Miller, L. D. (1995). A multisite study of client outcomes in assertive community treatment. *Psychiatric Services (Washington, D.C.),**46*(7), 696–701. 10.1176/ps.46.7.696 7552561 10.1176/ps.46.7.696

[CR117] McGuire, J. F., & Rosenheck, R. A. (2004). Criminal history as a prognostic indicator in the treatment of homeless people with severe mental illness. *Psychiatric Services (Washington, D.C.),**55*(1), 42–48. 10.1176/appi.ps.55.1.42 14699199 10.1176/appi.ps.55.1.42

[CR118] McHugo, G. J., Drake, R. E., Teague, G. B., & Xie, H. (1999). Fidelity to assertive community treatment and client outcomes in the New Hampshire dual disorders study. *Psychiatric Services,**50*(6), 818–824. 10.1176/ps.50.6.818 10375153 10.1176/ps.50.6.818

[CR119] Meisler, N., Blankertz, L., Santos, A. B., & McKay, C. (1997). Impact of assertive community treatment on homeless persons with co-occurring severe psychiatric and substance use disorders. *Community Mental Health Journal,**33*(2), 113–122. 10.1023/a:1022419316396 9145253 10.1023/a:1022419316396

[CR120] Mental Health Coordinating Council. (2022). *Recovery oriented language guide* (3rd ed.). Mental Health Coordinating Council.

[CR121] Microsoft Corporation (2018). Microsoft Excel (Version 16.0). Microsoft. https://office.microsoft.com/excel

[CR122] Mohamed, S. (2016). Comparison of intensive case management for psychotic and nonpsychotic patients. *Psychological Services,**13*(1), 10–19. 10.1037/ser0000041 26168139 10.1037/ser0000041

[CR123] Mohamed, S., Rosenheck, R., & Cuerdon, T. (2010). Who terminates from ACT and why? Data from the National VA Mental Health Intensive Case Management Program. *Psychiatric Services (Washington, D.C.),**61*(7), 675–683. 10.1176/ps.2010.61.7.675 20592002 10.1176/ps.2010.61.7.675

[CR124] Monroe-DeVita, M., Teague, G. B., & Moser, L. L. (2011). The TMACT: A new tool for measuring fidelity to assertive community treatment. *Journal of the American Psychiatric Nurses Association,**17*(1), 17–29. 21659289 10.1177/1078390310394658

[CR126] Morrissey, J. P., Domino, M. E., & Cuddeback, G. S. (2013). Assessing the effectiveness of recovery-oriented ACT in reducing state psychiatric hospital use. *Psychiatric Services,**64*(4), 303–311. 10.1176/appi.ps.201200095 23242485 10.1176/appi.ps.201200095

[CR125] Morrissey, J. P., Meyer, P. S., & Cuddeback, G. S. (2007). Extending assertive community treatment to criminal justice settings: Origins, current evidence, and future directions. *Community Mental Health Journal,**43*(5), 527–544. 10.1007/s10597-007-9092-9 17587178 10.1007/s10597-007-9092-9

[CR127] Morse, G. A., Calsyn, R. J., Klinkenberg, W. D., Trusty, M. L., Gerber, F., Smith, R., Tempelhoff, B., & Ahmad, L. (1997). An experimental comparison of three types of case management for homeless mentally ill persons. *Psychiatric Services (Washington, D.C.),**48*(4), 497–503. 10.1176/ps.48.4.497 9090733 10.1176/ps.48.4.497

[CR128] Mowbray, C. T., Collins, M. E., Plum, T. B., Masterton, T., & Mulder, R. (1997). Harbinger. I: The development and evaluation of the first PACT replication. *Administration and Policy in Mental Health,**25*(2), 105–123. 10.1023/a:1022230803615 9727211 10.1023/a:1022230803615

[CR129] Mueser, K. T., Bond, G. R., Drake, R. E., & Resnick, S. G. (1998). Models of community care for severe mental illness: A review of research on case management. *Schizophrenia Bulletin,**24*(1), 37–74. 10.1093/oxfordjournals.schbul.a033314 9502546 10.1093/oxfordjournals.schbul.a033314

[CR130] Munn, Z., Peters, M. D. J., Stern, C., Tufanaru, C., McArthur, A., & Aromataris, E. (2018). Systematic review or scoping review? Guidance for authors when choosing between a systematic or scoping review approach. *BMC Medical Research Methodology,**18*(1), Article 143. 10.1186/s12874-018-0611-x 30453902 10.1186/s12874-018-0611-x PMC6245623

[CR131] National Institute of Mental Health. (1987). *Toward a model plan for a comprehensive, community-based mental health system*. U.S. Department of Health and Human Services, Public Health Service, Alcohol, Drug Abuse, and Mental Health Administration.

[CR133] Nielsen, C., Hjorthøj, C., Arnfred, B. T., & Nordentoft, M. (2023). Patient outcomes of flexible assertive community treatment compared with assertive community treatment. *Psychiatric Services (Washington, D.C.),**74*(7), 695–701. 10.1176/appi.ps.20220235 36475824 10.1176/appi.ps.20220235

[CR132] Nielsen, C. M., Hjorthøj, C., Killaspy, H., & Nordentoft, M. (2021). The effect of flexible assertive community treatment in Denmark: A quasi-experimental controlled study. *The Lancet. Psychiatry,**8*(1), 27–35. 10.1016/S2215-0366(20)30424-7 33091344 10.1016/S2215-0366(20)30424-7

[CR134] Nieves, E. J. (2002). The effectiveness of the assertive community treatment model. *Administration and Policy in Mental Health,**29*(6), 461–480. 10.1023/a:1020724326260 12469701 10.1023/a:1020724326260

[CR135] Nishio, M., Ito, J., Oshima, I., Suzuki, Y., Horiuchi, K., Sono, T., Fukaya, H., Hisanaga, F., & Tsukada, K. (2012). Preliminary outcome study on assertive community treatment in Japan. *Psychiatry and Clinical Neurosciences,**66*(5), 383–389. 10.1111/j.1440-1819.2012.02348.x 22834656 10.1111/j.1440-1819.2012.02348.x

[CR137] Nordentoft, M., Øhlenschlaeger, J., Thorup, A., Petersen, L., Jeppesen, P., & Bertelsen, M. (2010). Deinstitutionalization revisited: A 5-year follow-up of a randomized clinical trial of hospital-based rehabilitation versus specialized assertive intervention (OPUS) versus standard treatment for patients with first-episode schizophrenia spectrum disorders. *Psychological Medicine,**40*(10), 1619–1626. 10.1017/S0033291709992182 20059798 10.1017/S0033291709992182

[CR138] Nordentoft, M., Melau, M., Iversen, T., Petersen, L., Jeppesen, P., Thorup, A., Bertelsen, M., Hjorthøj, C. R., Hastrup, L. H., & Jørgensen, P. (2015a). From research to practice: How OPUS treatment was accepted and implemented throughout Denmark. *Early Intervention in Psychiatry,**9*(2), 156–162. 10.1111/eip.12108 24304658 10.1111/eip.12108

[CR139] Nordentoft, M., Rasmussen, J. Ø., Melau, M., Hjorthøj, C. R., & Thorup, A. A. (2015b). How successful are first episode programs? A review of the evidence for specialized assertive early intervention. *Current Opinion in Psychiatry,**27*(3), 167–172. 10.1097/YCO.0000000000000150 10.1097/YCO.0000000000000052 24662959

[CR136] Nordén, T., Malm, U., & Norlander, T. (2012). Resource group assertive community treatment (RACT) as a tool of empowerment for clients with severe mental illness: A meta-analysis. *Clinical Practice and Epidemiology in Mental Health,**8*, 144–151. 10.2174/1745017901208010144 23173010 10.2174/1745017901208010144 PMC3502888

[CR140] Nugter, M. A., Engelsbel, F., Bähler, M., Keet, R., & van Veldhuizen, R. (2016). Outcomes of FLEXIBLE Assertive Community Treatment (FACT) Implementation: A Prospective Real Life Study. *Community Mental Health Journal,**52*(8), 898–907. 10.1007/s10597-015-9831-2 25648552 10.1007/s10597-015-9831-2 PMC5108818

[CR141] OECD & European Union (2018). Health at a glance: Europe 2018: State of health in the EU cycle. OECD Publishing. https://www.oecd-ilibrary.org/social-issues-migration-health/health-at-a-glance-europe2018_health_glance_eur-2018-ensabizcon/es/

[CR142] Patel, V., Araya, R., Chatterjee, S., Chisholm, D., Cohen, A., De Silva, M., Hosman, C., McGuire, H., Rojas, G., & van Ommeren, M. (2007). Treatment and prevention of mental disorders in low-income and middle-income countries. *Lancet,**370*(9591), 991–1005. 10.1016/S0140-6736(07)61240-9 17804058 10.1016/S0140-6736(07)61240-9

[CR143] Penzenstadler, L., Soares, C., Anci, E., Molodynski, A., & Khazaal, Y. (2019). Effect of Assertive Community Treatment for Patients with Substance Use Disorder: A Systematic Review. *European Addiction Research,**25*(2), 56–67. 10.1159/000496742 30699412 10.1159/000496742

[CR145] Petersen, L., Jeppesen, P., Thorup, A., Abel, M. B., Øhlenschlaeger, J., Christensen, T. Ø., Krarup, G., Jørgensen, P., & Nordentoft, M. (2005). A randomised multicentre trial of integrated versus standard treatment for patients with a first episode of psychotic illness. *BMJ (Clinical Research Ed.),**331*(7517), 602. 10.1136/bmj.38565.415000.E01 16141449 10.1136/bmj.38565.415000.E01 PMC1215551

[CR146] Petersen, L., Jeppesen, P., Thorup, A., Ohlenschlaeger, J., Krarup, G., Ostergård, T., Jørgensen, P., & Nordentoft, M. (2007). Substance abuse and first-episode schizophrenia-spectrum disorders. The Danish OPUS trial. *Early intervention in psychiatry*, *1*(1), 88–96. ttps://doi.org/10.1111/j.1751-7893.2007.00015.x 21352112 10.1111/j.1751-7893.2007.00015.x

[CR144] Peters, M., Godfrey, C., McInerney, P., Munn, Z., Tricco, A. C., & Khalil, H. (2020). Chapter 11: Scoping reviews. In E. Aromataris & Z. Munn (Eds.), JBI Manual for Evidence Synthesis. JBI. ttps://doi.org/10.46658/JBIRM-20-01

[CR147] Phillips, S. D., Burns, B. J., Edgar, E. R., Mueser, K. T., Linkins, K. W., Rosenheck, R. A., Drake, R. E., & McDonel Herr, E. C. (2001). Moving assertive community treatment into standard practice. *Psychiatric Services*, *52*(6), 771–779. ttps://doi.org/10.1176/appi.ps.52.6.771 11376224 10.1176/appi.ps.52.6.771

[CR148] Pina, I., Gilfellon, L., Webster, S., Henderson, E. J., & Oliver, E. J. (2024). Severe mental illness’: Uses of this term in physical health support policy, primary care practice, and academic discourses in the United Kingdom. *SSM Mental health*, *5*, 100314. ttps://doi.org/10.1016/j.ssmmh.2024.100314 38910841 10.1016/j.ssmmh.2024.100314 PMC11188150

[CR149] Pollock, D., Davies, E. L., Peters, M. D. J., Tricco, A. C., Alexander, L., McInerney, P., Godfrey, C. M., Khalil, H., & Munn, Z. (2021). Undertaking a scoping review: A practical guide for nursing and midwifery students, clinicians, researchers, and academics. *Journal of Advanced Nursing*, *77*(4), 2102–2113. ttps://doi.org/10.1111/jan.14743 33543511 10.1111/jan.14743 PMC8049063

[CR150] Pope, L. M., & Harris, G. E. (2014). Assertive community treatment (ACT) in a rural Canadian community: Client characteristics, client satisfaction, and service effectiveness. *Canadian Journal of Community Mental Health*, *33*(3), 17–27. ttps://doi.org/10.7870/cjcmh-2014-019

[CR151] Poremski, D., Stergiopoulos, V., Braithwaite, E., Distasio, J., Nisenbaum, R., & Latimer, E. (2016). Effects of Housing First on employment and income of homeless individuals: Results of a randomized trial. *Psychiatric Services*, *67*(6), 603–609. ttps://doi.org/10.1176/appi.ps.201500002 26876657 10.1176/appi.ps.201500002

[CR152] Prakash, J., Andrews, T., & Porter, I. (2007). Service innovation: Assertive outreach teams for adults with learning disability. *Psychiatric Bulletin*, *31*(4), 138–141. 10.1192/pb.bp.105.004648

[CR153] Psychiatric Rehabilitation Association (2003). PRA language guidelines. https://www.psychrehabassociation.org/pra-language-guidelines

[CR154] Randall, J. R., Vokey, S., Loewen, H., Martens, P. J., Brownell, M., Katz, A., Nickel, N. C., Burland, E., & Chateau, D. (2015). A systematic review of the effect of early interventions for psychosis on the usage of inpatient services. *Schizophrenia Bulletin*, *41*(6), 1379–1386. ttps://doi.org/10.1093/schbul/sbv016 25745034 10.1093/schbul/sbv016 PMC4601703

[CR155] Razali, S. M., & Hashim, M. A. (2015). Modified assertive community treatment: Effectiveness on hospitalization and length of stay. *Community mental health journal*, *51*(2), 171–174. ttps://doi.org/10.1007/s10597-014-9757-0 25056686 10.1007/s10597-014-9757-0

[CR156] Rezansoff, S. N., Moniruzzaman, A., Fazel, S., McCandless, L., Procyshyn, R., & Somers, J. M. (2017). Housing first improves adherence to antipsychotic medication among formerly homeless adults with schizophrenia: Results of a randomized controlled trial. *Schizophrenia Bulletin,**43*(4), 852–861. 10.1093/schbul/sbw136 27665002 10.1093/schbul/sbw136 PMC5274537

[CR157] Rochefort, D. A. (2019). Innovation and its discontents: Pathways and barriers in the diffusion of assertive community treatment. *The Milbank Quarterly,**97*(4), 1151–1199. 10.1111/1468-0009.12429 31680353 10.1111/1468-0009.12429 PMC6904263

[CR158] Rohenkohl, A. C., Daubmann, A., Gallinat, J., Karow, A., Kraft, V., Rühl, F., Schöttle, D., Lambert, M., & Schröter, R. (2022). Health-related quality of life in severe psychotic disorders during integrated care: 5-year course, prediction and treatment implications (ACCESS II). *Health and Quality of Life Outcomes,**20*(1), Article 133. 10.1186/s12955-022-02039-0 36076205 10.1186/s12955-022-02039-0 PMC9452858

[CR159] Salkever, D., Domino, M. E., Burns, B. J., Santos, A. B., Deci, P. A., Dias, J., Wagner, H. R., Faldowski, R. A., & Paolone, J. (1999). Assertive community treatment for people with severe mental illness: The effect on hospital use and costs. *Health Services Research,**34*(2), 577–601. 10357291 PMC1089024

[CR160] Salyers, M. P., Rollins, A. L., Clendenning, D., McGuire, A. B., & Kim, E. (2011). Impact of illness management and recovery programs on hospital and emergency room use by Medicaid enrollees. *Psychiatric Services,**62*(5), 509–515. 10.1176/ps.62.5.pss6205_0509 21532077 10.1176/appi.ps.62.5.509 PMC3093966

[CR161] Santos, A. B., Hawkins, G. D., Julius, B., Deci, P. A., Hiers, T. H., & Burns, B. J. (1993). A pilot study of assertive community treatment for patients with chronic psychotic disorders. *The American Journal of Psychiatry,**150*(3), 501–504. 10.1176/ajp.150.3.501 8434670 10.1176/ajp.150.3.501

[CR164] Schröter, R., Lambert, M., Rohenkohl, A., Kraft, V., Rühl, F., Luedecke, D., Gallinat, J., Karow, A., & Schmidt, S. J. (2022). Mediators of quality of life change in people with severe psychotic disorders treated in integrated care: ACCESS II study. *European Psychiatry,**66*(1), Article e1. 10.1192/j.eurpsy.2022.2332 36329654 10.1192/j.eurpsy.2022.2332 PMC9879911

[CR163] Schöttle, D., Ruppelt, F., Schimmelmann, B. G., Karow, A., Bussopulos, A., Gallinat, J., Wiedemann, K., Luedecke, D., Rohenkohl, A. C., Huber, C. G., Bock, T., & Lambert, M. (2019). Reduction of involuntary admissions in patients with severe psychotic disorders treated in the ACCESS integrated care model including therapeutic assertive community treatment. *Frontiers in Psychiatry,**10*, Article 736. 10.3389/fpsyt.2019.00736 31708810 10.3389/fpsyt.2019.00736 PMC6822062

[CR162] Schöttle, D., Schimmelmann, B. G., Ruppelt, F., Bussopulos, A., Frieling, M., Nika, E., Nawara, L. A., Golks, D., Kerstan, A., Lange, M., Schödlbauer, M., Daubmann, A., Wegscheider, K., Rohenkohl, A., Sarikaya, G., Sengutta, M., Luedecke, D., Wittmann, L., Ohm, G., … Lambert, M. (2018). Effectiveness of integrated care including therapeutic assertive community treatment in severe schizophrenia-spectrum and bipolar I disorders: Four-year follow-up of the ACCESS II study. *PLoS One,**13*(2), Article e0192929. 10.1371/journal.pone.0192929 29485988 10.1371/journal.pone.0192929 PMC5828355

[CR166] Siskind, D., Dark, F., Carney, K., Gore-Jones, V., Kar Ray, M., Steginga, A., Suetani, S., & Kisely, S. (2021). Placing rehabilitation at the core of assertive community treatment. *Australasian psychiatry : bulletin of Royal Australian and New Zealand College of Psychiatrists,**29*(1), 47–51. 10.1177/1039856220928876 32469640 10.1177/1039856220928876

[CR165] Siskind, D., & Wiley-Exley, E. (2009). Comparison of assertive community treatment programs in urban Massachusetts and rural North Carolina. *Administration and Policy in Mental Health,**36*(4), 236–246. 10.1007/s10488-009-0208-0 19214732 10.1007/s10488-009-0208-0

[CR167] Slade, E. P., McCarthy, J. F., Valenstein, M., Visnic, S., & Dixon, L. B. (2013). Cost savings from assertive community treatment services in an era of declining psychiatric inpatient use. *Health Services Research,**48*(1), 195–217. 10.1111/j.1475-6773.2012.01420.x 22594523 10.1111/j.1475-6773.2012.01420.x PMC3589962

[CR168] Smith, L., & Newton, R. (2007). Systematic review of case management. *Australian and New Zealand Journal of Psychiatry,**41*(1), 2–9. 10.1080/00048670601039831 17464675 10.1080/00048670601039831

[CR169] Smith, R. J., Jennings, J. L., & Cimino, A. (2010). Forensic continuum of care with Assertive Community Treatment (ACT) for persons recovering from co-occurring disabilities: Long-term outcomes. *Psychiatric Rehabilitation Journal,**33*(3), 207–218. 10.2975/33.3.2010.207.218 20061257 10.2975/33.3.2010.207.218

[CR170] Somers, J. M., Patterson, M. L., Moniruzzaman, A., Currie, L., Rezansoff, S. N., Palepu, A., & Fryer, K. (2013). Vancouver At Home: Pragmatic randomized trials investigating Housing First for homeless and mentally ill adults. *Trials,**14*, Article 365. 10.1186/1745-6215-14-365 24176253 10.1186/1745-6215-14-365 PMC4228396

[CR171] Sood, L., & Owen, A. (2014). A 10-year service evaluation of an assertive community treatment team: Trends in hospital bed use. *Journal of Mental Health,**23*(6), 323–327. 10.3109/09638237.2014.954694 25222169 10.3109/09638237.2014.954694

[CR172] Sood, L., Owen, A., Onyon, R., Sharma, A., Nigriello, J., Markham, D., & Seabrook, H. (2017). Flexible assertive community treatment (FACT) model in specialist psychosis teams: An evaluation. *BJPsych Bulletin,**41*(4), 192–196. 10.1192/pb.bp.116.053967 28811912 10.1192/pb.bp.116.053967 PMC5537572

[CR174] Stein, L. I., Barry, K. L., Van Dien, G., Hollingsworth, E. J., & Sweeney, J. K. (1999). Work and social support: A comparison of consumers who have achieved stability in ACT and clubhouse programs. *Community Mental Health Journal,**35*(2), 193–204. 10.1023/a:1018780916794 10412627 10.1023/a:1018780916794

[CR173] Stein, L. I., & Test, M. A. (1980). An alternative to mental health treatment. I: Conceptual model, treatment program, and clinical evaluation. *Archives of General Psychiatry,**37*(4), 392–397. 10.1001/archpsyc.1980.01780170034003 7362425 10.1001/archpsyc.1980.01780170034003

[CR175] Stergiopoulos, V., Hwang, S. W., Gozdzik, A., Nisenbaum, R., Latimer, E., Rabouin, D., Adair, C. E., Bourque, J., Connelly, J., Frankish, J. C., Katz, L. Y., Mason, K., Misir, V., O’Brien, K., Sareen, J., Schütz, C. G., Singer, A., Streiner, D. L., Vasiliadis, H. M., & Goering, P. N. (2015). Effect of scattered-site housing using rent supplements and intensive case management on housing stability among homeless adults with mental illness: A randomized trial. *JAMA,**313*(9), 905–915. 25734732 10.1001/jama.2015.1163

[CR176] Stobbe, J., Wierdsma, A. I., Kok, R. M., Kroon, H., Roosenschoon, B. J., Depla, M., & Mulder, C. L. (2014). The effectiveness of assertive community treatment for elderly patients with severe mental illness: A randomized controlled trial. *BMC Psychiatry,**14*, Article 42. 10.1186/1471-244X-14-42 24528604 10.1186/1471-244X-14-42 PMC3928976

[CR177] Svensson, B., Hansson, L., & Lexén, A. (2018). Outcomes of clients in need of intensive team care in Flexible Assertive Community Treatment in Sweden. *Nordic Journal of Psychiatry,**72*(3), 226–231. 10.1080/08039488.2018.1430168 29373933 10.1080/08039488.2018.1430168

[CR179] Sytema, S., Jörg, F., Nieboer, R., & Wunderink, L. (2014). Adding evidence-based interventions to assertive community treatment: A feasibility study. *Psychiatric Services,**65*(5), 689–692. 10.1176/appi.ps.201300143 24584988 10.1176/appi.ps.201300143

[CR178] Sytema, S., Wunderink, L., Bloemers, W., Roorda, L., & Wiersma, D. (2007). Assertive community treatment in the Netherlands: A randomized controlled trial. *Acta Psychiatrica Scandinavica,**116*(2), 105–112. 10.1111/j.1600-0447.2007.01021.x 17650271 10.1111/j.1600-0447.2007.01021.x

[CR180] Tang, F., Evans, C., Bogdan, A., Bullock, H., Westen, K., Kroon, H., & Delespaul, P. (2025). What is known about flexible assertive community treatment across populations and contexts? A scoping review protocol. *BMJ Open,**15*, Article e096100. 10.1136/bmjopen-2024-096100 41193201 10.1136/bmjopen-2024-096100 PMC12587939

[CR181] Tempier, R., Balbuena, L., Garety, P., & Craig, T. J. (2012). Does assertive community outreach improve social support? Results from the Lambeth Study of early-episode psychosis. *Psychiatric Services,**63*(3), 216–222. 10.1176/appi.ps.20110013 22388528 10.1176/appi.ps.20110013

[CR182] Test, M. A., & Stein, L. I. (1976). Practical guidelines for the community treatment of markedly impaired patients. *Community Mental Health Journal,**12*(2), 72–82. 10.1007/BF01435740 182432 10.1007/BF01435740

[CR183] Thoegersen, M. H., Morthorst, B. R., & Nordentoft, M. (2019). Assertive community treatment versus standard treatment for severely mentally ill patients in Denmark: A quasi-experimental trial. *Nordic Journal of Psychiatry,**73*(2), 149–158. 10.1080/08039488.2019.1576765 30894038 10.1080/08039488.2019.1576765

[CR184] Tholen, M. G., Martin, A., Stemeseder, T., Vikoler, T., Wageneder, B., Aichhorn, W., & Kaiser, A. K. (2024). Evaluation of a flexible assertive community treatment (FACT) program for patients with severe mental illness: An observational study in Salzburg, Austria. *International Journal of Mental Health Systems,**18*, Article 6. 10.1186/s13033-024-00628-8 38336693 10.1186/s13033-024-00628-8 PMC10858489

[CR185] Tibbo, P., Chue, P., & Wright, E. (1999). Hospital outcome measures following assertive community treatment in Edmonton, Alberta. *Canadian Journal of Psychiatry. Revue Canadienne De Psychiatrie,**44*(3), 276–279. 10.1177/070674379904400309 10225130 10.1177/070674379904400309

[CR186] Tibbo, P., Joffe, K., Chue, P., Metelitsa, A., & Wright, E. (2001). Global assessment of functioning following assertive community treatment in Edmonton, Alberta: A longitudinal study. *Canadian Journal of Psychiatry. Revue Canadienne De Psychiatrie,**46*(2), 144–148. 10.1177/070674370104600205 11280083 10.1177/070674370104600205

[CR187] Tinland, A., Loubière, S., Boucekine, M., Boyer, L., Fond, G., Girard, V., & Auquier, P. (2020). Effectiveness of a housing support team intervention with a recovery-oriented approach on hospital and emergency department use by homeless people with severe mental illness: A randomised controlled trial. *Epidemiology and Psychiatric Sciences,**29*, Article e169. 10.1017/S2045796020000785 32996442 10.1017/S2045796020000785 PMC7576524

[CR188] Tomar, N., Ghezzi, M., Wilson, A., Deinse, T., Burgin, S., & Cuddeback, G. (2020). Demographic and clinical characteristics of consumers who transition from assertive community treatment to less intensive services. *Social Work in Mental Health,**18*(6), 665–676. 10.1080/15332985.2020.1769002

[CR189] Tranberg, K., Colnadar, B., Nielsen, M. H., Hjorthøj, C., & Møller, A. (2024). Interventions targeting patients with co-occurring severe mental illness and substance use (dual diagnosis) in general practice settings: A scoping review of the literature. *BMC Primary Care,**25*(1), Article 281. 10.1186/s12875-024-02504-3 39097682 10.1186/s12875-024-02504-3 PMC11297724

[CR190] Tricco, A. C., Lillie, E., Zarin, W., O’Brien, K. K., Colquhoun, H., Levac, D., Moher, D., Peters, M. D. J., Horsley, T., Weeks, L., Hempel, S., Akl, E. A., Chang, C., McGowan, J., Stewart, L., Hartling, L., Aldcroft, A., Wilson, M. G., Garritty, C., & Straus, S. E. (2018). PRISMA extension for scoping reviews (PRISMA-ScR): Checklist and explanation. *Annals of Internal Medicine,**169*(7), 467–473. 10.7326/M18-0850 30178033 10.7326/M18-0850

[CR191] Udechuku, A., Olver, J., Hallam, K., Blyth, F., Leslie, M., Nasso, M., Schlesinger, P., Warren, L., Turner, M., & Burrows, G. (2005). Assertive community treatment of the mentally ill: Service model and effectiveness. *Australasian psychiatry : bulletin of Royal Australian and New Zealand College of Psychiatrists,**13*(2), 129–134. 10.1080/j.1440-1665.2005.02175.x 15948908 10.1080/j.1440-1665.2005.02175.x

[CR192] Valenstein, M., McCarthy, J. F., Ganoczy, D., Bowersox, N. W., Dixon, L. B., Miller, R., Visnic, S., & Slade, E. P. (2013). Assertive community treatment in veterans affairs settings: Impact on adherence to antipsychotic medication. *Psychiatric Services (Washington, D.C.),**64*(5), 445-451.e1. 10.1176/appi.ps.201100543 23412131 10.1176/appi.ps.201100543

[CR196] Vanderlip, E. R., Henwood, B. F., Hrouda, D. R., Meyer, P. S., Monroe-DeVita, M., Studer, L. M., Schweikhard, A. J., & Moser, L. L. (2017). Systematic literature review of general health care interventions within programs of assertive community treatment. *Psychiatric Services,**68*(3), 218–224. 10.1176/appi.ps.201600100 27903142 10.1176/appi.ps.201600100

[CR216] van Veldhuizen, J. R. (2007). FACT: a Dutch version of ACT. *Community mental health journal*, *43*(4), 421–433. 10.1007/s10597-007-9089-4 17514502 10.1007/s10597-007-9089-4

[CR193] van Vugt, M. D., Kroon, H., Delespaul, P. A., Dreef, F. G., Nugter, A., Roosenschoon, B. J., van Weeghel, J., Zoeteman, J. B., & Mulder, C. L. (2011). Assertive community treatment in the Netherlands: Outcome and model fidelity. *Canadian Journal of Psychiatry. Revue Canadienne De Psychiatrie,**56*(3), 154–160. 10.1177/070674371105600305 21443822 10.1177/070674371105600305

[CR194] van Vugt, M. D., Kroon, H., Delespaul, P. A., & Mulder, C. L. (2012). Consumer-providers in assertive community treatment programs: Associations with client outcomes. *Psychiatric Services (Washington, D.C.),**63*(5), 477–481. 10.1176/appi.ps.201000549 22388475 10.1176/appi.ps.201000549

[CR195] van Vugt, M. D., Kroon, H., Delespaul, P. A., & Mulder, C. L. (2014). Assertive community treatment and associations with substance abuse problems. *Community Mental Health Journal,**50*(4), 460–465. 10.1007/s10597-013-9626-2 23771775 10.1007/s10597-013-9626-2

[CR197] Vidal, S., Perroud, N., Correa, L., & Huguelet, P. (2020). Assertive community programs for patients with severe mental disorders: Are benefits sustained after discharge? *Community Mental Health Journal,**56*(3), 559–567. 10.1007/s10597-019-00513-6 31807993 10.1007/s10597-019-00513-6

[CR198] Vijverberg, R., Ferdinand, R., Beekman, A., & Meijel, B. (2017). The effect of youth assertive community treatment: A systematic PRISMA review. *BMC Psychiatry,**17*(1), Article 284. 10.1186/s12888-017-1446-4 28768492 10.1186/s12888-017-1446-4 PMC5541424

[CR199] Wasmer, D., Pinkerton, M., Dincin, J., & Rychlik, K. (1999). Impact of flexible duration assertive community treatment: Program utilization patterns and state hospital use. *The Journal of Rehabilitation*, *13*(6), 25–30.

[CR200] Waynor, W. R., Eissenstat, S. J., Yanos, P. T., Reinhardt-Wood, D., Taylor, E., Karyczak, S., & Lu, W. (2020). The role of illness identity in assertive community treatment. *Rehabilitation Counseling Bulletin,**63*(4), 216–223. 10.1177/0034355219886916

[CR201] Westen, K., Boyle, P., & Kroon, H. (2022). An observational comparison of FACT and ACT in the Netherlands and the US. *BMC Psychiatry,**22*, Article 311. 10.1186/s12888-022-03927-x 35505332 10.1186/s12888-022-03927-x PMC9063161

[CR202] Westen, K. H., Peeters, P., Delespaul, P., & Kroon, H. (2025). Heeft het FACT-model toekomst? [Does FACT have a future?]. *Tijdschrift Voor Psychiatrie,**67*(10), 608–611. 41466588

[CR203] Wilk, P., Vingilis, E., Bishop, J. E., He, W., Braun, J., Forchuk, C., Seeley, J., & Mitchell, B. (2013). Distinctive trajectory groups of mental health functioning among assertive community treatment clients: An application of growth mixture modelling analysis. *Canadian Journal of Psychiatry. Revue Canadienne De Psychiatrie,**58*(12), 670–678. 10.1177/070674371305801204 24331286 10.1177/070674371305801204

[CR204] Williamson, T. (2002). Ethics of assertive outreach (assertive community treatment teams). *Current Opinion in Psychiatry,**15*(5), 543–547. 10.1097/00001504-200209000-00009 15264342 10.1097/00001504-200209000-00013

[CR205] Wolff, N., Helminiak, T. W., Morse, G. A., Calsyn, R. J., Klinkenberg, W. D., & Trusty, M. L. (1997). Cost-effectiveness evaluation of three approaches to case management for homeless mentally ill clients. *American Journal of Psychiatry,**154*(3), 341–348. 10.1176/ajp.154.3.341 9054781 10.1176/ajp.154.3.341

[CR206] Wood, K., & Anderson, J. (1995). The effect on hospital admissions of psychiatric case management involving general practitioners: Preliminary results. *Australian and New Zealand Journal of Psychiatry,**29*(2), 223–229. 10.1080/00048679509075914 7487784 10.1080/00048679509075914

[CR209] World Health Organization (1992). International classification of diseases: Tenth revision (ICD-10). World Health Organization. https://icd.who.int/browse10/2019/en

[CR210] World Health Organization (2019). International classification of diseases (11th ed). *World Health Organization*. https://icd.who.int/browse11/l-m/en

[CR211] World Health Organization (2021). Guidance on community mental health services: Promoting person-centred and rights-based approaches. *World Health Organization*. https://www.who.int/publications/i/item/9789240025707

[CR212] World Health Organization (2022a). Mental disorders. World Health Organization. https://www.who.int/news-room/fact-sheets/detail/mental-disorders

[CR207] World Health Organization (2022b). Mental health: Strengthening our response. World Health Organization. https://www.who.int/es/news-room/fact-sheets/detail/mental-health-strengthening-our-response

[CR208] World Health Organization (2022c). World mental health report: Transforming mental health for all. World Health Organization. https://iris.who.int/bitstream/handle/10665/356118/9789240051966-eng.pdf

[CR213] Zavradashvili, N., Donisi, V., Grigoletti, L., Pertile, R., Gelashvili, K., Eliashvili, M., & Amaddeo, F. (2010). Is the implementation of assertive community treatment in a low-income country feasible? The experience of Tbilisi, Georgia. *Social Psychiatry and Psychiatric Epidemiology,**45*(8), 779–783. 10.1007/s00127-009-0125-2 19710993 10.1007/s00127-009-0125-2

[CR214] Zhao, W., Law, S., Luo, X., Chow, W., Zhang, J., Zhu, Y., Liu, S., Ma, X., Yao, S., & Wang, X. (2015). First adaptation of a family-based ACT model in mainland China: A pilot project. *Psychiatric Services,**66*(4), 438–441. 10.1176/appi.ps.201400087 25555033 10.1176/appi.ps.201400087

[CR215] Zumstein, N., & Riese, F. (2020). Defining severe and persistent mental illness: A pragmatic utility concept analysis. *Frontiers in Psychiatry,**11*, Article 648. 10.3389/fpsyt.2020.00648 32733295 10.3389/fpsyt.2020.00648 PMC7358610

