# Supplementary material for: Assertive Community Treatment and ACT-Derived Outreach Models: A Scoping Review of Adaptations, Fidelity and Outcomes
Source: Community Ment Health J. 2026 Mar 26;62(6):1210–29. doi: 10.1007/s10597-026-01607-8 (PMC13451395; doi:10.1007/s10597-026-01607-8)
Supplement: Supplementary file 1 [file 10597_2026_1607_MOESM1_ESM.docx]

**Supplementary material**

**Table S1.** Search strategy and results obtained across databases.

| **Database** | **Search Strategy** | **Results** | **Search Date** |
| --- | --- | --- | --- |
| **Medline (PubMed)** | ("Assertive community treatment" OR "Community Treatment, Assertive" OR "Treatment, Assertive Community") AND ("Mental disorders" OR "Mentally Ill Persons" OR Schizophrenia OR "Bipolar disorder" OR "Psychotic Disorders" OR "Depressive Disorder, Major" OR "Obsessive-Compulsive Disorder" OR "Personality Disorders") AND ("Treatment Adherence and Compliance" OR "Community Integration" OR "Quality of Life" OR "Psychic Symptoms" OR "Patient Readmission" OR Hospitalization OR Emergencies OR Effectiveness) | 583 | 05/10/2024 |
| **Web of Science (WoS)** | ("Assertive community treatment" OR "Community Treatment, Assertive" OR "Treatment, Assertive Community") AND ("Mental disorders" OR "Mentally Ill Persons" OR Schizophrenia OR "Bipolar disorder" OR "Psychotic Disorders" OR "Depressive Disorder, Major" OR "Obsessive-Compulsive Disorder" OR "Personality Disorders") AND ("Treatment Adherence and Compliance" OR "Community Integration" OR "Quality of Life" OR "Psychic Symptoms" OR "Patient Readmission" OR Hospitalization OR Emergencies OR Effectiveness) | 691 | 05/11/2024 |
| **Cochrane Library** | ("Assertive community treatment" OR "Community Treatment, Assertive" OR "Treatment, Assertive Community") AND ("Mental disorders" OR "Mentally Ill Persons" OR Schizophrenia OR "Bipolar disorder" OR "Psychotic Disorders" OR "Depressive Disorder, Major" OR "Obsessive-Compulsive Disorder" OR "Personality Disorders") AND ("Treatment Adherence and Compliance" OR "Community Integration" OR "Quality of Life" OR "Psychic Symptoms" OR "Patient Readmission" OR Hospitalization OR Emergencies OR Effectiveness) | 155 | 05/10/2024 |
| **Scopus** | ("Assertive community treatment" OR "Community Treatment, Assertive" OR "Treatment, Assertive Community") AND ("Mental disorders" OR "Mentally Ill Persons" OR Schizophrenia OR "Bipolar disorder" OR "Psychotic Disorders" OR "Depressive Disorder, Major" OR "Obsessive-Compulsive Disorder" OR "Personality Disorders") AND ("Treatment Adherence and Compliance" OR "Community Integration" OR "Quality of Life" OR "Psychic Symptoms" OR "Patient Readmission" OR Hospitalization OR Emergencies OR Effectiveness) | 439 | 05/11/2024 |
| **CINAHL** | ("Assertive community treatment" OR "Community Treatment, Assertive" OR "Treatment, Assertive Community") AND ("Mental disorders" OR "Mentally Ill Persons" OR Schizophrenia OR "Bipolar disorder" OR "Psychotic Disorders" OR "Depressive Disorder, Major" OR "Obsessive-Compulsive Disorder" OR "Personality Disorders") AND ("Treatment Adherence and Compliance" OR "Community Integration" OR "Quality of Life" OR "Psychic Symptoms" OR "Patient Readmission" OR Hospitalization OR Emergencies OR Effectiveness) | 739 | 05/12/2024 |
| **PsycInfo** | ("Assertive community treatment" OR "Community Treatment, Assertive" OR "Treatment, Assertive Community") AND ("Mental disorders" OR "Mentally Ill Persons" OR Schizophrenia OR "Bipolar disorder" OR "Psychotic Disorders" OR "Depressive Disorder, Major" OR "Obsessive-Compulsive Disorder" OR "Personality Disorders") AND ("Treatment Adherence and Compliance" OR "Community Integration" OR "Quality of Life" OR "Psychic Symptoms" OR "Patient Readmission" OR Hospitalization OR Emergencies OR Effectiveness) | 1,283 | 05/12/2024 |
| **ProQuest** | ("Assertive community treatment" OR "Community Treatment, Assertive" OR "Treatment, Assertive Community") AND ("Mental disorders" OR "Mentally Ill Persons" OR Schizophrenia OR "Bipolar disorder" OR "Psychotic Disorders") AND ("Treatment Adherence and Compliance" OR "Community Integration" OR "Quality of Life" OR "Psychic Symptoms" OR "Patient Readmission" OR Hospitalization OR Emergencies OR Effectiveness) | 4,945 | 01/27/2024 |
| **IBECS** | “Tratamiento asertivo comunitario” | 11 | 01/26/2024 |
| **LILACS** | “Tratamiento asertivo comunitario” | 317 | 01/26/2024 |

Table developed by the authors.

| **Table S2:** **General characteristics of included studies.** | | | | | | | |
| --- | --- | --- | --- | --- | --- | --- | --- |
| **Reference; Year; Country** | **Study type/**  **Program (ACT-model classification)** | **Participants (N, medical diagnosis, age and sex)  Groups (if applicable)** | **Inclusion criteria** | **Residential setting** | **Data collection dates** | **Measures/outcomes** | **Main results** |
| Aagaard et al. (2011); Denmark | Quasi-experimental study with non-randomized control group.  Classic ACT (Stein & Test–based ACT team), rural Denmark; high-fidelity ACT implementation (McGrew 27/31; 87% | N = 174 Medical diagnosis: SMI Age: Mean = 41 years Sex: 58% men  Groups:   - N interention group (ACT) = 86 - N control group = 88 | - ICD-10 diagnosis: F20, F22, F25, or F31 - And at least one of the following:  a) Drug or alcohol abuse (F10–F19) or forensic sanction (Z04.6) b) Intensive hospital use: ≥4 admissions or ≥50 days of inpatient or day hospitalization in the past 2 years | Rural | Recruitment and intervention: from May 2001. Follow-up: 2 years after entry into ACT or usual care | - Hospitalization: admissions, bed days, hospital days. - CANSAS (Camberwell Assessment of Needs) - SAPS (positive symptoms) - SANS (negative symptoms) - CSQ (Client Satisfaction Questionnaire) | Significant reduction in hospitalization, greater engagement with the system, improved functioning, and higher patient satisfaction were observed. No long-term improvements were found in psychotic symptoms. The value of an integrated approach is emphasized, including resources such as group therapy, vocational support, and crisis intervention. |
| Aagaard et al. (2016); Denmark | Observational. Retrospective cohort study.  Classic ACT (standard ACT teams in North Denmark Region); fidelity assessed with McGrew (variable fidelity across teams: 90.3%, 77.4%, 48.4%) | N = 240 Medical diagnosis: SMI Age: Mean = 41 years Sex: 52.5% men  Groups:   - N ACT Team North group = 86 - N ACT Team South group = 106 - N ACT Team Thy/Mors group = 48 | - Primary diagnosis of F20, F22, F25 or F31 - And at least one of the following:  a) Substance use disorder (F10–F19) or forensic sanction (Z04.6) b) High use of psychiatric hospitals ≥4 admissions or ≥50 hospital days in the past 2 years | Rural | January 2008 – January 2011. Follow-up: 2 years before and 2 years after inclusion | - Psychiatric hospitalizations (number and days). - Psychiatric emergency room visits - Psychiatric outpatient visits - Use of somatic hospitals - Pharmacological treatment (before and after) | A significant reduction was observed in the number of admissions, hospital days, and emergency visits. In contrast, outpatient consultations increased substantially. No notable changes were found in the use of hospitals for organic medical conditions. Pharmacological treatment showed a general decrease in the use of psychotropic medications. |
| Aagaard et al. (2017); Denmark | Observational. Retrospective cohort study.  Classic ACT (intensive case management / ACT program), North Denmark Region; ACT implemented as regional ACT teams (caseload ~1:10; 24-h crisis support; MDT approach) | N= 240  Medical diagnosis: SMI  Age: Mean: 41  Sex: 52.5% men   Groups:  N/A | - Primary diagnosis of F20, F22, F25 or F31 - And at least one of the following:  a) Substance use disorder (F10–F19) or forensic sanction (Z04.6) b) High use of psychiatric hospitals ≥4 admissions or ≥50 hospital days in the past 2 years  - Additional selection based on non-compliance or insufficient outpatient treatment. | Mixed | 1995/2008-2012 | Use of psychiatric service:   - Admissions (voluntary and involuntary) - Dangerous criterion - Treatment criterion - Bed days - Consultations, Psychiatric emergency visits (PER) | A significant reduction was observed in the number of voluntary admissions, bed days, and psychiatric emergency visits. Although not statistically significant, there was a downward trend in admissions based on risk criteria. No changes were found in admissions based on treatment needs or forensic criteria. Overall, ACT is considered preferable from both the patient’s and the care team’s perspectives. |
| Ahrens et al. (2007); USA | Uncontrolled prospective pre-post study.  Program: PACT (classic ACT/PACT**)**; conventional PACT services + youth-specific enhancements (supported education, family/foster coordination, group therapy/recreation); no program structural modifications. | N = 15 Medical diagnosis: Schizophrenia (20%), Schizoaffective disorder (67%), Bipolar disorder Age: Mean = 16.8 years, SD = 1.4 Sex: 80% male  Groups: N/A | - Aged 15 - 21 years. - Diagnosis of schizophrenia, schizoaffective disorder, or bipolar disorder. - Four or more functional limitations, including: history of frequent hospitalizations, juvenile institutionalization, school truancy, homelessness, or history of foster care placement. | Urban | April 1998 - May 2000. 12-month follow-up | - Days of psychiatric hospitalization in the year before and after PACT - Days in forensic treatment or incarceration - Total days in institutions (psychiatric hospitalization + forensic treatment/incarceration) - Frequency of hospital admissions | PACT led to a significant reduction in psychiatric hospitalization and overall institutionalization, including incarceration. However, no significant change was observed in the frequency of hospital admissions. These findings are consistent with previous studies on PACT in adult populations. |
| Albert et al. (2017); Denmark | RCT  Program: OPUS (SEI); includes modified ACT, family involvement, and social skills training (case manager ratio ≤ 12:1; extended OPUS up to 5 years in intervention arm). | N = 400 Medical diagnosis: Schizophrenia spectrum disorder (ICD-10) Age: OPUS group mean = 25.6 years, SD = 4.3 Sex: OPUS group = 53.8% male; TAU group = 43.3% male  Groups:   - N intervention group (OPUS) = 197 - N control group (TAU) = 203 | - Aged 18 - 35 years. - Diagnosis of schizophrenia spectrum disorder according to ICD-10. - No intellectual disability (IQ < 70), as such patients were not treated within OPUS. | Urban | 2009 – 2012. Five-year follow-up (until 2015) | - Negative symptoms: SANS (Scale for the Assessment of Negative Symptoms) - Symptom remission: SAPS + SANS - Psychotic symptoms: SAPS (Scale for the Assessment of Positive Symptoms) - Substance abuse: SCAN - Patient satisfaction: CSQ (Client Satisfaction Questionnaire) - Treatment adherence: Records (service contact) - Medication adherence: Self-report (percentage of doses taken) - Suicidality: Self-report - Days of hospitalization: National Register - Employment participation: DREAM Register - Symptom remission at 2 years: Life Chart Schedule - Disorganized dimension: SAPS and SANS - Global functioning: GAF and PSP - Cognitive functioning: BACS - Therapeutic alliance: Working Alliance Inventory - Self-efficacy: General Self-Efficacy Scale - Quality of life: WHOQOL-BREF - Outpatient contacts - Psychiatric emergency contacts | The experimental group had greater contact with mental health services and reported higher levels of patient satisfaction. A stronger therapeutic alliance was also observed in the experimental group. However, there were no differences between groups in hospitalization rates, employment status, or remission of psychotic symptoms. Some individuals from the control group eventually received ACT, which may have reduced the differences observed between groups. |
| Arahanthabailu et al. (2022); India | Observational. Retrospective pre–post study with cross-sectional follow-up.  Modified ACT (M-ACT / M-CAT); outreach/home visits (monthly MDT visit + phone follow-up every 2 weeks; 24-h crisis phone support as needed); low caseload (4–5 clients per case coordinator; max 35); fidelity assessed with DACTS (2.9 = low fidelity). | N = 10 Medical diagnosis: Schizophrenia Age: Mean = 42 years, SD = 11.1 Sex: 60% men  Groups: N/A | Individuals with chronic schizophrenia, multiple relapses, low adherence to treatment, and poor family support. | Predominantly rural (7 rural, 3 urban) | N.D. | - Number of hospitalizations - Adherence to treatment - SOFAS (social and occupational functioning). - IDEAS (mental health disability) - Service perception questionnaire (patients and carers). | A significant reduction in hospitalizations was observed, along with increased treatment adherence and improved overall functioning. There was also a noticeable decrease in disability levels. All patients and caregivers expressed satisfaction with the program and indicated they would recommend the service. |
| Aubry et al. (2015); Canada | RCT  Housing First (Pathways model) with ACT support (HF+ACT) | N = 950 Medical diagnosis: Mental disorder Age: Mean = 39.4 years, SD = 11.03 Sex: 68% male  Groups:   - N Intervention group (HF) = 469 - N Control group (TAU) = 481 | - Legal adult age (≥18 years; ≥19 in Vancouver). - Homeless or unstably housed. - Severe mental disorder (according to MINI 6.0). - Not receiving ACT at study entry. | Urban | October 2009 – July 2011. Follow-up: 12 months after group assignment (until June 2012) | - Housing stability: Measured with the Residential Time-Line Follow-Back Inventory. Defined as living in a stable place ≥6 months with tenancy rights - Quality of life: 20-item Quality of Life Interview (QOLI-20) - Psychiatric symptoms: Colorado Symptom Index (CSI) - Substance use: Global Appraisal of Individual Needs – Short Screener (GAIN-SS). Problem use defined as ≥2 symptoms in the past month - Community functioning: Multnomah Community Ability Scale (MCAS) | Housing First resulted in greater housing stability compared to usual treatment. Participants in the Housing First group also reported a higher quality of life, with improvements in housing conditions, sense of safety, and opportunities for leisure. Additionally, they showed better functioning within the community. No significant differences were observed between the groups in terms of psychiatric symptoms or substance use. |
| Aubry et al. (2016); Canada | RCT  Housing First (Pathways model) with ACT support (HF+ACT) | N = 950 Medical diagnosis: Mental disorder Age: Mean = 39.4 years, SD = 11.03 Sex: 68% male  Groups:   - N intervention group (HF) = 469 - N control group (TAU) = 481 | - Aged ≥18 years (≥19 in Vancouver) - Absolute homelessness or precarious housing situation. - Current diagnosis of a mental disorder according to MINI 6.0 or recent written diagnosis. - Not currently receiving ACT or ICM. - Legal status as a Canadian citizen, permanent resident, or asylum seeker. | Urban | October 2009 – August 2011, with last follow-up in June 2013 | - Time to first stable housing - Proportion of time in stable housing - Consecutive days housed at endpoint - Perceived housing quality - Community functioning: MCAS - Quality of life: QoLI-20 - Self-reported health status: EQ-5D - Mental health symptoms: CSI - Substance use problems: GAIN-SPS - Hospital days, emergency visits, and arrests | Housing First demonstrated greater housing stability, quicker access to stable housing, and longer housing retention, along with better housing quality. Initial gains were observed in quality of life and community functioning, although these differences diminished by the second year. No significant differences were found between groups in mental health or substance use, although the control group showed a slight advantage in mental health outcomes at the end of the study. Both groups showed a reduction in service utilization, including hospitalizations, emergency visits, and arrests. Housing First was also more cost-effective, resulting in substantial annual savings per participant. |
| Aubry et al. (2019); Canada | RCT  Housing First (Pathways model) with ACT support (HF+ACT) — small-city implementation | N = 201 Medical diagnosis: SMI Age:   - HF group: 18–30 = 29%, 31–40 = 21%, 41–50 = 31%, >51 = 20% - TAU group: 18–30 = 22%, 31–40 = 27%, 41–50 = 37%, >51 = 14%   Sex:   - HF group = 66% male - TAU group = 63% male   Groups:   - N intervention group (HF) = 100 - N control group (TAU) = 101 | - Aged ≥18. - Absolute or precarious homelessness. - SMI (using the Mini International Neuropsychiatric Interview – MINI). - Canadian citizenship, permanent residence, or asylum seeker status. - Not receiving ACT (Assertive Community Treatment) or ICM (Intensive Case Management) at study entry. | Urban | October 2009 – February 2013 | - Housing stability (days in stable housing, perceived quality, number of relocations) - Community functioning (Multnomah Community Ability Scale – MCAS) - Self-reported health status (EQ-5D) - Mental health symptoms (Colorado Symptom Index – CSI) - Substance use issues (GAIN-SPS) - Quality of life (QoLI-20) - Perceived recovery (Recovery Assessment Scale – RAS) | Participants in the Housing First group reported significantly greater improvements in quality of life compared to those receiving treatment as usual. Housing First also achieved higher rates of housing stability. Although the number of psychiatric hospitalizations was lower in the Housing First group, the total number of hospital days was higher. Improvements were noted in social and psychological integration, including perceived safety, financial stability, and leisure activities. Both groups showed progress in mental health symptoms and substance use issues, with no significant differences between them |
| Baier et al. (2013); Switzerland | Quasi-experimental pre-post study without a control group.  ACT adaptation for adolescents (early tailored assertive community case management / mobile outreach team) — AIMA program (Antenne d’Intervention dans le Milieu pour Adolescents), Lausanne, Switzerland | N = 35 Medical diagnosis: Psychiatric disorders   - Psychotic disorders = 51.43% - Mood disorders = 14.29% - Anxiety disorders = 8.57% - Conduct disorders = 25.71%   Age: Mean = 16.3 years, SD = 1.4 Sex: 42.86% men  Groups: N/A | Adolescents aged 13–18 years with psychiatric disorders who refuse treatment in conventional outpatient services. | Urban | 2009-2010 | - Escala HoNOSCA (Health of the Nation Outcome Scales for Children and Adolescents) | Significant improvements were observed in areas such as hyperactivity and concentration, academic and language skills, non-organic somatic symptoms, emotional symptoms, peer and family relationships, school attendance, and awareness of personal difficulties. Overall functioning, as measured by the total HoNOSCA score, also showed substantial improvement. However, no significant improvements were found in substance abuse. |
| Bertelsen et al. (2008); Denmark | RCT, 5-year follow-up  OPUS (Specialized Early Intervention, SEI) — integrated early-intervention program including ACT + family involvement + social skills training. | N = 547 Medical diagnosis: First episode of psychotic illness Age:   - Integrated treatment group: Mean = 26.6 years, SD = 6.4 - Standard treatment group: Mean = 26.6 years, SD = 6.3.   Sex:   - Integrated treatment group: 58% male - Standard treatment group: 60% male   Groups:   - N Intervention group (Integrated treatment) = 275 - N Control group (Standard treatment) = 272 | - Aged 18 - 45 years. - First contact with mental health services with a diagnosis within the schizophrenia spectrum (ICD-10). - No more than 12 continuous weeks of antipsychotic medication prior to study entry. | Urban | January 1998 – December 2000. Follow-up at 2 and 5 years | - Psychotic and negative symptoms: Scale for the Assessment of Positive Symptoms (SAPS) and Scale for the Assessment of Negative Symptoms (SANS) - Global functioning: Global Assessment of Functioning (GAF) - Substance use: Substance abuse diagnosis (ICD-10) - Presence of depression - Suicidal behavior (ideation and attempts) - Number of hospitalizations - Total hospital days - Number of outpatient consultations - Use of psychiatric emergency services - Housing type (independent or supervised) - Days in supervised housing - Employment or education | The intensive intervention led to improvements in symptoms and quality of life during the first two years; however, these effects were not sustained at the five-year follow-up. There was a lasting benefit in terms of reduced institutionalization and lower reliance on intensive psychiatric services. These findings suggest that longer-term interventions may be necessary to maintain positive outcomes over time. |
| Bodén et al. (2010); Sweden | Observational. Historical cohort study.  Modified ACT (mACT) — implementation of modified ACT teams for first-episode non-affective psychosis in Uppsala County (Sweden), compared with pre-implementation treatment-as-usual (non-mACT). | N = 144 Medical diagnosis: Non-affective psychosis (first episode) Age:   - Non-mACT median = 26, IQR = 12; - mACT median = 26, IQR = 12   Sex:   - Non-mACT = 52% men - mACT = 67% men   Groups:   - N Non-mACT group = 66 - N mACT group = 78 | - First episode of psychosis - Aged 16-59 years - DSM-IV diagnosis of non-affective psychosis - Exclusion: organic cause or severe substance abuse | Mixed | 1995–2000 (cohort identification) 5-year follow-up | - PANSS (positive and negative symptoms) - GAF - AUDIT - Subjective Satisfaction With Life (SSWL) - Days of hospitalization - Number of admissions - Employment - Education - Independent living - Suicide - Substance use | At five-year follow-up, the mACT group showed no significant clinical improvement compared to the non-mACT group, with a trend toward worse positive symptoms and higher use of second-generation antipsychotics. The modified ACT program did not demonstrate long-term benefits. Further research is needed to determine the optimal duration and effective components of inpatient and community-based care before reducing inpatient services. |
| Bond et al. (1990); USA | RCT  ACT / PACT (large-city adaptation of ACT model) — Urban ACT team delivering assertive outreach and in vivo services, based on Stein & Test model. | N = 88  Medical diagnosis: SMI Age:   - ACT group: Mean = 33 years - Drop-in center group: Mean = 35.6 years   Sex:   - ACT group: 53% male - Drop-in center group: 60% male   Groups:   - N Intervention group (PACT) = 45 - N Control group (Drop-in center) = 43 | - Aged ≥18 years. - Diagnosis of schizophrenia, schizoaffective disorder, affective disorder, or severe personality disorder. - History of frequent psychiatric hospitalization: - At least 3 hospitalizations in the past 2 years - At least 5 hospitalizations over the lifetime. - Residence within a 3-mile radius of the treatment program. - No prior significant contact with the ACT or DI program evaluated in the study. | Urban | July 1984 – July 1986. Follow-up: 12 months after admission | - Psychiatric hospital use during the 12 months after treatment allocation - Number of psychiatric hospitalizations - Total number of days hospitalized - Frequency of police contacts - Residential stability (community housing after 12 months) - Client Satisfaction Questionnaire (CSQ) - Quality of life: Life Satisfaction Checklist (LSC) - Daily living difficulties: Areas of Difficulty Checklist (ADC) | Patients in the ACT group were hospitalized for significantly fewer days compared to those in the DI group. ACT also led to a reduction in psychiatric readmissions and was associated with higher satisfaction with services. There was less police contact among ACT participants and greater housing stability. Additionally, the ACT program was more cost-effective, with lower costs per patient than the DI approach. |
| Bond et al. (1991); USA | Quasi-experimental controlled.  Classic ACT (Stein & Test–based ACT; CMHC demonstration projects modeled after the Bridge program) — ACT for young adults with severe mental illness and substance abuse (MISA) | N = 97 Medical diagnosis: SMI Age: Mean = 31.5 years Sex: 79% men  Groups:   - N ACT group = 31 - N RG group = 23 - N control group = 43 | - Aged 18 - 45 years. - Diagnosis of SMI as defined by the Indiana Department of Mental Health. - Diagnosis of substance abuse or dependence, or documented evidence of use. - Frequent use of hospitals or psychiatric crisis services in the past year (≥2 hospitalizations, emergency room visits, jail stays, or shelters). | Urban | 1897 - 1991.- 18-month follow-up | - Program retention - Number of contacts with healthcare professionals - Number of hospital admissions - Number of hospitalization days - Substance use (DAPS and MAST) - Perceived quality of life (Life Satisfaction Checklist – LSC) - Frequency of arrests and incarcerations - Housing situation: whether the patient has secured stable accommodation - Time in employment: proportion of participants who obtain and maintain a job - Patient satisfaction | Higher treatment adherence was observed in both the ACT and RG groups compared to the control group. Hospitalizations decreased in the ACT and control groups, while no such change was seen in the RG group. The RG group demonstrated greater reductions in alcohol and marijuana use. Both ACT and RG participants reported a better quality of life than those in the control group. |
| Bond et al. (2004); USA | Observational. Retrospective pre–post study.  Classic ACT (DACTS-defined ACT; statewide ACT implementation initiative) with fidelity monitoring using the Dartmouth ACT Scale (DACTS) | N = 317 Medical diagnosis: SMI   - Schizophrenia spectrum disorder: 70.5% - Affective disorder: 16.4% - Other psychiatric disorder: 13.1%   Age: Mean = 37.8 years, SD = 9.4 Sex: 65.4% men  Groups: N/A | - Frequent users of state psychiatric hospitals (≥3 admissions in 1 year, ≥5 in 2 years or ≥180 days hospitalized in the previous year) - Admission to state-funded ACT teams - Participation for ≥12 months - Consent given | Urban | 1994–1996 | - Reduction in days of hospitalization in state hospitals (pre-post 12 months) - Fidelity to the ACT model (DACTS, 25 items, score range 1–5) | A substantial reduction in average hospital stay was observed following the intervention. Most high-fidelity ACT teams achieved better outcomes, and all sites that showed marked decreases in hospitalization had above-average fidelity scores. |
| Bonsack et al. (2005); Switzerland | Observational. Retrospective cohort study.  Time-limited ACT / Assertive Outreach (ACT-derived adaptation) — intensive, home-based assertive outreach program in Lausanne (Switzerland) targeting difficult-to-engage patients and designed to facilitate linkage to outpatient care | N = 75 Medical diagnosis: SMI   - Psychosis: 68% - Affective disorder: 21% - Personality disorder: 7%   Age: Mean = 35.5 years (range 18–65) Sex: 55% men  Groups: N/A | - Aged 18–65 years - Resident of the catchment area (Lausanne and surroundings, 240,000 inhabitants) - At least one of the following: 3 psychiatric admissions in the last 12 months or >6 in 5 years; repeated missed outpatient appointments after discharge; persistent nonadherence or refusal of pharmacological treatment | Urban | January 2000 - March 2002 | - HoNOS (severe clinical and social problems ≥3) - MCAS (community skills) - CTRS (clinical urgency) - Motivation for treatment (Prochaska and DiClemente's model) - Social network support (5 qualitative levels) - Alcohol and drug use (5-point scales) | The intervention led to significant improvements in most clinical and social outcomes, including reductions in psychotic symptoms, substance use, and aggressive behavior, along with increased motivation, adherence, and social support. Treatment collaboration and medication adherence both improved substantially. Hospitalizations became less frequent and shorter in duration, especially among individuals with high prior use. Most participants completed the program within a relatively short period, while those requiring more time tended to have more complex needs and lower initial motivation. |
| Botha et al. (2010); South Africa | RCT  Modified ACT (mACT) — randomized controlled trial of a modified assertive community treatment intervention in an under-resourced/developing country context (South Africa). | N = 60 Medical diagnosis: Schizophrenia or schizoaffective disorder Age:   - ACT group = 30.55 years, SD = 9.09 - Control group = 34.81 years, SD = 11.02   Sex:   - ACT group = 74.19% male - Control group = 71.43% male   Groups:   - N intervention group (ACT) = 34 - N control group = 26 | - Confirmed diagnosis of schizophrenia or schizoaffective disorder. - Frequent users of psychiatric services (according to modified Weiden criteria). - Capacity to provide written informed consent. | Urban | 2007 – 2008 | - Positive and Negative Syndrome Scale (PANSS) - Calgary Depression Scale for Schizophrenia (CDSS) - Extrapyramidal Symptom Rating Scale (ESRS) - Social and Occupational Functioning Assessment Scale (SOFAS) - Quality of life: WHO-QOL - Number of readmissions - Days of psychiatric hospitalization: Clinical records - Illness remission: Andreasen criteria | Modified ACT led to reductions in hospitalizations and inpatient days. It also improved psychiatric symptoms and social functioning. Treatment adherence was higher in the modified ACT group. Notably, these positive outcomes were achieved using fewer resources than those typically required in models from high-income countries. |
| Botha et al. (2014); South Africa | RCT  Modified ACT (mACT) — 3-year follow-up RCT evaluating sustainability of a modified assertive intervention based on the international ACT model (South Africa) | N = 56 Medical diagnosis: Schizophrenia or schizoaffective disorder Age:   - Cases group mean = 32.0 years (IQR 26.8–42.8) - Control group mean = 27.5 years (IQR 23.8–36.8)   Sex:   - Cases = 69% male - Controls = 79% male   Groups:   - N intervention group = 32 - N control group = 24 | - Diagnosis of schizophrenia or schizoaffective disorder. - Need for antipsychotic treatment. - Meeting criteria for high-frequency hospitalization: - ≥3 admissions within 18 months, or - ≥5 admissions within 36 months, or - ≥2 admissions within 12 months with clozapine treatment, or - ≥2 admissions within 12 months and ≥120 days of hospitalization. | Mixed | Not specified. Follow-up: 36 months. Data collected over 72 months: 36 months pre-intervention (pre-DOI) and 36 months post-intervention (post-DOI) | Number of readmissions, days of hospitalization, months in service, days until first admission | The intervention group showed a significant reduction in readmissions and spent fewer days hospitalized compared to the control group. |
| Calsyn et al. (1998); USA | RCT  Modified PACT / ACT for homeless people with SMI (homelessness-adapted ACT model) — ACT programs modified from the original PACT model to include special outreach services and housing assistance for homeless individuals. | Study 1 N = N.D. Medical diagnosis: SMI   - Schizophrenia: 30% - Major depression: 21% - Bipolar disorder: 9% - Other psychoses: 5% - Other disorders: 15%   Age: Mean = 33.7 years Sex: 58% male  Groups: N/A  Study 2 N = 165 Medical diagnosis: SMI   - Schizophrenia: 66% - Delusional/paranoid disorder: 3% - Bipolar disorder: 13% - Atypical psychosis: 12% - Recurrent depression: 15% - Dementias: 1.2%   Age: Mean = 34.76 years, SD = 10.41 Sex: 58% male  Groups:  ACT-CW (ACT + community workers; paraprofessional augmentation): n = 55  ACT-only (standard ACT): n = 55   - Broker Case Management group = 55 | - Homeless or at risk of homelessness. - Severe DSM-III-R Axis I diagnosis. - Willingness to receive support in mental health, housing, employment, and social services. | Urban | N.D. | - Size of professional and natural support networks - Emotional, material, recreational and advisory support - Number of conflictive relationships | ACT increased access to professional support but did not lead to significant improvements in participants’ natural social relationships. While community workers within ACT provided more material assistance, they did not enhance other dimensions of social interaction. To improve social integration for homeless individuals with mental illness, it is recommended that ACT programs include targeted social skills interventions. |
| Carton et al. (2010); USA | Observational. Descriptive cross-sectional study.  Classic ACT (high-fidelity ACT program; HACT—Hillsborough Assertive Community Treatment) — ACT services for formerly homeless persons (Tampa, Florida, USA), with fidelity monitored using DACTS. | N = 22 Medical diagnosis: SMI Age: Mean = 41 years, SD = 10.69 Sex: 72.2% men  Groups: N/A | - Formerly homeless individuals - Receiving ACT services (HACT, Hillsborough) - With at least 6 months of program participation | Urban | N.D. | - Perceived Amount of Social Support: Adapted SSQSR questionnaire - Quality of Social Support: QRISS scale (adapted version, support items only) - Replacement of Previous Relationships: Dichotomous question: “Have the relationships with the HACT team replaced those you had during homelessness?” | The HACT team was identified as the primary and highest-quality source of social support for formerly homeless individuals with severe mental illness. Relationships with the team were reported to be stronger than those maintained before or during homelessness. In many cases, ACT professionals took the place of friends, family, or other prior social contacts. While this was viewed as a positive outcome in the short term, the authors recommend promoting the development of natural social networks over time. |
| Chandler et al. (1999); USA | RCT (cost-effectiveness analysis)  Classic/high-fidelity ACT (capitated ACT model; STRIDES program) — a capitated Assertive Community Treatment program closely modeled on ACT, designed for high service users in a county mental health system | N = 59 Medical diagnosis: SMI Age: Over 45 — ACT group: 30%; Control group: 48.3% Sex:   - ACT group: 60% male - Control group: 62.1% male   Groups:   - N intervention group (ACT) = 30 - N control group = 29 | - Residents in subacute intensive care units - Expected stay ≥4 to 6 months - High previous use of the mental health system | Urban | May 1995 - April 1996 | - Costs: gross and net (excluding Medicaid reimbursement) - Community stay (days outside the hospital) - Number of admissions and hospital days - Outpatient care and continuity of care - Housing and employment status - Satisfaction, quality of life (Lehman scale) - Level of functioning (SLOF) | ACT was significantly more cost-effective than usual services, with much lower costs per participant. Participants in the ACT group spent more time living in the community and had fewer long-term hospital readmissions, although they used emergency services more frequently. Continuity of care after hospitalization was notably better in the ACT group, with far more provider contacts prior to discharge. Several ACT participants achieved independent housing, unlike those in the control group. Global functioning improved significantly in the ACT group. However, no significant differences were found in satisfaction, quality of life, or social activities, which were slightly higher in the control group. |
| Chandler et al. (2002); USA | RCT (cost-effectiveness analysis)  Classic ACT (capitated ACT program; STRIDES) — capitated ACT model designed for very high and sustained service utilizers, focused on substituting intensive community services for long-term/sub-acute institutional care. | N = 59 Medical diagnosis: SMI Age: Over 45 — ACT group: 30%; Control group: 48.3% Sex:   - ACT group: 60% male - Control group: 62.1% male   Groups:   - N intervention group (ACT) = 30 - N control group = 29 | - Cost to the system >$40,000 in 2 of the last 3 years - Frequent readmissions - Inability to live independently - Expected stay in subacute unit of at least 4–6 months | Urban | Intervention started in May 1995. Duration: 4 years | - Costs (net and gross) - Hospitalization days - Hours of outpatient services/cases managed - Discharge from subacute units - Admission to Step-Up program (transition to lower intensity) | ACT led to a significant reduction in the number of days spent in institutions and resulted in lower net annual costs, generating substantial savings over the study period. The program also provided a greater amount and better continuity of outpatient services. Several participants successfully transitioned to lower-intensity levels of care. Importantly, ACT helped prevent reliance on a "crisis-only care" model. |
| Chow et al. (2011); Canada/Japan | Observational. Cross-sectional comparative study.  Classic ACT (ethnoculturally adapted ACT teams; cross-national comparison) — Mount Sinai Hospital ACT (Toronto, Canada) vs KUINA Center ACT (Hitachinaka, Japan), both with good ACT fidelity (DACTS). | N = 106 Medical diagnosis: SMI Age:   - Mount Sinai Hospital ACT: Mean = 37.5 years, SD = 9.1 - Kuina ACT Center: Mean = 39.4 years, SD = 11.8   Sex:   - Mount Sinai Hospital ACT: 71% men - Kuina ACT Center: 80% men   Groups:   - N Mount Sinai ACT (Canada) = 66 - N KUINA Center ACT (Japan) = 40 | Not specified: under ACT follow-up | Urban | 2002–2005 | - Days of hospitalization - Number of admissions - Number of patients with at least one admission - Psychiatric symptoms (BPRS) - Patient and family satisfaction - Fidelity to the ACT model (DACTS) | The ACT model, when culturally adapted, has proven effective in both multicultural settings like Canada and more homogeneous contexts like Japan. Key adaptations included the use of bicultural staff, incorporation of traditional practices, strong family involvement, and collaboration with local communities. In both settings, teams achieved significant reductions in hospitalizations and symptoms, along with high levels of participant satisfaction. |
| Clark et al. (1998); USA | RCT (cost-effectiveness analysis)  Classic ACT (Dual Disorders ACT / Integrated ACT) vs Standard Case Management (SCM) — ACT teams with specific training in integrated substance use treatment. | N = 193 Medical diagnosis: Dual diagnosis   - Schizophrenia: 53.6% - Bipolar disorder: 23.6% - Schizoaffective disorder: 22.7%   Age: Mean = 34 years Sex: 74% men  Groups:   - N Intervention group (ACT) = 100 - N Control group (SCM) = 93 | - DSM-III-R diagnosis of schizophrenia, schizoaffective disorder or bipolar disorder - Active substance use disorder - Aged 18–60 years - No severe medical comorbidities - No severe learning disability - Written informed consent obtained | Mixed | 1989–1991. Follow-up: 3 years (until 1994) | - SATS (Substance Abuse Treatment Scale). - Subjective Quality of Life (QOL) - Residential stability - Arrests and use of legal system - Family and informal support - Costs (social perspective): mental health, general medical care, legal system community services, informal care | Both groups showed improvements in substance use and quality of life. During the first two years, ACT was not significantly more cost-effective than Supportive Case Management (SCM). However, by the third year, ACT demonstrated greater efficiency, with a lower cost per unit of improvement. |
| Clarke et al. (2000); USA | RCT  Classic ACT (two start-up ACT teams) vs Usual Care — RCT comparing two ACT variants (consumer-staffed vs non-consumer-staffed) against usual community care. Fidelity measured with Dartmouth ACT Fidelity Scale. | N = 163 Medical diagnosis: SMI Age: Mean = 36.5 years, SD = 10.3 Sex: 60.7% male  Groups:   - N Consumer ACT team = 57 (63.2% male) - N Non-consumer ACT team = 57 (61.4% male) - N Usual care team = 49 (57.1% male) | - Aged ≥18 years. - Diagnosis of SMI. - Functional impairment in at least two areas: social role, daily living activities, or social acceptance. - Individuals with intellectual disability were excluded. | Urban | 18 months before study start (1997–1998). Follow-up: 24 months post-randomization | To assess whether ACT teams (one composed of mental health service users, the other of non-user professionals) reduce risk or delay time until:   - First psychiatric hospitalization - First psychiatric emergency visit (ER) - First episode of homelessness - First arrest | No significant differences were found between ACT and Usual Care in terms of hospitalizations, emergency visits, or time spent homeless. However, individuals in the Usual Care group experienced a shorter time to first arrest compared to those in ACT groups. Among ACT participants, those supported by consumer staff had fewer hospitalizations than those in traditional ACT teams. Higher BPRS scores were associated with an increased risk of hospitalization and emergency visits. A strong therapeutic relationship with providers was linked to a lower risk of homelessness. |
| Clausen et al. (2015); Norway | Observational. Cross-sectional study.  Classic ACT (Norwegian ACT teams; national implementation context) — patients enrolled in 12 ACT teams established in Norway (2009–2011 national rollout) | N = 149 Medical diagnosis: SMI   - Schizophrenia: 80%   Age: Mean = 38 years, SD = 10.5 Sex: 68% men  Groups: N/A | Individuals with SMI treated by ACT during its first year of implementation | Mixed | December 2009 - February 2011 | - Quality of Life: MANSA (Manchester Short Assessment of Quality of Life) - Functioning: PSF (Practical and Social Functioning, revised version) - Global functioning: GAF - Psychiatric symptoms: BPRS - Substance abuse: AUDIT and DUDIT - Weekly social contact. | Better quality of life was associated with older age, higher functioning, and frequent social contact. In contrast, anxiety and depression symptoms were linked to poorer quality of life. Specific areas of functioning—such as personal hygiene, financial management, and social networks—were related to greater satisfaction in corresponding life domains like health, housing, and relationships. No significant associations were found with psychotic symptoms or gender. |
| Clausen et al. (2016); Norway | Observational. Naturalistic pre-post study.  Classic ACT (Norwegian ACT teams; low–moderate to moderate–high fidelity, TMACT) — observational cohort across 12 Norwegian ACT teams, with explicit fidelity evaluation using TMACT. | N = 142 Medical diagnosis: SMI Age: Mean = 39.8 years, SD = 10.6 Sex: 67% men  Groups: N/A | - Aged ≥18 years - Severe mental disorder (schizophrenia, schizoaffective, other psychotic disorder, bipolar disorder) - Impaired functional level - Need for comprehensive and long-term follow-up by mental health and social care services - Problematic substance use was allowed if not the primary diagnosis | Mixed | ACT treatment initiated in December 2009 and February 2011. Follow-up: 2 years before and 2 years after | - Number of hospital admissions. - Total days hospitalized - Days of involuntary admission - GAF (symptoms and functioning) - BPRS (psychiatric symptoms) - PSF (practical and social functioning) - AUDIT / DUDIT, AUS / DUS (substance use evaluation) | Both groups experienced a significant reduction in total hospital days, with the group with substance use showing a more pronounced decrease. Involuntary admission days were significantly reduced only among individuals with substance use. There were no significant changes in the total number of admissions for either group. A significant interaction was found between substance use and baseline functioning: individuals with substance use and higher functioning at baseline showed a greater reduction in inpatient days, whereas among those without substance use, better functioning was associated with more inpatient days after ACT. |
| Clausen et al. (2016); Norway | Observational. Naturalistic longitudinal study.  Classic ACT (Norwegian ACT teams; national rollout; fidelity measured with TMACT) — same Norwegian 12-team ACT implementation, now focused on differential outcomes by baseline inpatient utilization. | N = 142 Medical diagnosis: SMI Age:   - High use: Mean = 38 years, SD = 9.7 - Non‑high users: Mean = 42 years, SD = 10.8   Sex:   - High use: 69% men - Non‑high users: 64% men   Groups:   - N High users group = 74 - N Non‑high users group = 68 | - Aged ≥18 years - SMI - Low psychosocial functioning - Need for long-term follow-up - Comorbid substance use was permitted (not as primary diagnosis) | Mixed | December 2009 – February 2011. Follow-up: 2 years before and 2 years after admission | - Number of hospital admissions - Total days of hospitalization - Days of involuntary hospitalization - Symptomatology (BPRS: positive symptoms, negative symptoms, mania, anxiety) - Global functioning (GAF) and functional performance (PSF) - Problematic alcohol/drug use (AUDIT/DUDIT) - Engagement with services (HEAS) - Involuntary outpatient treatment | Following the implementation of ACT, there was a significant reduction in both total and involuntary hospitalization days across the overall sample. High service users experienced a strong decline in both total and involuntary hospital days, while low users showed an initial increase in hospitalization during the first year, followed by a decrease in the second year. Factors associated with reduced hospitalization included more severe negative symptoms, being under involuntary outpatient treatment at the time of ACT enrollment, and a history of frequent service use. |
| Clausen et al. (2020); Norway | Observational. Prospective cohort study.  Classic ACT (Norwegian national ACT teams; fidelity assessed via TMACT) | N = 142 Medical diagnosis: SMI with substance use problems   - Schizophrenia spectrum disorders: 87% - Bipolar disorder: 7%   Age: Mean = 40 years, SD = 8.7 Sex: 67% men  Groups: N/A | - Participants from the first 12 ACT teams in Norway - Diagnosis of SMI - Follow-up of at least 2 years - Initial classification based on problematic substance use using AUDIT/DUDIT or clinical scales (AUS/DUS) | Mixed | December 2009 – February 2012. 24-month follow-up | - Adequate housing - Meaningful daily activity (employment, training...) - Psychiatric symptoms (BPRS-E, including subscales: positive symptoms, negative symptoms, mania/agitation, anxiety/depression) - Global functioning (GAF-F) and practical/social functioning (PSF) - Quality of life (MANSA: overall mean and satisfaction with life in general) | Both groups showed improvements in housing stability, psychiatric symptoms (particularly reductions in anxiety, depression, and mania) and global functioning. A significant reduction in manic symptoms was observed only in the group with problematic substance use. No significant changes were found in meaningful daily activity, practical or social functioning, or overall life satisfaction, although a slight improvement in life satisfaction was noted in the group without problematic substance use. |
| Coetzee et al. (2022); South Africa | Observational. Retrospective cohort study.  Modified ACT (resource-adapted; larger caseload; less frequent visits; South Africa) | N = 55 Medical diagnosis: Schizophrenia or schizoaffective disorder Age: Mean = 43 years, SD = 9.69 Sex: 76% men  Groups:   - N Intervention group (ACT-i) = 30 - N Control group = 25 | - High-frequency psychiatric service users (HFUs) - Diagnosis of schizophrenia or schizoaffective disorder - Participants in the ACT trial in South Africa between January 2007 and March 2010 | Mixed | Inclusion in original study: 2007–2010, Review up to 2020 | - Number of psychiatric readmissions - Total number of hospitalization days (over 10 years) - Admission to SUSD (step up/setp down) services - Forensic evaluation - Death (with cause) - Group change (re-enrolment, discharge due to stability or lack of adherence) | Patients who initially received ACT demonstrated better outcomes, including a higher number of individuals without psychiatric readmissions and greater use of available services. The modified ACT model appeared to provide sustained long-term benefits, even under suboptimal conditions such as high caseloads and less frequent visits. These findings suggest that the ACT approach may be effective in low-resource settings, though larger studies with more consistent control groups are needed to confirm these results. |
| Commander et al. (2005); United Kingdom | Observational. Prospective longitudinal study.  Assertive Outreach (PACT-based; high fidelity; UK routine implementation) | N = 250 Medical diagnosis: SMI   - Schizophrenia, schizotypal or delusional disorder: 74% - Bipolar affective disorder: 22% - Other: 4%   Age: 71% between 25–44 years Sex: 74% men  Groups: N/A | - Aged 18–65 years - Severe and enduring mental illness - ≥2 hospital admissions in the past 2 years - ≥3 of the following: nonadherence, history of violence or offences, persistent risk of self-harm or neglect, treatment resistance, substance abuse, previous compulsory admission | Urban | 1999-2002 | - BPRS scale (psychiatric symptoms) - FACE (social and mental functioning, risk, behavior) - Number of hospital admissions - Days of hospitalization - Involuntary admissions - Adherence - Treatment response | A significant reduction in hospitalizations was observed, including both overall admission rates and involuntary admissions. The total number of hospitalization days also decreased substantially. However, no improvements were noted in psychiatric symptoms or social functioning. In fact, there was a significant decline in performance of daily living activities. While there was a moderate reduction in treatment non-response, no changes were observed in risks to self or others. |
| Cusack et al. (2010); USA | RCT  Forensic ACT (FACT; high-fidelity ACT adaptation for criminal justice settings) | N = 134 Medical diagnosis: SMI Age:   - FACT group: Mean = 38.8 years, SD = 10.9 - TAU group: Mean = 34.4 years, SD = 8.9   Sex: 59% male  Groups:   - N Intervention group (FACT) = 72 - N Control group (TAU) = 62 | - Aged ≥18 years. - Diagnosis of SMI. - Incarceration within the past year. - Criminal justice system involvement (e.g., detention, convictions, prior arrests). - No history of serious violent crimes (e.g., murder, rape, aggravated assault). | Urban | 2000 – 2003. Follow-up: 12 and 24 months post-randomization | - Number of arrests - Number of days in jail - Number of convictions - Number of hospitalization days - Number of outpatient visits - Number of recorded psychiatric crises - Costs of psychiatric hospitalization - Outpatient visit costs - Incarceration costs | The FACT group experienced fewer arrests and spent less time in jail compared to the TAU group. They also had fewer hospital days and a greater number of outpatient visits. Although outpatient service costs were higher for the FACT group, these were offset by reductions in hospitalization and incarceration costs. Overall, FACT proved more effective in keeping participants out of the judicial system without increasing overall mental health expenditures. |
| Dahlan et al. (2013); Malaysia | Observational. Descriptive cross-sectional study  ACT (classic / ACT-style community treatment) — ACT implemented as part of community mental health reform in Malaysia | N = 155 Medical diagnosis: Schizophrenia Age: Median = 36 years (IQR 29–44) Sex: 72.3% men  Groups: N/A | - Diagnosis of schizophrenia (DSM-IV-TR) - Received ACT for at least 1 year - Aged 18 - 60 years | Urban | February to May 2010 | - Symptom remission (abbreviated BPRS according to Andreasen et al. criteria) - Social support (clinically assessed: neighborhood relationships, social acceptance, social participation) - Duration of illness and ACT treatment - Type of antipsychotic - Treatment adherence - Substance use - Quality of life (WHOQOL-BREF) - Level of functioning (PSP) - Frequency of hospitalization | A large proportion of participants achieved symptom remission. Strong social support and higher educational attainment were significantly associated with better outcomes. Additional factors linked to remission included shorter illness duration and being under the age of 35. No significant associations were found with antipsychotic type, treatment adherence, or substance use. Most participants demonstrated good functioning, low hospitalization rates, and favorable quality of life scores across multiple domains. |
| Daubney et al. (2021); Australia | Observational. Prospective pre-post study (longitudinal, without control group)  ACT-derived adaptation (Youth ACT / Assertive Outreach for adolescents): AMYOS (Assertive Mobile Youth Outreach Service) | N = 243 Medical diagnosis: Severe and complex mental health problems F40–F48: Neurotic, stress-related and somatoform disorders: 46.5% Age: Mean = 15.71 years, SD = 1.45 Sex: 34.6% male  Groups: N/A | - Aged 13-18 years - Severe and complex mental health problems - High-risk behaviors or risk of deterioration - Difficulty engaging with traditional services - Need for more intensive intervention | Urban | 2014 – October 2019 | - Global functioning (CGAS) - Psychiatric problems (HoNOSCA) - Suicidality and hostility (BPRS) - Suicidal risk and risk behaviors (frequency) - Therapeutic engagement (engagement scales) - Participation in education, employment, primary care - Hospitalization: number of monthly admissions, number of days hospitalized, emergency room visits | Significant improvements were observed in global functioning, reductions in psychiatric symptoms, and decreases in suicidal risk and related behaviors. Both families and adolescents showed greater engagement with services. Hospitalizations decreased, with fewer monthly admissions and nights spent hospitalized. Although emergency visits increased, hospital admissions did not. There were also notable increases in educational participation, employment rates, and contact with primary care physicians. |
| de Bruijn et al. (2023); Netherlands | Observational. Retrospective cohort study.  Flexible Assertive Community Treatment (FACT) — Dutch FACT model (fully implemented, system‑wide FACT teams). | N = 1734 Medical diagnosis: Psychotic disorder   - Schizophrenia: 46.8% - Other psychotic disorder: 39.6% - Schizoaffective disorders: 13.6%   Age: 35–50 years: 42.8% Sex: 65.1% men  Groups:   - N No discharge group (n = 1067), 65.4% men - N Discharged control group (TAU) (n = 667), 64.5% men | - Diagnosis of psychotic disorder (DSM-IV-TR or DSM-5) - At least one uninterrupted year of FACT treatment (no gaps >30 days) - FACT treatment received between 2009 and 2019 - Informed consent | Urban | 2009–2010 | - Clinics: hospitalization in first year, HoNOS - Use of services: total contact hours, percentage of contacts in community, multidisciplinary (no. of disciplines involved) - Contacts with other teams (outside FACT). | The most common discharge profile included older age at onset, diagnosis of a psychotic disorder other than schizophrenia, lower initial HoNOS scores, and fewer contacts with non-FACT teams. Planned discharge occurred in just over one-third of cases. Median time in FACT was shorter for those discharged compared to those not discharged. Factors such as treatment intensity, multidisciplinary approach, and proportion of community contacts were not associated with discharge status. |
| Dekker et al. (2002); Netherlands | RCT  ACT-derived adaptation (Dutch ACT-like intensive community case management; partial ACT fidelity / modified ACT) | N = 79 Medical diagnosis: SMI Age: Mean = 40 years Sex: Not determined  Groups:   - N Intervention group (ACT) = 51 - N Control group = 28 | - Chronic psychiatric diagnosis. - History of multiple psychiatric admissions. - Known to several mental health agencies. - Difficulties with daily living (requiring support for housing and daily activities). | Urban | Patient recruitment between July 1993 and June 1994. Follow-up: 2 years | - Number of psychiatric hospitalizations - Total number of hospital days - Psychiatric symptoms and social functioning | ACT in Amsterdam significantly reduced hospital days but did not prevent psychiatric admissions. There were no significant improvements in psychiatric symptoms or social functioning compared to standard care. The absence of 24/7 coverage in ACT may reduce its effectiveness in preventing crises. Long-term follow-up is recommended to better assess the impact on patient stability. |
| Dincin et al. (1993); USA | Observational. Retrospective cohort study.  Classic ACT (Stein & Test-derived ACT / Thresholds Bridge ACT program) — early urban ACT implementation with shared team responsibility and in vivo services. | N = 66 Medical diagnosis: SMI   - Schizophrenia: 82% - Major affective disorder: 18%   Age: Mean = 34.6 years, SD = 11.1 Sex: 65% men  Groups: N/A | - Individuals with ≥3 state hospital admissions in the past year, or ≥5 lifetime hospitalizations, or ≥100 hospital days in the past year. - All identified from the target area hospital. | Urban | 1987–1989 Seguimiento de pacientes: 1 y 2 años tras inclusión | - Number of hospitalizations in state hospital - Hospitalization days - GAS (Global Assessment Scale) - Hospital and program costs | ACT significantly reduced hospital use at both individual and system levels. It also improved participants’ global functioning. The program demonstrated high feasibility, even in regions with a history of high bed usage. Regular monthly inter-agency meetings were essential for coordinating services and reducing barriers. |
| Dixon et al. (1997); USA | Observational. Prospective study.  Classic ACT (homelessness-focused ACT; ACT arm of an RCT) — assertive community treatment program model delivered to homeless adults with severe and persistent mental illness. | N = 77 Medical diagnosis: SMI Age: Mean = 40.9 years, SD = 10.0 Sex: 68% men  Groups: N/A | - Homeless at intake - Severe and persistent mental illness - Aged between 18 and 64 years | Urban | N.D. | - Compliance with medication (antipsychotics, antidepressants, mood stabilizers) - Psychiatric symptoms (BPRS scale) - Housing status - Hospitalizations | Medication compliance at baseline was low but showed a significant increase after three months and was largely maintained throughout the year. Patients who remained compliant exhibited fewer psychiatric symptoms. No significant differences were found in hospitalization days, housing status, or incarceration rates. In conclusion, voluntary ACT treatment improved medication adherence among homeless individuals with severe mental illness, although about one-third remained noncompliant at some point during the year. |
| Doré-Gauthier et al. (2020); Canada | Quasi-experimental study with historical controls.  ACT-derived specialized adaptation (Modified ACT / Intensive Assertive Community Intervention Team)  IACIT-HYPS (Intensive Assertive Community Intervention Team for Homeless Youth with Psychosis and SUD) integrated within Early Intervention Services (EIS). | N = 50 Medical diagnosis: Affective or non-affective psychosis Age:   - IHE group: Mean = 24.1 years, SD = 3.0 - TAU group: Mean = 23.6 years, SD = 3.5   Sex:   - IHE: 91.7% men - TAU: 92.3% men   Groups:   - Intervention group (IHE, EIS + IACIT-HYPS) = 24 - Control group (TAU) = 26 | - Diagnosis of affective or non-affective psychosis. - Aged 18 - 30 years. - No previous treatment for psychosis, or treatment for less than one year. - Additional criteria for the IACIT-HYPS group: Homeless or at risk of homelessness and diagnosis of a substance use disorder (SUD). | Urban | IHE: February 2012 – October 2015 TAU (historical cohort): 2005 – 2011 24-month follow-up | - SOFAS (social and occupational functioning) - GAF (global functioning) - CGI-S (clinical global impression) AUS and DUS (alcohol and drug use) - Stability and autonomy in housing - Number and duration of hospitalizations - Emergency room visits - Antipsychotic treatment | Participants in the IHE group were more likely to achieve housing stability and did so more quickly compared to the control group. They also spent less time hospitalized during follow-up. No significant differences were found between groups in psychiatric symptoms, social functioning, or global clinical functioning. Both groups showed similar reductions in substance use. However, cocaine abuse was associated with a lower likelihood of achieving housing stability. |
| Drake et al. (1998); USA | RCT  Classic ACT (Integrated Dual Disorders ACT / ACT for co-occurring SMI–SUD) — ACT team delivering integrated mental health + substance abuse treatment as part of the ACT model, compared with Standard Case Management (SCM). | N = 223 Medical diagnosis: SMI Age:   - ACT group: Mean = 34 years, SD = 8.5 - Control group: Mean = 41 years, SD = 11   Sex:   - ACT group: 74.4% male - Control group: 54.7% male   Groups:   - N Intervention group (ACT) = 100 - N Control group = 93 | - Diagnosis of schizophrenia, schizoaffective disorder, or bipolar disorder according to DSM-III-R. - Active substance use disorder. - Age between 18 and 60 years. - No severe medical comorbidities. - No intellectual disability. - Written informed consent provided. | Mixed | 1989 – 1995 | - Alcohol Use Scale (AUS) - Drug Use Scale (DUS) - Addiction Severity Index (ASI) - Substance Abuse Treatment Scale (SATS) - Days of use in past 6 months: Time-Line Follow-Back (TLFB) - Drug use detection - Expanded Brief Psychiatric Rating Scale (BPRS) - Global Assessment of Functioning (GAF) - Quality of Life Interview (QOLI) - Institutionalization and hospital history - Service Utilization Interview - Program participation: attendance, medication adherence, recovery engagement | ACT helped patients progress more quickly in their recovery process but did not increase the number of patients who stopped substance use. Both ACT and the comparison treatment achieved similar reductions in substance use and hospitalizations, with no significant differences between them. |
| Drukker et al. (2011); Netherlands | Quasi-experimental study with matched controls  ACT-model classification: ACT-derived adaptation (Flexible/FACT variant; “Dutch variant of ACT”) | N = 444 Medical diagnosis: SMI Age:   - FACT group: Mean = 43.1 years, SD = 1.1 - CAU group: Mean = 43.3 years, SD = 0.6   Sex:   - FACT group: 55% men - CAU group: 59% men   Groups:   - N Intervention group (FACT) = 114 - N Control group (CAU) = 330 | - Diagnosis of SMI or moderate mental illness - Patients monitored in the CNCM system (needs monitoring). Three baseline states: new, new episode, or persistent care - FACT group: patients assessed between July and December 2004 - Control group: patients from the NN region without FACT ≥1 year of available pre-study data. | Urban | 1999–2005 | - Days of psychiatric hospitalization, outpatient contacts, days in day hospital - Functioning: GAF-p (psychopathology) and GAF-i (impairment) - Cost-effectiveness: Cohort migration model and 5-year cost estimation - State classification: Long-term admission, short-term admission, outpatient care, supported housing | FACT resulted in an increase in outpatient contacts per year. No significant differences were found in hospitalization days or day hospital use. According to the cohort model, FACT reduced long-term admissions but increased short-term admissions and supported housing, as well as total outpatient contacts. Estimated costs over five years showed FACT was more expensive than care as usual. Functioning scores were slightly lower in the FACT group according to the model, though this was not confirmed in the usual care group. In conclusion, FACT increases outpatient service use, but its cost-effectiveness remains uncertain due to limited comparable clinical data. |
| Drukker et al. (2014); Netherlands | Observational. Retrospective pre-post study.  ACT-model classification: Classic ACT (Stein & Test / PACT lineage) | N = 214 Medical diagnosis: SMI Age: Mean = 37.4 years, SD = 11.4 Sex: 76% men  Groups:   - N Pre-ACT group = 213 - N ACT group = 214 - N Post-ACT group = 70 | - Difficulties accessing or remaining in treatment within the mental health system. - Lack of stable housing or homelessness. - History of multiple rejections or exclusions from mental health services due to: - Failure to attend appointments - Aggressive or antisocial behavior - Need for multidisciplinary care not coordinated by conventional services | Urban | 1999 – 2009 | - Number of hospital days and number of psychiatric admissions. - Number of days in supervised housing. - Number of mental health outpatient visits and frequency of contact with the ACT team. - Cost per patient based on the services used. | Patients in ACT increased their use of mental health services compared to the period before ACT. Use of supported housing also rose significantly during ACT. While hospitalizations became less frequent, the total length of stay did not decrease. Treatment costs increased during ACT, but greater patient stability was observed. After leaving ACT, many patients continued treatment, though hospitalizations increased. |
| Essock et al. (1998); USA | RCT (cost-effectiveness analysis)  Classic ACT (Stein & Test lineage; multidisciplinary ACT teams) vs Standard Case Management (SCM) | N = 262 Medical diagnosis: SMI   - Schizophrenia or schizoaffective disorder: 67% - Bipolar disorder: 17% - Major depression: 12% - Other disorders: 4%   Age: Not specified Sex: 64% men  Groups: N/A | - Diagnosis of severe Axis I mental disorder (schizophrenia, bipolar disorder, major depression, etc.) - High service use: ≥2 psychiatric hospitalizations, or 1 prolonged admission (≥180 days), or ≥2 crisis service contacts in the past 2 years - Community dysfunction: homelessness in the past year or weekly need for supervision/help with personal care | Urban | 1989 – 1993 | - Quality of Life Interview (QOLI) - Symptom Checklist-90-R (SCL-90-R) - HSRI Uniform Client Data - Family Burden Interview - Daily record of services (type, duration, staff, location) - Weekly overnight stay location reports - Costs | ACT increases the number of days individuals spend in the community without raising costs. It is particularly cost-effective for people who have been recently hospitalized. |
| Essock et al. (2006); USA | RCT  Classic ACT (high-fidelity ACT model) vs Standard Clinical Case Management (SCM) for integrated dual-diagnosis treatment (SMI + SUD) in homeless/unstably housed clients | N = 198 Medical diagnosis: SMI Age:   - ACT group: Mean = 36.4 years, SD = 7.9 - Control group: Mean = 36.6 years, SD = 7.7   Sex:   - ACT group: 71% male - Control group: 73% male   Groups:   - N Intervention group (ACT) = 99 - N Control group = 99 | - Diagnosis of major psychotic disorder. - Active substance use disorder. - Intensive use of health services in the past two years. - At least two of the following situations: - Psychiatric hospitalization - Crisis programs stay - Emergency department visit - Incarceration - Unstable housing or homelessness. - Impairment in independent living skills. - No pending: legal charges, severe medical illnesses, or severe intellectual disability. - If hospitalized, patients had to be scheduled for discharge to the community. - Capacity to provide informed consent. | Urban | August 1993 – July 1998. Follow-up: 3 years per participant | - Alcohol Use Scale (AUS) - Drug Use Scale (DUS) - Self-reported substance use days in past 6 months - Urine and saliva samples for alcohol and drug detection - Number of days in stable housing - Number of days in institutions (hospitals or prisons) - Total days hospitalized - Number of psychiatric hospitalizations - Brief Psychiatric Rating Scale (BPRS) - Global Assessment of Functioning (GAF) - Quality of Life Interview (QOLI) | Both groups showed improvements across multiple clinical domains over time. There were no significant differences between groups in reductions in substance use. In the site with higher institutionalization rates, ACT patients spent less time hospitalized than those receiving standard case management. No significant differences were found in psychiatric symptoms or satisfaction with care between the groups. ACT patients were less likely to disengage from the care system compared to those in standard case management. |
| Fam et al. (2007); Singapore | Observational. Retrospective cohort study.  Classic ACT (ACT / assertive outreach model; multidisciplinary community-based team) — IMH ACT programme (Singapore), based on Madison/Wisconsin ACT model principles. | N = 100 Medical diagnosis: SMI   - Schizophrenia: 86% - Schizoaffective disorder: 9% - Manic-depressive psychosis and others: 5%   Age: Mean = 42 years, SD = 9.9 Sex: 46% men  Groups: N/A | - Aged 18–65 years - Patients of the Institute of Mental Health - Severe and persistent mental illness (schizophrenia, delusional disorder, manic-depressive psychosis) - Presence of severe symptoms and significant functional impairment - Significant disability not adequately treated by traditional outpatient care - Three or more hospital admissions in the past year | Urban | November 2003 – December 2005 12-month follow-up | - Number of Admissions (NOA) - Length of Stay (LOS, in days) - Employment status (part-time or full-time employment vs. unemployment) | There was a significant reduction in both the number and duration of hospitalizations, along with a significant increase in patient employability after one year of intervention. |
| Fekete et al. (1998); USA | RCT  Rural ACT (modified classic ACT for rural settings; outreach ACT team). | N = 153 Medical diagnosis: SMI Age: Mean = 38.1 years, SD = 11.1 Sex: 43% male  Groups:   - N Intervention group (ACT) = 78 - N Control group = 75 | - Aged ≥18 years. - Diagnosis of SMI as per Indiana state criteria. - Low utilization of community mental health services. - Presence of at least one of the following risk factors: - Frequent psychiatric hospitalizations - Recurrent psychiatric emergency visits - Prior legal problems - Residential instability | Rural | Not specified. Follow-up: 24 months | - Psychiatric hospital use over 24 months of follow-up - Number of hospitalizations - Total number of days hospitalized - Quality of life (measured via interviews and service satisfaction scales) Indiana Level of Functioning (LOF) - Brief Psychiatric Rating Scale (BPRS) - Attitudes towards medication (adherence scales) - Number of housing changes - Employment and occupational status - Number of jail days and police contacts | No significant differences were found between ACT and standard care in hospitalization reduction. However, professionals rated ACT patients as having better quality of life and functioning. ACT patients exhibited a slight reduction in psychiatric symptoms. No differences were observed between groups in employment or legal issues. ACT patients showed less residential stability in the second year, possibly due to efforts to transition them into more appropriate housing. |
| Firn et al. (2013); United Kingdom | Observational. Retrospective study.  Assertive Outreach (AO) dismantling / replacement by CMHT reinforced with FACT  (FACT-enhanced CMHT as an ACT-derived adaptation integrated into routine community mental health teams). | N = 112 Medical diagnosis: SMI   - Schizophrenia: 89% - Bipolar disorder: 10%   Age: Mean = 44 years and 4 months Sex: 69.6% men  Groups: N/A | - Received care for at least 1 year by assertive outreach teams - Transferred to and followed for 1 year in the new FACT model integrated within CMHT | Urban | AO: until October 2010 CMHT with FACT: November 2010 – October 2011 | - Number of hospital admissions. - Days of hospitalization (occupied bed days - OBD) - Use of CRHTT (intensive home care teams): face-to-face contacts, missed appointments (DNA), total number of annual contacts per patient. - Housing status (independent, temporary, supported). - Employment status | FACT significantly reduced hospital admissions and halved the total days of hospitalization compared to AO. Annual mean team contacts per patient were lower in FACT, with no significant difference in face-to-face contacts. Missed appointments increased slightly. There was no difference in crisis team contacts. Housing stability improved significantly, but employment rates did not, possibly due to changes in reporting methods. Estimated cost savings included substantial reductions in hospitalization and contact expenses, as well as direct savings from AO closure. |
| Firn et al. (2016); United Kingdom | Observational. Retrospective pre–post study.  Assertive Outreach dismantling / replacement with CMHT + FACT  FACT described as Dutch adaptation of ACT integrated into CMHTs (2-tier system) | N = 112 Medical diagnosis: SMI   - Schizophrenia: 89% - Bipolar disorder: 10%   Age: Mean = 44 years and 4 months Sex: 69.6% men  Groups: N/A | - Patients with at least 12 months of prior care from Assertive Outreach (AO) teams - Who continued with enhanced standard care for at least part of the follow-up (4 years) | Urban | Pre-FACT follow-up period: until October 2010. FACT follow-up: 2011–2014 | - Number of hospital admissions - Days of hospitalization (with and without leave) - Use of crisis resolution and home treatment teams (CRHTT): number of contacts, face-to-face contacts, Did Not Attend (DNA) appointments - Estimated costs (based on 2010/11 values) | There was a significant reduction in admissions and hospital days in the years following AO closure. Total contacts decreased, but the proportion of face-to-face contacts remained stable. Did Not Attend (DNA) rates showed a moderate increase. No significant changes occurred in the use of Crisis Resolution and Home Treatment Teams (CRHTT). Estimated savings resulted from reduced contacts and less intensive care. FACT effectively compensated for the closure of the AO model without worsening clinical outcomes. |
| Gold et al. (2012); USA | Observational. Retrospective longitudinal study.  Classic ACT (High-fidelity ACT; DACTS-rated) — longitudinal evaluation of an ACT team’s service delivery patterns over 4 years. | N = 121 Medical diagnosis: SMI   - Schizophrenia spectrum disorders: 58% - Major mood disorders: 42%   Age: Not specified Sex: 63% men  Groups: N/A | - Individuals with severe mental illness - At risk of rehospitalization or homelessness - Treated in a high-fidelity ACT program | Urban | April 1996 – March 2000 (48 months) | - Days of contact per customer per month. - Hours per contact day - Total hours of service per client per month - ACT fidelity (DACTS) - Psychiatric hospitalization rate - Community employment rate | Frequency of contact increased over the years, while the duration of each contact decreased. Fidelity to the ACT model remained consistently high. Employment rates improved during the same period. Hospitalization rates declined, with fewer patients being hospitalized and a reduction in the average length of stay. |
| Hackman et al. (2009); USA | Observational. Retrospective cohort study.  ACT (modified ACT / ACT with planned transition/step-down component) — ACT program delivering classic ACT services but with annual discharge evaluation / transition to less intensive CMHC services (time-limited/step-down practice). | N = 67 Medical diagnosis: SMI   - Schizophrenia or schizoaffective disorder: 64.1%   Age: Mean = 44.7 years Sex: 37.3% men  Groups: N/A | - Patients treated by the University of Maryland ACT between 1999 and 2003 - Referred to less intensive outpatient services (CMHC or CCT) within the same system - With at least one evaluation at the time of transfer - Complete medical record review | Urban | October 1999 – February 2003 | - Type of referral and follow-up. - Transition outcomes: success (remaining in outpatient services) vs. failure (returning to ACT or loss to follow-up). | A substantial proportion of patients with SMI can successfully transition from ACT to less intensive services. However, no clear predictors of successful transition have been identified. |
| Hambridge et al. (1994); Australia | Observational. Retrospective pre–post study.  Classic ACT / ACT-like outreach team (ACT-derived; Mobile Community Management Team – MCMT) | N = 64 Medical diagnosis: SMI   - Schizophrenia: 78% - Schizoaffective disorder: 5% - Bipolar affective disorder: 8% - Major depressive episode: 3% - Personality disorder: 6%   Age: Mean = 44.3 years Sex: 64% men  Groups: N/A | - Primary diagnosis of SMI - Aged >18 years - Frequent rehospitalizations or intensive use of community services - Failure in high-quality standard services - Residents of the catchment area - Follow-up of ≥12 months | Urban | May 1988 – July 1992 | - Hospitalization: number of admissions, days hospitalized, average length of stay - Life Skills Profile (LSP) - Brief Psychiatric Rating Scale (BPRS) - Dosage in chlorpromazine equivalents - Satisfaction: patient and family surveys | There was a substantial reduction in psychiatric hospitalization days and in the number of hospitalized patients. The average length of stay decreased significantly. Significant improvements were observed across all subscales of the LSP at six months, along with a marked reduction in non-psychotic symptoms. Medication dosage was also reduced over the course of the year. Despite the absence of a control group, positive outcomes were sustained, supported by high fidelity to the model and strong organizational backing. |
| Hamernik et al. (1999); Australia | Quasi-experimental study.  Classic ACT (pilot ACT team; assertive outreach, multidisciplinary, low caseload, extended hours + 24h backup) — ACT implemented in Brisbane, Australia. | N = 36 Medical diagnosis: Psychiatric diagnosis   - Schizophrenia: 71% - Bipolar disorder: 18% - Major depression: 5% - Borderline personality disorder: 5%   Age:   - ACT group: Mean = 34.5 years (range 21–59) - Control group: Mean = 42.5 years (range 24–64)   Sex:   - ACT: 61.1% men - Control: 55.6% men   Groups:   - N intervention group (ACT) = 18 - N control group = 18 | - Clear psychiatric diagnosis - Aged 16-64 years - ≥90 days of hospitalization in the previous year or ≥2 admissions in the last 12 months - Substance abuse or intellectual disability is not the primary problem | Urban | N.D. (12-month follow-up) | - Hospitalization days - Number of admissions - Life Skills Profile (LSP) - Brief Psychiatric Rating Scale (BPRS) - Quality of Life Scale (QLS) - Clinically Significant - Change Analysis (Jacobson & Truax) | Hospitalization days were reduced more in the ACT group compared to the control group, with both groups experiencing decreases in hospital admissions. Life skills improved in both groups, particularly in the ACT group. The ACT group also demonstrated a significant reduction in psychiatric symptoms. No significant changes in quality of life were observed, although a notable portion of the ACT group showed reliable clinical improvement. Clinically significant changes were more frequent in the ACT group than in the control group. |
| Hamilton et al. (2015); United Kingdom | Quasi-experimental study (Interrupted Time Series Analysis)  ACT-derived adaptation: Assertive Outreach Team (AOT / Assertive Outreach) – UK model | N = Not specified Medical diagnosis: Psychosis or Schizophrenia Age: Not specified Sex: Not specified  Groups: N/A | - Hospitalizations recorded in Hospital - Episode Statistics (HES) between April 1999 and December 2010 - Diagnosis of schizophrenia or psychosis - Analysis focused on the impact of the national introduction of AOT teams | Mixed | April 1999 - December 2010 | - Number of hospital admissions for psychosis/schizophrenia | A statistically significant reduction in hospital admission rates was observed following the implementation of the AOT model. A consistent seasonal pattern was noted, with peak admissions occurring in the summer months. However, causality cannot be definitively established, as the reduction may be due to the direct impact of AOT, a decrease in available psychiatric beds, or other simultaneous changes in community services. |
| Hansen et al. (2023); Denmark | RCT, 20-year follow-up  OPUS (Specialized Early Intervention Services; modified ACT-based model / ACT-derived EIS model) — early intervention program for first-episode schizophrenia spectrum disorder including modified ACT + psychosocial components | N = 164 Medical diagnosis: First-episode schizophrenia spectrum disorder Age: Mean = 45.9 years, SD = 5.6 Sex: 48.2% male  Groups:   - Patients who participated in the long term follow up (n = 164) - Patients who did not (n = 383) | - Diagnosis: First diagnosis of schizophrenia spectrum disorder according to the International Classification of Diseases (ICD-10: F20–F25, F28–F29). - Location: Residency within the Aarhus and Copenhagen catchment areas in Denmark. - No prolonged prior treatment: No antipsychotic treatment for more than 12 weeks before randomization. - Informed consent: Capacity to understand and agree to study participation. | Urban | 1998 and 2000, with follow-up until 2022 | - Psychiatric diagnosis (SCAN 2.1) - Number of psychiatric admissions, hospital days, and emergency visits - Outpatient psychiatric service contacts - Psychotic symptoms (SAPS) - Negative symptoms (SANS) - Functioning (GAF-F) - Personal and social functioning (PSP) - Cognition (Brief Assessment of Cognition in Schizophrenia) - Current psychiatric treatment (adherence, dosage, use) - Self-perceived recovery - Substance use - Suicidal ideation and attempts - Quality of life (WHOQOL-BREF) - Health-related quality of life (EQ-5D and VAS) - Self-rated health (Danish Health Profile 2013) | The 20-year follow-up found no significant differences between OPUS and TAU in symptoms, functioning, psychiatric service use, or clinical recovery. Although 40% of participants achieved symptomatic remission and 18% clinical recovery, most still required support. The need for new strategies to improve long-term outcomes is highlighted. |
| Harvey et al. (2011); Australia/United Kingdom | Observational. Cross-sectional comparative study.  Classic ACT / ACT-like teams (implementation/fidelity comparison study; DACTS-rated) — comparison of ACT implementation in Melbourne (Australia) vs London (England). | N = 622 Medical diagnosis: SMI Age:   - London: Mean = 36.1 years, SD = 12.0 - Melbourne: Mean = 37.4 years, SD = 11.54   Sex:   - London: 66.3% men - Melbourne: 63% men   Groups:   - N London ACT group = 439 - N Melbourne ACT group = 183 | - Individuals treated by ACT teams in Melbourne or London - Diagnosis of SMI - Data collected by clinical professionals based on medical records | Urban | Melbourne: August 2003 – December 2004 London: data from the PLAO study (2001) | - Model of care (DACTS) - Maslach Burnout Inventory - Minnesota Satisfaction Questionnaire - Job Diagnostic Survey - Adverse events: violence, self-injury, arrests, homelessness - Hospitalizations (frequency, duration) - Therapeutic Engagement (Helping Alliance Scale) - Substance use (Clinician Alcohol and Drug Scales) | Patients had similarities in age, gender, and diagnosis, although the Melbourne group had more previous hospitalizations, shorter average hospital stays (40 days compared to 70), higher levels of self-harm and homelessness, better therapeutic alliance, but fewer arrests than the London group. Staff showed similar levels of job satisfaction and burnout, though the Melbourne team had more ACT experience and more out-of-hours shift work. |
| Hastrup et al. (2013); Denmark | RCT (cost-effectiveness analysis)  OPUS (Early Intervention in Psychosis program incorporating modified/enriched ACT components) | N = 547 Medical diagnosis: First episode psychosis Age: Not specified Sex: Not specified  Groups:   - N Intervention group (OPUS) = 275 - N Control group = 272 | - Aged 18–45 years - Diagnosis within the schizophrenia spectrum (ICD-10 F2) - First contact with psychiatric services (inpatient or outpatient) - No antipsychotic treatment lasting more than 12 consecutive weeks prior to inclusion | Urban | January 1998 – December 2000. Follow-up until 2007 (5 years) | - GAF (Global Assessment of Functioning). - Health and social costs: hospitalization, outpatient care, emergency room visits, medications, supervised living, medical and psychological consultations | At two years, the OPUS group demonstrated significantly better global functioning than the control group, though this difference was not significant at five years. Total costs at five years were lower for OPUS but without statistical significance. OPUS participants used fewer hospitalizations and supervised living services but had more psychiatric outpatient visits. Simulation analyses suggest a high probability that OPUS is cost-effective, especially at common willingness-to-pay thresholds for improvements in global functioning. |
| Hastrup et al. (2015); Denmark | Quasi-experimental study with non-randomized control group.  Classic ACT (rural ACT team) + add-on components — with fidelity assessed. | N = 174 Medical diagnosis: SMI Age: Not specified Sex: Not specified  Groups:   - N Intervention group (ACT) = 86 - N Control group = 88 | - Primary diagnosis of F20, F22, F25 or F31 - And at least one of the following: - Substance use disorder (F10–F19) or forensic sanction (Z04.6) - ≥4 admissions or ≥50 hospital days in the past 2 years | Rural | 1996 and 2006. 4-year follow-up after ACT inclusion | - Direct costs (hospitalization, outpatient, medication, physicians, assisted living). - Retention in psychiatric services (contact up to 4 years). | ACT resulted in a significant reduction in total costs when assisted living expenses were included, with a notable decrease in hospitalization costs during the first two years. While retention rates at four years did not differ significantly, the model proved to be more economically efficient than standard care, supporting its implementation in rural areas of Denmark. |
| Herinckx et al. (1997); USA | RCT  Classic ACT (two ACT teams) vs Usual care | N = 174 Medical diagnosis: SMI Age: Mean = 36.5 years, SD = 10.3 Sex: 61% male  Groups:   - N Intervention group (ACT) = 116 - N Control group = 58 | - Aged ≥18 years. - Diagnosis of SMI according to Oregon state definition. - History of persistent psychotic symptoms (excluding substance-induced psychosis). - Functional impairment in at least two of the following areas: social life, daily activities, or social acceptability. | Urban | March 1994 – October 1995. Observation period: March 1996 – July 1996 | - Engagement and retention in treatment: Follow-up over 870 days to assess treatment retention - Number of days hospitalized - Number of psychiatric consultations - Diagnosis - History of substance use - Duration of homelessness prior to study entry | ACT groups retained a higher percentage of patients compared to standard care. The risk of treatment dropout was greatest during the first nine months. Recent homelessness increased the risk of dropout. Patients receiving standard care were more than twice as likely to drop out compared to those in ACT. No significant differences were observed between ACT programs with consumer teams and traditional ACT. |
| Horiuchi et al. (2006); Japan | Uncontrolled prospective pre-post study.  Classic ACT (ACT-J Japan; high/moderate fidelity, DACTS 4.1) | N = 43 Medical diagnosis: SMI   - Schizophrenia and related disorders: 67.7% - Mood disorders: 18.2% - Other diagnoses: 15.1%   Age: Mean = 33.9 years, SD = 10.2 Sex: 42.4% male  Groups: N/A | - Aged 18–59 years - Resident in one of the three cities in the catchment area - Diagnosis of SMI (schizophrenia or mood disorders) - History of: multiple hospitalizations or psychiatric emergency use; treatment nonadherence; problematic behaviors (violence, substance use, disappearances, homelessness, suicide attempts); lack of social roles or self-care skills during the past 2 years - Exclusion: primary diagnosis of intellectual disability, dementia, substance use disorder, or personality disorder | Urban | May 2003 – April 2004. 12-month follow-up | - Quality of Life (QOLI) - BPRS scale (psychiatric symptoms) - Days of hospitalization - Frequency of family contact - Monthly income - Level of overall subjective well-being - Level of satisfaction across life domains (family, leisure, housing, health, safety, etc. | Reduction in unnecessary hospital use was achieved without negatively impacting subjective quality of life. However, satisfaction with family relationships declined, which was linked to a higher number of hospitalization days, underscoring the need for more effective family interventions. |
| Huguelet et al. (2012); Switzerland | Observational. Prospective pre–post study.  Classic ACT (newly implemented ACT program; moderate fidelity—Dartmouth index 3.82 | N = 55 Medical diagnosis: SMI Age: Mean = 41.3 years, SD = 12.5 Sex: 56.4% men  Groups: N/A | - Severe and persistent mental illness - Frequent use of psychiatric services - Frequent readmissions (“revolving door” phenomenon) - Difficulty engaging in outpatient care | Urban | October 2007 – August 2009 (follow-up: 6–8 months) | - Psychiatric symptoms: BPRS - Social functioning: HoNOS, MCAS - Quality of life: WHOQOL - Therapeutic alliance: WAI - Satisfaction with services: VSSS-54 - Adherence to treatment: MARS - Recovery: RAS - Substance use: AUS, DUS, SATS - Daily interventions: RQC - Family burden: FEIS, PSI - Direct/indirect costs, time of attendance | Significant improvements were observed in psychiatric symptoms, functioning, quality of life, and family burden. Hospitalization days decreased among the most severe patients. Younger age was the strongest predictor of improvement, while older patients, particularly those with delusional disorder, showed no improvement or deterioration. Fewer family interventions were linked to worse outcomes. Family burden improved in areas such as daily attendance, economic costs, and crisis perception. |
| Ito et al. (2009); Japan | Observational. Retrospective pre-post study.  Classic ACT (ACT-J Japan; high fidelity; DACTS mean ≈ 4.0–4.1) | N = 43 Medical diagnosis: Schizophrenia or Major Affective Disorder   - Schizophrenia: 72% - Major Affective Disorder: 19% - Others: 9%   Age: Mean = 35.8 years, SD = 10.5 Sex: 44% men  Groups: N/A | - Aged 18–60 years - Primary diagnosis: schizophrenia or major affective disorder - One or more of the following: ≥1 hospitalization in the past 2 years, ≥3 emergency visits, >3 months treatment interruption, significant difficulty with independent living - Resident in Ichikawa City or surroundings - Approval by the responsible physician | Urban | May 2003 – April 2004. Follow-up: 1 year post-discharge | - Number of psychiatric hospitalization days (pre and post) - Psychiatric symptoms (BPRS) - Quality of life (QOLI) - Stable housing status (>6 months) - Monthly income - Frequency of family contact | There was a significant reduction in hospitalization days. Psychiatric symptoms remained stable, and quality of life was maintained. The percentage of participants with stable housing increased, as did their monthly income. However, the frequency of family contact decreased slightly. |
| Ito et al. (2011); Japan | RCT  Classic ACT (ACT-J Japan; high/moderate fidelity, DACTS = 4.1) | N = 118 Medical diagnosis: Schizophrenia, mood disorder or other mental disorders (ICD-10)   - Schizophrenia = 88%   Age: Mean = 40 years Sex: 50.9% male  Groups:   - N intervention group (ACT) = 59 - N control group = 59 | - Residency in one of three selected cities. - Aged 18 - 59 years. - Diagnosis of schizophrenia, mood disorder, or other mental disorders according to ICD-10. - Significant use of psychiatric services in the past 2 years, defined as: - Two or more psychiatric hospitalizations - 100 or more days of psychiatric hospitalization. - Three or more visits to psychiatric emergency departments. - Prolonged absence from outpatient appointments (more than 3 months without attending). - Social functioning impairment in the past year, defined as a Global Assessment of Functioning (GAF) score ≤50. - Patients with dementia, intellectual disability or personality disorders were excluded. | Urban | May 1, 2004 – April 30, 2007. 12-month follow-up | - Days of psychiatric hospitalization - Psychiatric symptoms (Brief Psychiatric Rating Scale – BPRS) - Social functioning (Global Assessment of Functioning – GAF) - Quality of life (Quality of Life Interview – QOL) - Service user satisfaction (Client Satisfaction Questionnaire – CSQ-8) | ACT led to a significant reduction in hospitalization days compared to the control group, although statistical analysis did not show a significant difference. Depressive symptoms improved significantly in the ACT group but not in the control group. User satisfaction was higher in the ACT group. No significant differences were found in social functioning or quality of life between groups. |
| Jerrell (1995); USA | RCT (cost-effectiveness analysis)  PACT adaptation / PACT-TCL model (ACT-derived classic PACT implementation) | N = 122 Medical diagnosis: SMI Age: Not specified Sex: Not specified  Groups: N/A | - Aged 18–59 years - Recently discharged after ≥2 hospitalizations or subacute care in the past year - At least 2 of the following: - Poor work history - Use of public assistance - Poor basic life skills - Absence of support network - Inappropriate social behaviors requiring psychiatric or judicial intervention | Urban | N.D. (24-month follow-up) | - Social Adjustment Scale (SAS) - Role Functioning Scale (RFS) - Psychiatric symptoms (C-DIS-R) - Costs of intensive care (hospitalization, residential services, etc.) - Costs of support services (outpatient treatment, housing, medication, etc.) | The adapted PACT model produced the best outcomes in psychosocial functioning and reduction of intensive care costs, without being more expensive than other models. The clinical model led to greater symptom reduction but had less impact on functioning. The broker model was more economical but less effective overall. Providing more support services was key to improving outcomes across all models. The importance of multidisciplinary teams and continuous follow-up was emphasized. |
| Jones (2002); United Kingdom | Observational. Retrospective pre-post study.  ACT (UK) / Assertive Outreach Team (ACT-derived UK implementation) | N = 55 Medical diagnosis: SMI Age: Mean = 39 years (range: 22–59) Sex: 66% men  Groups: N/A | - Must meet 1 item from each category:  1. Category: Serious Mental Illness  - Schizophrenia - Psychotic depression - Bipolar affective disorder - Drug/alcohol abuse* - Duration of illness >5 years*  2. Category: Illness Instability  - 2 years under Section 2 or 3 - 180 days hospitalized in 2 years - Multiple interventions by various professionals  3. Category: Illness Disability  - Housing instability - Nonadherence to treatment - Repeated missed appointments - Unemployment*  4. Category: Risk Factors  - Complex CPA - Multiple suicide attempts - History of violence or judicial contact. - Exploitation/self-neglect   (*) Not essential criteria, but may support inclusion | Mixed | N.D. | - Number of admissions - Days of hospitalization per admission - Total days hospitalized - Camberwell Assessment of Need (CAN) - HoNOS | There was a reduction in the number of admissions and total days hospitalized, but the average length of each admission remained unchanged. Some patients who had not been previously hospitalized were admitted during ACT, indicating that ACT identified previously unmet needs. |
| Kane et al. (2004); USA | Quasi-experimental study.  ACT-derived PACT enhancement model: PACT + Advanced Practice Psychiatric Nurses (APNs) + Consumer Peer Providers (CPPs). | N = 59 Medical diagnosis: SMI Age:   - NPACT group: Mean = 40.3 years, SD = 16.4 - PACT group: Mean = 42.86 years, SD = 12.2   Sex:   - NPACT group: 62.2% men - PACT group: 52.4% men   Groups:   - N NPACT group = 38 - N PACT group = 21 | - Aged 18–75 years - No dual diagnosis with intellectual disability - English-speaking - Corrected vision and hearing within normal limits | Rural | N.D. | - Psychiatric symptoms (PANSS) - Community adjustment (MCAS) - Disability (Disability Rating Form) - Physical symptoms (AIMS and PILL) - Healthy lifestyle (HPLP) - Consumer satisfaction (MHSIP) | Improvements were observed in both groups, but NPACT showed superior results in reducing psychiatric symptoms, enhancing community adjustment, and increasing consumer satisfaction. There was also a trend toward improvement in physical symptoms in the NPACT group. No significant short-term effects were found on healthy lifestyle practices. |
| Karow et al. (2012); Germany | Quasi-experimental (cost-effectiveness analysis)  High-fidelity ACT (DACTS) within integrated psychosis care (ACCESS Trial**)** | N = 120 Medical diagnosis: SMI Age:   - ACT group: Mean = 31.4 years, SD = 9.9 - Standard care group: Mean = 37.6 years, SD = 11.7   Sex:   - ACT group: 56.3% men - Standard care group: 57.1% men   Groups:   - N Intervention group (ACT) = 64 - Control group = 56 | - Aged 18–65 years - Diagnosis of first episode or multiple episodes of a schizophrenia spectrum disorder - New or ongoing treatment with immediate-release quetiapine (IR) - For multi-episode patients: at least one psychotic relapse with subsequent hospitalization due to nonadherence in the past 24 months | Urban | 2006–2007. 12-months follow-up | - Cost-effectiveness using QALYs (quality of life adjusted for years). - PANSS (positive and negative symptoms) - CGI-S (severity) - GAF (functioning) - EQ-5D - Q-LES-Q-18 - SWN-K (subjective well-being) | ACT was associated with significantly lower inpatient costs but higher outpatient costs compared to standard care, resulting in a nonsignificant reduction in total costs. The ACT group showed a statistically significant increase in quality-adjusted life years (QALYs). The incremental cost-effectiveness ratio (ICER) favored ACT, with a very high probability of being cost-effective below the common threshold per QALY gained. |
| Karow et al. (2020); Germany | Quasi-experimental (cost-effectiveness analysis)  IC-TACT (Integrated Care + Therapeutic ACT; ACCESS model) | N = 270 Medical diagnosis: Affective or non-affective psychosis Age:   - IC-TACT: Mean = 39.4 years, SD = 14.2 - SC: Mean = 37.6 years, SD = 11.7   Sex:   - IC-TACT: 44.4% men - SC: 57.1% men   Groups:   - N Intervention group (IC-TACT) = 214 - Control group (SC) = 56 | - Aged 18–65 years - Diagnosis of affective or non-affective psychosis - Patients with multiple episodes - Clinical history of previous admissions - Consecutively enrolled according to routine clinical practice - Compared to a historical control group | Urban | IC-TACT (ACCESS II): 2008–2014 SC control (ACCESS I): 2005–2008 12-months follow-up | - Total costs (12 months): inpatient, outpatient, medication. - Quality of life adjusted for years (QALY) - PANSS - CGI-S - GAF - EQ-5D - Q-LES-Q-18 - Number of admissions, hospital days, use of day hospital | Integrated treatment with IC-TACT significantly reduces total costs and improves quality of life compared to standard care in patients with severe, multi-episode psychosis. The intervention is cost-effective even when delivered by high-cost specialized teams. |
| Kenny et al. (2004); USA | RCT  ACT-model classification: Classic ACT (Stein & Test model); ACT-only and ACT + community workers arms combined for analysis vs brokered case management (BCM). | N = 165 Medical diagnosis: SMI Age: Mean = 34.76 years Sex: 58% male  Groups:   - N Intervention group (ACT) = 105 - N Control group (BCM) = 60 | - Homeless or at risk of homelessness (defined as street living, staying in shelters, or experiencing multiple episodes of housing instability). - Diagnosis of a SMI (DSM-III-R or DSM-IV). - Willingness to receive support for welfare, employment, housing, mental health, or other social services. - No conviction for serious violent crimes in the past year (e.g., homicide, rape, armed robbery, arson). | Urban | August 1993 – July 1998. Follow-up every 6 months for 3 years | - Brief Psychiatric Rating Scale (BPRS) - Addiction Severity Index (ASI) - Number of days in stable housing vs. days homeless - Number of contacts with treatment team - Medication adherence: Self-reported (≥16 days taking medication in the last month) | ACT was more effective than BCM in reducing psychiatric symptoms and improving housing stability. Medication adherence was higher in ACT, along with a greater number of contacts with the healthcare team. Hospitalizations were also reduced in the ACT group. |
| Killaspy et al. (2006); United Kingdom | RCT  Classic ACT vs CMHT (Community Mental Health Team, UK RCT; REACT) | N = 251 Medical diagnosis: SMI Age:   - ACT group = Mean = 38 years, SD = 11 - Control group = Mean = 40 years, SD = 11   Sex:   - ACT group = 62% male - Control group = 55% male   Groups:   - N Intervention group (ACT) = 127 - N Control group = 124 | - Living in independent or low-support accommodation in the Camden and Islington districts. - Under care of community mental health teams for at least 12 months with difficulties in treatment engagement. - Primary diagnosis of SMI. - High recent use of inpatient psychiatric care (≥100 consecutive days or ≥5 admissions in the past two years, or ≥50 consecutive days or ≥3 admissions in the past year). - Participants with substance use or personality disorders were accepted if these were secondary diagnoses. - Patients with organic brain damage were excluded. | Urban | July 1999 – July 2002 | - Number of psychiatric hospitalization days over 18 months (measured via hospital records) - Client Satisfaction Questionnaire (CSQ) - Expanded Brief Psychiatric Rating Scale (EBPRS) - HoNOS - Life Skills Profile (LSP) - Camberwell Assessment of Need Short Assessment Schedule (CANSAS) - Manchester Short Assessment of Quality of Life (MANSA) - Homeless Engagement and Acceptance Scale (HEAS) - Rating of Medication Influences Scale (RoMI) - Alcohol and Drug Scale | No significant differences were found in the number of hospitalization days between the two groups. Patients receiving assertive treatment demonstrated better engagement with services and reported higher satisfaction. No differences were observed in clinical functioning, social functioning, or quality of life. |
| Killaspy et al. (2009); United Kingdom | RCT, 36-month follow-up  ACT (UK Assertive Outreach team; ACT-informed implementation) vs CMHT | N = 251 Medical diagnosis: SMI Age:   - ACT group mean = 38 years, SD = 11 - Control group = 40 years, SD = 11   Sex:   - ACT group = 62% male; - Control group = 55% male   Groups:   - N intervention group (ACT) = 127 - N control group = 124 | - At least 100 consecutive days of hospitalization or five or more admissions in the past 2 years, or 50 consecutive days or three admissions in the past year. - Living independently. - Considered a hard-to-engage case by mental health teams for at least 12 months. | Urban | 1999 – 2002 (36 months after randomisation) | - Total days of psychiatric hospitalization - Number of hospital admissions - Involuntary admissions - Average days per admission (mean, SD) | No significant differences were observed in hospitalization use between ACT and CMHT at 36 months. Participants in ACT had more face-to-face contacts with the care team in the final months of follow-up. ACT also had fewer dropouts compared to CMHT. No significant differences were found between groups in adverse events such as deaths, self-harm, or incarcerations. |
| Killaspy et al. (2014); United Kingdom | RCT, 10-year follow-up  Classic ACT vs CMHT (REACT 10-year follow-up) | N = 251 Medical diagnosis: SMI Age:   - ACT group mean = 38 years, SD = 11 - Control group = 40 years, SD = 11   Sex:   - ACT group = 62% male; - Control group = 55% male   Groups:   - N intervention group (ACT) = 99 - N control group = 104 | - At least 100 consecutive days of hospitalization or ≥5 admissions in the past 2 years, or 50 consecutive days or ≥3 admissions in the past year. - Living independently. - Identified as a hard-to-engage case by mental health teams for at least 12 months. | Urban | 1999 – 2002 (10 years after randomisation) | - Total days of hospitalization, number of hospital admissions, days per admission, involuntary admissions, proportion with any admission - Current employment or occupation, employment or occupation over the last 10 years, participation in social or leisure activities, family contact, current supported housing, supported housing in the past 10 years. - Adverse events: no service contact, homelessness, physical assaults (against others, strangers) , sexual offence, arson, imprisonment,  self-harm, recurrent self-harm | No significant differences were found in clinical outcomes between ACT and CMHT at 10 years. The ACT group had a higher number of hospital days, suggesting that ACT retained patients with more complex needs, while CMHT served more stable patients. Social outcomes, including employment, social activities, and family contact, were similar in both groups. Use of Community Treatment Orders (CTOs) was greater in ACT, with patients under CTOs more likely to remain in ACT or be transferred to forensic services. |
| Kim et al. (2015); South Korea | Quasi-experimental study with matched control group.  Modified ACT case-management program (Korean ACT) vs standard case management | N = 64 Medical diagnosis: Schizophrenia, bipolar disorder or major depression Age:   - ACT group: Mean = 40.88 years, SD = 10.42 - Control group: Mean = 42.03 years, SD = 8.35   Sex:   - ACT group: 65.6% men - Control group: 71.9% men   Groups:   - N Intervention group (ACT) = 32 - N Control group = 32 | - Aged 18–60 years - Complicated issues related to medication, housing, employment, legal or family matters - Diagnosis of schizophrenia, bipolar disorder or major depression - Informed consent from the patient and family | Urban | August 2011 – November 2012(15 months follow-up) | - Psychiatric Symptoms: BPRS Global - Functioning: GAF - Life Satisfaction Scale: Life Satisfaction Scale - Recovery-Promoting Relationship Scale (RPRS) | Within the ACT group, there was a significant reduction in psychiatric symptoms, though this was not superior to the control group. Global functioning improved significantly in the ACT group and was better than in the control group. No differences were observed between groups in life satisfaction. The control group showed significant improvement in relapse prevention, but there were no differences between groups. |
| Kortrijk et al. (2010); Netherlands | Observational. Prospective longitudinal study.  Classic ACT / ACT teams (Netherlands) | N = 139 Medical diagnosis: SMI   - Schizophrenia: 61.7% - Other psychosis: 10.6% - Affective disorders: 7.3% - Substance abuse: 51.7%   Age: Mean = 38.3 years, SD = 9.5 Sex: 82.7% men  Groups: N/A | - Aged ≥18 years - Diagnosis of SMI (mainly psychosis or bipolar disorder, with or without addiction) - Lack of motivation for treatment (need for intensive intervention) | Urban | January 2003 - August 2008 | - HoNOS - Motivation for treatment (Likert-type scale adapted from SPI) - CAN scale: alcohol and drug items - DSM-IV diagnosis | Significant improvement in HoNOS scores was observed over time, especially in the initial months. Predictors of worse outcomes included substance abuse, age over 30, low educational level, and low motivation for treatment. Sex differences were noted: in men, worse outcomes were linked to age, low education, and substance abuse; in women, substance abuse and low motivation had a more pronounced negative impact. |
| Kortrijk et al. (2012); Netherlands | Observational. Cross-sectional study.  Classic ACT / ACT teams (Netherlands) | N = 278 Medical diagnosis: Schizophrenia or other psychotic disorder Age: Mean = 41.4 years, SD = 10.8 Sex: 82.4% men  Groups: N/A | - Aged ≥18 years - Diagnosis of severe psychotic disorder - Difficulty adhering to conventional treatment, need for intensive community care - Treated by one of the 7 ACT teams in Rotterdam - At least two consecutive complete evaluations using PANSS and HoNOS | Urban | N.D. | - PANSS - HoNOS - Quality of life (QoL): abbreviated version of MANSA | Among patients, symptomatic remission (SR) and functional remission (FR) occurred in distinct proportions, with some achieving both and others none. SR and FR were independent; achieving one was not required to achieve the other, though the presence of SR increased the odds of FR. Use of antipsychotics predicted both remissions, while comorbidity with substance use disorder negatively impacted SR. Functional remission, with or without symptomatic remission, was associated with higher quality of life, while symptomatic remission alone was not. In multivariate analysis, only functional remission independently predicted higher quality of life. |
| Kortrijk et al. (2014); Netherlands | Observational. Retrospective cohort study.  ACT (Dutch implementation; moderate fidelity, DACTS mean 3.5) | N = 251 Medical diagnosis: SMI Psychotic disorder: 62% Age: Mean = 40 years Sex: 73% men  Groups: N/A | - Aged ≥18 years - Diagnosis of SMI, usually psychosis or bipolar disorder - Low initiamotivation for treatment (need for assertive intervention) | Urban | February 2002 - April 2012 | - Subjective quality of life (6-item scale similar to MANSA, score 1-7 per item) - CANSAS (22-item needs assessment: met, unmet, or not needed) - Change in number of unmet needs - Follow-up duration - Motivation for treatment variable | There was a significant increase in quality of life, accompanied by a reduction in the number of unmet needs. About 40% of participants showed clinically significant improvement in quality of life, nearly half remained stable, and a smaller portion worsened. Needs least associated with improvements in quality of life included housing, daytime activities, self-care, psychotic symptoms, companionship, and finances. The overall association between resolution of individual needs and quality of life was weak. |
| Kortrijk et al. (2019); Netherlands | Observational. Prospective longitudinal cohort study.  ACT-model classification: Flexible ACT (Dutch FACT model) | N = 2660 Medical diagnosis: SMI Age: Mean = 43.2 years, SD = 10.8 Sex: 59.7% men  Groups: N/A | - Aged ≥18 years - Diagnosis of SMI: psychiatric history or ≥2 years of treatment plus functional disability - Continuous contact with a FACT team for 2 years (minimum 3 ROM evaluations, one per year) | Urban | January 2011 – July 2016 | - Severity of psychosocial problems (HoNOS) - Unmet needs (CANSAS, 27-item version) - Quality of life (QoL, 7-item scale) | The majority of patients remained stable in their level of severity. Unmet needs decreased in most groups. Patients who experienced deterioration showed an increase in unmet needs. Substance use problems, particularly alcohol, predicted worsening outcomes. The establishment of specialized FACT teams for personality disorders or substance use is recommended. |
| Kreindler et al. (2010); Canada | Observational. Retrospective longitudinal study  Assertive Community Treatment (ACT) — high-fidelity ACT team (PACT Hargrave, Winnipeg, Canada) | N = 65 Medical diagnosis: SMI Age: Mean = 37.8 years, SD = 11.6 Sex: 63.1% male  Groups: N/A | - Active ACT clients with at least 1 year of participation - Data collected retrospectively from 2 years before to 4–7 years after ACT entry | Urban | June 2001 - February 2008 | - Housing stability (frequency of moves and duration of residence) - Type of housing (independent or non-independent) - Presence of rent subsidy - Neighborhood income level - Substance abuse | Six months after entering ACT, clients showed sustained improvements in housing stability. The proportion of participants living in independent housing increased. Rent subsidies, independent housing, and neighborhoods with higher socioeconomic status were linked to greater housing stability. Women, younger individuals, and those with substance abuse issues were at higher risk of housing instability. ACT played a key role in facilitating access to and maintenance of independent housing. |
| Lafave et al. (1996); Canada | RCT  Assertive Community Rehabilitation Program (ACT-based; Stein & Test model) — Canadian ACT experience (Brockville Psychiatric Hospital, Ontario). | N = 65 Medical diagnosis: SMI Age: Mean = 35.8 years, SD = 2 Sex: Not specified  Groups:   - N intervention group (ACR program) = 24 - N control group = 41 | - Aged between 16 and 65 years. - Diagnosis: SMI (including personality disorders). - Intensive use of psychiatric services: high utilization of mental health resources and need for psychiatric rehabilitation. - Referred to Brockville Psychiatric Hospital for psychiatric rehabilitation between July 1990 and June 1991. - No exclusion based on symptom severity. | N.D. | July 1990 – June 1991. Follow-up: 12 months after treatment assignment | - Days of hospitalization during follow-up - Number of hospitalizations during the follow-up year - Proportion of patients living in the community each month (measured from October 1990 to October 1992) - Quality of Life Interview - Client Satisfaction Questionnaire - Environmental Index - Brief Psychiatric Rating Scale (BPRS) | The ACT group experienced significantly fewer hospital days and hospitalizations compared to the control group. A higher proportion of ACT patients lived in the community, and they demonstrated better objective quality of life, particularly in housing-related aspects. Housing autonomy was greater in the ACT group. Both groups reported moderate satisfaction with services, though ACT participants were more likely to recommend the program. |
| Lambert et al. (2010); Germany | Quasi-experimental study with non-randomized control group  High‑fidelity Assertive Community Treatment (ACT) integrated within a specialized psychosis care program (ACTA‑guided; DACTS = 4.5). | N = 120 Medical diagnosis: Schizophrenia spectrum disorders Age:   - ACT group mean = 31.4 years, SD = 9.9 - Standard Care group mean = 37.6 years, SD = 11.7   Sex:   - ACT group = 56.3% male - Standard Care group = 57.1% male   Groups:   - N intervention group (ACT) = 64 - N control group = 56 | - Diagnosis of schizophrenia, schizoaffective disorder, delusional disorder, or psychotic disorder not otherwise specified (per DSM-IV). - Current or recent treatment with immediate-release quetiapine. - For patients with multiple episodes: at least one psychotic relapse requiring hospitalization due to nonadherence in the past 24 months. | Urban | April 2005 - December 2008 | - Time to service disengagement - Treatment non-adherence (≥1 week without medication) - Symptom improvement (PANSS, CGI-S) - Level of functioning (GAF, Modified Vocational Status Index) - Quality of life (Q-LES-Q-18) - Satisfaction with care (CSQ-8 for patients and family members) - Service use data (hospitalizations, outpatient visits) | Fewer patients disengaged from services in the ACT group compared to the control group. Service retention was longer in ACT participants. Hospitalizations were less frequent in the ACT group. ACT patients also had better outcomes in symptoms, functioning, quality of life, and satisfaction. Medication non-adherence was significantly lower in the ACT group. |
| Lamberti et al. (2017); USA | RCT  Forensic Assertive Community Treatment (FACT) — standardized FACT model with high‑fidelity ACT core (DACTS = 4.75) plus criminogenic‑risk targeting, legal leverage, and mental‑health/justice collaboration (FACTS‑rated). | N = 70 Medical diagnosis: Psychotic disorder   - Schizophrenia = 51% - Depression with psychotic features = 19%, - Schizoaffective disorder = 11%, - Psychotic disorder NOS = 10% - Bipolar disorder with psychotic features = 9%   Age: Mean = 37.5 years, SD = 11.6 Sex: 61% male  Groups:   - N intervention group (FACT) = 35 - N control group = 35 | - Age: 18 years or older. - Diagnosis of a psychotic disorder according to DSM-IV-TR. - Capacity to consent to participation in the research. - History of conviction for a minor offence and eligibility for conditional release. - Not currently under probation, mental health court supervision, or assisted outpatient treatment at enrolment. - Exclusion of individuals with pending charges for serious crimes (but participants with previous convictions for serious crimes were allowed). | Urban | February 2011 – May 2014. One-year follow-up; last patient follow-up completed in May 2015 | - Days of psychiatric hospitalization during the follow-up year - Number of new criminal convictions during the study - Number of days in jail after the intervention - Days in outpatient mental health care - Number of psychiatric emergency visits - Number of arrests during follow-up - Number of parole revocations by a judge - Number of outpatient mental health service contacts - Level of psychiatric medication adherence - Severity of psychiatric symptoms - Insight into illness - Substance abuse evaluation - Risk level for criminal recidivism | The FACT group had fewer criminal convictions and jail days compared to the control group, with significant reductions in both. Psychiatric hospitalization days were also significantly lower in the FACT group. FACT participants spent more time in outpatient treatment. No significant differences were observed between groups in the number of arrests, psychiatric emergency visits, or total incarcerations, though the FACT group had more incarcerations related to noncompliance with judicial conditions rather than new offenses. |
| Lang et al. (1999); USA | Observational. Retrospective cohort study.  Classic Assertive Community Treatment (Stein & Test–derived ACT model) | N = 45 Medical diagnosis: SMI   - Schizophrenia or schizoaffective disorder: 88.9% - Affective disorder with psychotic features: 11.1%   Age: Mean = 41.6 years, SD = 9.4 Sex: 69% men  Groups:   - N Participants who received ACT services since program inception = 81 - N Participants currently in the program at time of survey = 61 - N Participants who participated in the survey = 45 | - Patients treated by the ACT team at the Connecticut Mental Health Center (New Haven) between 1992 and 1997 - History of SMI with multiple needs: homelessness, substance use, legal history, nonadherence, social isolation | Urban | 1992 - December 1997 | - Psychiatric symptoms - Substance abuse - Medical problems - Medication adherence - Primary supports - Social supports - Occupation/vocation - Qualitative assessment of income reasons - Housing - Daily living skills - Economic issues - Legal involvement - Behavioral problems - Treatment involvement - Number of hospitalizations and jail admissions (before and after) | There was a significant reduction in institutional admissions, including hospitals and jails, during the first two years of the program, with the reduction maintained through year five. Some patients alternated between hospitalization and jail. Both clients and clinicians reported significant improvement across multiple areas. Predictors of reduced institutionalization included clinical improvement in substance abuse problems and patient-reported improvements in social support and economic status. |
| Latimer et al. (2020); Canada | RCT (cost-effectiveness analysis)  Housing First (scattered‑site Pathways model) with Assertive Community Treatment (HF+ACT) | N = 950 Medical diagnosis: Mental disorder (psychotic disorder or bipolar disorder, with frequent substance comorbidity) Age distribution: <30 years: 24%, 30–49 years: 60%, ≥50 years: 17% Sex: 68.31% men  Groups:   - N Intervention group (Housing First + ACT) (n = 469), 68% men - Control group (TAU) (n = 481), 68% men | Aged ≥18 years, diagnosed mental disorder, absolute homelessness or residential instability with a history of homelessness. | Urban | October 2009 – June 2011 (follow-up of up to 24 months) | - Days of stable housing (main variable) - Use of services: psychiatric and physical hospitalizations, emergency room visits, incarcerations, shelters, outpatient visits - Total annual cost per participant | There was a substantial average increase in days of stable housing. A significant portion of the intervention cost was offset by reductions in other services. The incremental cost per additional day of stable housing was modest. The total annual cost of the intervention and the net cost per person were reasonable. |
| Lee et al. (2015); Hong Kong | Quasi-experimental study with historical controls.  Recovery‑oriented Assertive Community Treatment (ACT) | N = 210 Medical diagnosis: Psychotic disorders (74–79%) Age:   - ACT group: Mean = 40.34 years, SD = 11.74 - Control 1 group: Mean = 38.96 years, SD = 11.55 - Control 2 group: Mean = 41.81 years, SD = 11.73   Sex:   - ACT group: 55.7% men - Control 1 group: 54.3% men - Control 2 group: 59% men   Groups:   - N Intervention group (ACT) = 70 - N Control 1 group = 70 - N Control 2 group = 70 | - Three or more psychiatric hospital admissions in the 12 months prior to selection - Aged 18–65 years - Exclusion: intellectual disability, dementia, or sole diagnosis of substance abuse | Urban | September 2008 - July 2010 (intervention) + follow-up up to 18 months.  Control period C1: August 2006 – February 2008, Control period C2: May 2010 – April 2012 | - Number of psychiatric readmissions (ReAdm) - Days of hospitalization (LOS) - Emergency room attendance (AED) - NoFUdays - BPRS (Brief Psychiatric Rating Scale) - WHOQOL-BREF-HK (quality of life in 4 dimensions: physical, psychological, social relations and environment). | All groups showed improvement due to deinstitutionalization, but the ACT group demonstrated greater reductions in readmissions and hospital days, fewer missed appointments, significant improvement in symptoms, and moderate improvement in quality of life, especially in social relationships. |
| Lee et al. (2015); Hong Kong | Observational. Historical cohort study.  High‑intensity Assertive Community Treatment (ACT) | N = 199 Medical diagnosis: SMI Age:   - ACT group: Mean = 40.34 years, SD = 11.74 - Control 1 group: Mean = 38.96 years, SD = 11.55 - Control 2 group: Mean = 41.90 years, SD = 11.62   Sex:   - ACT group: 55.7% men - Control 1 group: 54.3% men - Control 2 group: 55.9% men   Groups:   - N ACT = 70 - N Control 1 = 70 - N Control 2 = 59 | - Aged 18–65 years - ≥3 psychiatric hospitalizations between April 2007 and December 2008 - Exclusions: under 18 or over 65, primary diagnosis of intellectual disability, dementia, or substance use disorder without another major psychiatric condition | Urban | May 2010 – January 2012 | - Brief Psychiatric Rating Scale (BPRS). - Specific Level of Functioning Scale - WHOQOL-BREF (Quality of Life: Physical, Psychological, Social, Environmental) - No. of hospital readmissions - Days of admission - Psychiatric emergency room attendances - No. and duration of dropouts from follow-up - No. of unplanned readmissions - No. of home visits | Significant improvements were observed in the ACT group compared to controls, including reductions in hospital readmissions, hospital stays, and unplanned readmissions. Loss to follow-up decreased in both frequency and duration. Contact with professionals, especially home visits, increased. Functioning improved as measured by specific scales, psychiatric symptoms decreased, and quality of life increased in physical, psychological, and environmental dimensions. No significant differences were found in social relationships. |
| Lehman et al. (1997); USA | RCT  High‑fidelity Assertive Community Treatment (ACT) | N = 152 Medical diagnosis: SMI Age:   - ACT group: Mean = 39 years, SD = 9.5 - Control group: Mean = 36 years, SD = 8.3   Sex:   - ACT group: 65% male - Control group: 70% male   Groups:   - N Intervention group (ACT) = 77 - N Control group = 75 | - Diagnosis of severe and persistent mental illness according to DSM-III-R. - Extensive psychiatric hospitalization history, defined as: - At least 2 psychiatric hospitalizations longer than 21 days in the last 3 years, or - A total of 42 or more days in a psychiatric hospital in the past year, or - More than 90 days of hospitalization over the past 3 years. - Severe functional impairment, defined as the inability to maintain employment, education, or homemaking for at least 75% of the past year. - Homeless or sheltered living for: - At least 5 of the past 45 days, or - At least 14 of the past 180 days, or - Having changed residence at least twice in the last 6 months. | Urban | March 1991 – September 1992. Follow-up: 12 months after group assignment | - Psychiatric hospital use during the 12 months after treatment allocation - Total number of days hospitalized - Number of emergency room visits - Number of outpatient mental health consultations - Days in stable housing - Frequency of incarcerations - Psychiatric symptoms: Colorado Symptom Index (CSI) - Quality of life: Lehman Quality-of-Life Interview (QOLI) - Self-perceived health: Medical Outcomes Study 36-Item Short Form Health Survey (SF-36) | The ACT group had fewer psychiatric hospitalization days and emergency visits compared to standard care. They also had more outpatient mental health consultations and spent more days in stable housing. Significant improvements in psychiatric symptoms were observed in the ACT group, along with higher life and health satisfaction compared to the control group. |
| Lehman et al. (1999); USA | RCT(cost-effectiveness analysis)  High‑fidelity Assertive Community Treatment (ACT) | N = 152 Medical diagnosis: SMI   - Schizophrenia or schizoaffective disorder: 58% - Bipolar disorder: 20% - Major depression: 8% - Other SMI: 14%   Age: Mean = 37 years Sex: 67% men  Groups:   - N Intervention group (ACT) = 76 - N Control group = 76 | - Diagnosis of severe and persistent mental illness (based on duration, diagnosis, and disability) - Literally homeless (on the street or in shelters) - Informed consent | Urban | March 1991 – September 1992 | - Days in stable housing - Direct costs: outpatient visits (mental health and medical), inpatient (mental health and medical), emergencies, substance use treatment, outpatient and inpatient rehabilitation - Days in jail (reported in previous 1997 publication) - Quality of life and clinical outcomes. | The ACT group spent more days in stable housing than the control group and had a lower cost per day in stable housing, resulting in a favorable efficiency ratio. ACT reduced psychiatric hospitalizations and emergency room visits while increasing outpatient visits and substance abuse treatment. There was a trend toward lower total costs with ACT, though it was not statistically significant. Efficiency was higher in white patients compared to African American patients. |
| Liem et al. (2013); Hong Kong | Quasi-experimental study with historical control group.  Modified Assertive Community Treatment (ACT) | N = 140 Medical diagnosis: Schizophrenia, mood or anxiety disorder Age: ACT group mean = 40.3 years, SD = 11.7 Sex: ACT group = 55.7% male  Groups:   - N intervention group (ACT) = 70 - N control group = 70 | - Aged between 18 and 65 years. - Diagnosis of schizophrenia, mood disorder, or anxiety disorders. - Three or more psychiatric hospitalizations at Kwai Chung Hospital in the previous 12 months. | Urban | Intervention group: 2007–2008. Control group: 2005–2006 | - Number of psychiatric readmissions - Days of hospitalization - Emergency room visits - Number of missed medical appointments | The ACT group showed a significant reduction in psychiatric readmissions compared to the control group. They also had fewer hospitalization days and missed fewer medical appointments. However, the control group experienced fewer emergency room visits at 24 months |
| López-Santiago et al. (2012); Spain | Observational. Retrospective pre-post study.  Assertive Community Treatment (ACT)–inspired program (“PTAC‑Albacete”) | N = 55 Medical diagnosis: SMI   - Schizophrenia: 60% - Schizoaffective disorder: 21.8% - Bipolar disorder: 16.4% - Borderline personality disorder: 1.8%   Age: Mean = 41.94 years, SD = 12 Sex: 54.5% men  Groups: N/A | - Diagnosis of SMI - Minimum duration in the program: - ≥12 months for the first analysis - ≥36 months for the second analysis | Urban | 2002-2008 | - Number of hospital admissions - Days of hospitalization - Psychiatric emergencies - Global Activity Assessment score (EEAG) | At 12 months, there were substantial reductions in admissions, hospitalization days, and emergency visits, with moderate to large effect sizes. At 36 months, these reductions were maintained, with continued decreases in admissions, hospitalization days, and emergency visits, accompanied by strong effect sizes. |
| Low et al. (2013); Singapore | Observational. Retrospective cohort study  Assertive Community Management (ACM) — ACT‑derived model | N = 155 Medical diagnosis: SMI Age: Mean = 44.3 years, SD = 10.6 Sex: 50.3% men  Groups: N/A | - Aged 18–65 years - Severe and persistent mental illness (e.g., schizophrenia, delusional disorder, bipolar disorder) - Presence of severe symptoms and impairments causing distress and significant disability in daily functioning - Significant disability caused by SMI that does not improve with traditional outpatient services - Patients with a total of 3 admissions and/or hospitalization lasting more than 30 days in the past year | Urban | N.D. | - Number of hospital admissions (NOA). - Length of Stay (LOS). | There was a substantial average reduction in the number of admissions and length of hospital stay. The ACM model showed significant effects on the number of admissions. A significant interaction between diagnosis and sex was found, with men diagnosed with schizoaffective disorder experiencing more admissions than men with schizophrenia. |
| Luo et al. (2019); China | RCT  Culturally adapted high‑fidelity Assertive Community Treatment (ACT) | N = 60 Medical diagnosis: Schizophrenia Age:   - ACT group mean = 29.2 years, SD = 4.3 - Control group = 30.2 years, SD = 7   Sex:   - ACT group = 70% male - Control group = 66.7% male   Groups:   - N intervention group (ACT) = 30 - N control group = 30 | - Age between 18 and 45 years. - Diagnosis of schizophrenia based on DSM-IV. - No organic brain disorders, intellectual disability, severe traumatic brain injury or personality disorder. - Significant functional impairment. - Need for high-level mental health services (based on ACT manual criteria). - Not living with another family member with mental illness. - Informed consent obtained from the patient and their cohabiting family caregiver. | Urban | April 2013 – July 2013. Follow-up: 12 months | - Days of hospitalization - Days in relapse (defined by PANSS criteria + hospitalization + clinical behavior) - Days in paid employment over 12 months - PANSS (Positive and Negative Syndrome Scale) - Global illness severity (Clinical Global Impression – CGI) - Social functioning (Personal and Social Performance – PSP) - Social disability (Social Disability Screening Schedule – SDSS) - Daily living skills (UCSD Performance-Based Skills Assessment – Brief, UPSA-B) - Family functioning (Family Assessment Device – FAD) - Caregiver quality of life (WHOQOL-BREF) | The ACT group experienced significantly less hospitalization, with fewer rehospitalizations and fewer days spent in the hospital compared to the control group. They also had fewer relapses and fewer days in relapse. Employment rates and days worked were higher in the ACT group. Significant improvements were observed in psychiatric symptoms and social functioning. Additionally, caregivers reported better quality of life and improved family relationships. |
| Mantzouranis et al. (2019); Switzerland | Observational. Retrospective cohort study.  Adolescent Assertive Community Treatment (youth‑ACT) | N = 179 Medical diagnosis: SMI   - Depression: 34.64% - Anxiety disorders: 18.44% - Behavioral disorders: 13.97% - Personality disorder: 10.61% - Psychosis: 10.06% - Others without a formal diagnosis (referred by schools, social services): 12.28%   Age: Mean = 15.76 years, SD = 1.76 Sex: 56.98% men  Groups: N/A | All the adolescents followed by the ACT teams in the Vaud canton. | Mixed | 2010-2014 | - Reliable Change Index (RCI), - HoNOSCA( HoNOSCA total, HoNOSCA-Ext, HoNOSCA-Emo), - GAF (Global Assessment of Functioning). | Statistically significant improvements were observed in total HoNOSCA scores, emotional and externalizing subscales, and global functioning (GAF). Clinically significant improvements were noted in a portion of patients across various domains, including emotional difficulties, externalizing behavior problems, and global functioning. The rate of clinical deterioration was low. |
| Manuel et al. (2011); USA | RCT  Assertive Community Treatment (ACT) — standard ACT delivering integrated dual‑diagnosis treatment (high‑intensity ACT vs. standard clinical case management). | N = 198 Medical diagnosis: Psychosis Age: Not determined Sex: Not determined  Groups:   - N Intervention group (ACT) = 99 - N Control group (SCCM) = 99 | - Diagnosis of psychosis (schizophrenia, schizoaffective disorder, or other). - Active substance use (abuse or dependence). - High utilization of mental health services in the past 2 years, including at least two of the following: - Psychiatric hospitalizations - Crisis program stays - Psychiatric emergency room visits - Incarceration - Homelessness or unstable housing - No pending legal charges or severe medical conditions. - Informed consent obtained. | Urban | August 1993 – July 1998. Follow-up: 3 years with assessments every 6 months | - Psychiatric medication adherence - Measured via self-report of days without medication in the past 6 months - Classified as: - Poor adherence: ≥20% of days without medication - Good adherence: <20% of days without medication - Use of mental health services - Change in psychiatric symptoms - Substance use | ACT improves medication adherence in individuals with psychosis and substance abuse. Benefits may stem from more intensive follow-up, medication delivery, and continuous monitoring. ACT is especially useful for patients with high complexity and poor adherence. However, adherence was measured only by self-report, which may affect the accuracy of the results. |
| Marquant et al. (2018b); Belgium | Quasi-experimental study with non-randomized control.  Forensic Assertive Community Treatment (ForACT) | N = 140 Medical diagnosis: Mental disorders Age:   - ForACT group: Mean = 39.5 years, SD = 10.1 - Control group: Mean = 39.9 years, SD = 9.8   Sex: 100% men  Groups:   - N Intervention group (ForACT) = 70 - Control group = 56 | Forensic inpatients (male internees); exclusions: women, high-risk individuals, primary diagnosis of paraphilia or intellectual disability | Urban | April 2012 – January 2015 (33-month follow-up) | - Arrests - Re-admissions to prison. - Hospitalizations, length of stay - Presence of antisocial traits, history of violence, dual diagnosis. | ForACT was associated with a lower probability of arrest and imprisonment. Although hospitalization rates were higher in ForACT, hospital stays were shorter. Treatment continuity was better in ForACT. Hospitalizations were primarily related to substance use. |
| McCrone et al. (2009); United Kingdom | RCT (cost-effectiveness analysis)  High‑fidelity Assertive Community Treatment (ACT) | N = 251 Medical diagnosis: SMI Age:   - ACT: Mean = 39 years, SD = 11 - Usual care: Mean = 40 years, SD = 11   Sex:   - ACT: 62% men - Usual care: 55% men   Groups:   - Intervention group (IC-TACT) = 127 - Control group (Usual Care) = 124 | - Referral from community mental health teams (CMHT) - Low engagement with services - Primary diagnosis of SMI (e.g., schizophrenia, schizoaffective, bipolar or other psychotic disorder) - Recent intensive use of hospitalization: - ≥100 consecutive days or ≥5 admissions in the last 2 years - or ≥50 consecutive days or ≥3 admissions in the past year - or clinical judgement indicating significant risk to self or others | Urban | July 1999 and July 2002 | - Total cumulative cost at 18 months (mental health services, hospitalization, community care, etc.). - Costs by type of resource used (inpatient, outpatient, home visits, etc.). - Associated cost per unit of improvement in satisfaction - Comparison of costs between groups (ACT vs. usual care). - Psychiatric symptoms - Global functioning - Satisfaction variables: Level of user satisfaction with services received - Engagement with services: level of involvement/engagement with services. | No significant differences in costs were found between ACT treatment and usual care. While ACT did not generate economic savings during the study period, it resulted in greater user satisfaction and higher levels of engagement with services. |
| McFarlane et al. (1996); USA | RCT  Assertive Community Treatment (ACT) with two levels of structured family involvement (ACT + Psychoeducational Multifamily Group Treatment/ACT + Crisis‑Only Family Intervention) | N = 68 Medical diagnosis: Schizophrenia or schizophrenia spectrum disorders Age: Mean = 29.8 years, SD = 5.8 Sex: 64.7% male  Groups:   - Multifamily group = 37 (70.3% male) - Crisis family intervention group = 31 (58.1% male) | - Diagnosis of schizophrenia or schizophrenia spectrum disorders. - At least one complicating factor such as: - Non-adherence to treatment - History of violence or suicidality - Multiple hospitalizations or frequent emergency use - Criminal history - Unstable housing - Moderate to severe substance abuse - At least one relative willing to participate in the intervention. | Mixed | 1987 – early 1991. Follow-up: 24 months after group assignment | - Number of hospitalizations in each 6-month period - Positive and Negative Syndrome Scale (PANSS) - Medication Compliance Scale (6 levels) - Family burden and well-being: Social Adjustment Scale-III (SAS-III), family version - Employment and social functioning: Evaluation of competitive vs. supported employment | Both groups experienced reductions in psychiatric symptoms and hospitalizations. The Multifamily Group achieved better employment outcomes, including a higher employment rate and greater participation in supported employment. No significant differences were found between groups in hospitalization or symptom levels. Both groups showed improvements in family burden and well-being. |
| McGrew et al. (1995); USA | Observational. Prospective longitudinal study.  Assertive Community Treatment (ACT) | N = 212 Medical diagnosis: SMI   - Schizophrenia or schizoaffective disorder: 51.8% - Schizoaffective disorder: 13.2% - Affective disorder: 17.5% - Anxiety disorder: 1.9% - Personality disorder: 8.0% - Substance use disorder (primary): 0.5%   Age: Mean = 36.1 years, SD = 10.3 Sex: 55.2% men  Groups: N/A | - Aged ≥18 years - Chronic SMI (Indiana Department of Mental Health criteria) - Inappropriate users or non-responders to existing services - Significant risk of rehospitalization (≥3 hospitalizations in the past 2 years and ≥5 lifetime hospitalizations) | Mixed | Program start: April 1989. Data from 2 years prior and 18-month follow-up. | - Number of admissions and days hospitalized. - Global functioning: GAF and SBLOF - Quality of life: LSC scale (client and professional) - Other: employment, income, housing, legal problems, medication compliance, perception about medications | Over the 18-month follow-up, hospital admissions and length of stay progressively decreased. Quality of life and overall functioning improved according to both clients and professionals. No changes were observed in employment, treatment adherence, or attitudes. Financial management improved, while legal issues increased, possibly due to enhanced monitoring. |
| McGuire et al. (2004); USA | Observational. Prospective cohort study.  Assertive Community Treatment (ACT) case‑management model | N = 5774 Medical diagnosis: SMI   - Schizophrenia: 37% - Other psychotic disorder: 31% - Major depression: 49% - Bipolar disorder: 20% - Personality disorder: 22% Age: Mean = 39 years, SD = 9.56 Sex: 61% men   Groups: N/A | - Diagnosis of SMI - Homeless situation: living in a shelter, abandoned building or outdoors for at least 7 of the 14 days prior to admission | Mixed | May 1994 – June 1998: 12-month follow-up | - Psychiatric problems (composite psychiatric score – Addiction Severity Index) - Alcohol and drug use problems - Days homeless (past 60 days) - Days employed (past 30 days) - Days in jail (past 60 days) - Healthcare and criminal justice system costs - Instrumental social support - Childhood abuse - Childhood conduct disorder - Family-of-origin stability | Individuals with a history of incarceration for six months or more showed greater clinical and social complexity, including higher psychiatric and addiction severity, more time spent homeless, and increased rates of dual diagnoses and jail days at follow-up. Despite these challenges, differences in service use and total costs were minimal. All groups demonstrated improvements across multiple indicators after 12 months, with the most notable gains observed in psychiatric symptoms and reductions in criminal recidivism. |
| Meisler et al. (1997); USA | Observational. Retrospective cohort study.  Integrated Assertive Community Treatment (ACT/TCL hybrid)for homeless adults with co‑occurring severe mental illness and substance use disorders | N = 114 Medical diagnosis: SMI Age: Mean = 39 years (range: 19–61) Sex: 49.1% men  Groups: N/A | Individuals with severe mental illness who were homeless or about to be discharged from hospital or prison with no residence to return | Urban | 1988-1992 | - Severity of Substance Use (Case Manager Rating Scale) - Housing Situation - Employment Psychiatric - Admissions and Hospitalization Days - Arrests and Days in Prison | High retention and ongoing engagement were observed, along with reductions in hospitalization and homelessness. The homeless population achieved an outstanding employment rate. Better outcomes were found among individuals who attained abstinence or mild control over substance use. The severity of substance use was the main negative predictor of outcomes, rather than the duration in the program. |
| Mohamed (2016); USA | Observational. Prospective cohort study.  ACT‑like Intensive Case Management (ICM) — VA MHICM program — nationwide Veterans Health Administration Mental Health Intensive Case Management (MHICM) | N = 5660 Medical diagnosis: Psychiatric diagnoses Age:   - Nonpsychotic: Mean = 53.2 years, SD = 13.4 - Psychotic, no symptoms: Mean = 51.8 years, SD = 11. - Psychotic with symptoms: Mean = 52.1 years, SD = 11.7   Sex:   - Nonpsychotic: 83.8% men - Psychotic, no symptoms: 87.2% men - Psychotic with symptoms: 88.6% men   Groups:   - N Nonpsychotic group = 820 - Psychotic no symptoms = 2338 - Diagnosis and symptom = 2502 | Participation in the VA ACT-like program (MHICM) | Urban | N.D. | - Days working - Days consuming alcohol and drugs - Quality of life - GAF (Global Assessment of Functioning) - BSI (Brief Symptom Inventory) - BPRS (Brief Psychiatric Rating Scale) - Instrumental activities of daily living - Violence and suicidal behavior - Satisfaction with service - Therapeutic alliance | All groups combined showed significant improvements in psychiatric symptoms, alcohol use, global functioning, income, and satisfaction with services. However, no significant changes were observed in employment days or drug use. Participants with moderate psychotic symptoms showed less improvement in psychological distress and alcohol use compared to the other groups. |
| Mohamed et al. (2010); USA | Observational. Retrospective cohort study.  ACT‑like Intensive Case Management (ICM) — VA MHICM program — nationwide Veterans Health Administration Mental Health Intensive Case Management (MHICM) | N = 1402 Medical diagnosis: SMI Age:   - Early termination: Mean = 50.0 years, SD = 10.6 - Later termination: Mean = 51.0 years, SD = 11.3 - Nontermination: Mean = 50.52 years, SD = 9.7   Sex:   - Early termination: 93% men - Later termination: 91% men - Nontermination: 90% men   Groups:   - N Early termination group = 230 - N Later termination group = 371 - N Nontermination group = 801 | - Veterans with severe and persistent mental illness. - Presence of significant functional impairment. - Inadequately served by standard outpatient care. - History of high levels of psychiatric hospitalization. | Urban | N.D. | - Number of hospitalizations - Length of hospital stay (days) - Overall clinical improvement - Treatment cost | There was a significant reduction in psychiatric hospitalization days following enrollment in ACT, along with fewer admissions compared to the year prior. Total treatment costs decreased after implementing ACT, although a detailed cost-effectiveness analysis was not conducted. ACT proved to be clinically effective and may offer a more cost-efficient alternative to traditional inpatient care. |
| Morrissey et al. (2013); USA | Quasi-experimental study with matched control group.  Recovery‑oriented Assertive Community Treatment (ACT) | N = 900 Medical diagnosis: Schizophrenia and affective disorders Age:   - PACT group mean = 41 years, SD = 11.8; - Control group mean = 40 years, SD = 11.7   Sex:   - PACT group = 62% male; - Control group = 60% male   Groups:   - N intervention group (PACT) = 450 - N control group = 450 | Schizophrenia and affective disorders in patients with high levels of psychiatric hospitalization. | Mixed | PACT: admissions from July 2007. Control group: January 2001 -June 2007 | - Number of state psychiatric hospitalization days per year - Associated hospital costs (inflation-adjusted to 2010 USD) - Use of local psychiatric hospitals (days) - Emergency room visits (number of visits) - Use of crisis stabilization units (days) - Number of arrests | PACT led to a significant reduction in the number of state psychiatric hospitalization days per person per year, along with substantial decreases in annual per-patient costs. While the use of local psychiatric hospitals and crisis stabilization units increased, there was no significant reduction in the likelihood of state hospitalization, only in the duration. The greatest impact was observed among individuals with the highest number of prior hospitalization days. |
| Morse et al. (1997); USA | RCT  Assertive Community Treatment (ACT) and ACT + Community Workers | N = 165 Medical diagnosis: SMI   - Schizophrenia = 66% - Recurrent depression = 15% - Bipolar disorder = 13% - Atypical psychosis = 12% - Delusional or paranoid disorder = 3% - Dementia = 1.2%   Age: Mean = 34.76 years, SD = 10.41 Sex: 58% male  Groups:   - N Assertive Community Treatment with community workers group = 55 - N Assertive Community Treatment only group I= 55 - N Broker case management group = 55 | - Homeless or at risk of homelessness. - DSM-III-R Axis I diagnosis. - Willingness to receive support with welfare, employment, housing, mental health, or other social services. - No serious criminal offences in the past year (e.g., homicide, sexual assault, aggravated assault, arson). | Urban | 1990 -1993 | - Contacts with the assigned program - Access to resources (housing, employment, social benefits) - Number of days in stable housing - Psychiatric symptoms: Brief Psychiatric Rating Scale (BPRS) - Self-esteem: Rosenberg Self-Esteem Scale - Substance abuse measures: Addiction Severity Index (ASI) | Assertive Community Treatment proved more effective than brokered case management in increasing the number of days in stable housing, improving patient satisfaction, reducing psychiatric symptoms (particularly thought disorders and unusual activity) and enhancing access to resources such as housing and financial assistance. However, no significant differences were found between the groups in terms of income, self-esteem, or substance use. |
| Mowbray et al. (1997); USA | RCT  Assertive Community Treatment (ACT) — first full replication of the original PACT model (Training in Community Living), Harbinger Program (Kent County, Michigan). | N = 121 Medical diagnosis: SMI Age distribution:   - Harbinger group: 18–27 = 36%, 28–37 = 42%, 38–47 = 10%, ≥48 = 12% - Control group: 18–27 = 42%, 28–37 = 32%, 38–47 = 11%, ≥48 = 15%   Sex:   - Harbinger group = 51% male; - Control group = 50% male   Groups:   - N intervention group (Harbinger) = 59 - N control group = 62 | Patients who presented for admission to the psychiatric unit at Kent Oaks Community Hospital during a period of approximately two years and were considered eligible for an alternative referral to hospitalization. | Urban | Assignment and initial follow-up: 1979-1981. Assessments at 30 and 66 months | - Days of hospitalization during follow-up (primary variable) - Number of hospital admissions - Global Assessment Scale (GAS) - Satisfaction with services - Living and employment conditions - Psychiatric symptoms (self-rated on a 1–10 scale) | At 30 months, the ACT group showed significantly fewer hospital days and hospitalizations, greater residential autonomy, lower unemployment, and better functional status compared to the control group.  At 66 months, group differences were reduced, but ACT participants continued to use fewer hospital services. They also reported higher satisfaction with care and fewer self-reported symptoms. |
| Nielsen et al. (2021); Denmark | Quasi-experimental propensity score–matched controlled study.  Flexible Assertive Community Treatment (FACT) compared with Assertive Community Treatment (ACT) and Community Mental Health Teams (CMHTs). | N = 2034 Medical diagnosis: SMI Age:   - CMHT-FACT group: Median = 44 (IQR 33–54) - CMHT control group: Median = 44 (IQR 33–54) - ACT-FACT group: Median = 44 (IQR 35–54) - ACT control group: Median = 43 (IQR 31–53)   Sex:   - CMHT-FACT group: 55% male - CMHT control group: 55% male - ACT-FACT group: 49% male - ACT control group: 49% male   Groups:   - N CMHT-FACT group = 887 - N CMHT control group = 887 - N ACT-FACT - group = 130 - N ACT control group = 130 | Patients receiving care from CMHT, ACT, or FACT teams in the Capital Region of Denmark as of 1 May 2016 | Mixed | November 2016 - November 2018 | - Number of psychiatric admissions - Number of psychiatric hospitalization days - Number of outpatient contacts (office visits, home visits, network meetings, group therapy, family meetings) - Use of coercive measures (involuntary admission, detention, restraint, or forced treatment) - Episodes of self-harm - Mortality | FACT was associated with fewer psychiatric admissions compared to both CMHT and ACT models. However, no significant differences were found in the total duration of hospitalization. FACT teams had more outpatient contacts and conducted more home visits than standard CMHT or ACT teams. There were no significant differences between groups in the use of coercive measures, self-harm episodes, or mortality rates. |
| Nielsen et al. (2023); Denmark | Quasi-experimental study with non-randomized control group  Flexible Assertive Community Treatment (FACT) vs. Assertive Community Treatment (ACT) | N = 131 Medical diagnosis: SMI Age:   - FACT group: Mean = 48 years, SD = 12.2 - ACT group: Mean = 50 years, SD = 13.3   Sex:   - FACT group = 53% male; - ACT group = 61% male   Groups:   - N FACT group = 74   N ACT group = 57 | Severe mental illness, frequent use of psychiatric inpatient services, and difficulty maintaining outpatient treatment | Urban | No exact date reported, but data compared before and after team reconfiguration between 2018 and 2019 | - Personal and social functioning (Personal and Social Performance – PSP) - Therapeutic alliance/bond (Working Alliance Inventory – WAI) - Patient satisfaction (Client Satisfaction Questionnaire – CSQ-8) | The ACT group demonstrated better personal and social functioning than the FACT group. However, no significant differences were observed in therapeutic alliance or patient satisfaction. These findings suggest that the FACT model may be less effective than ACT in enhancing functioning among individuals with severe mental illness. |
| Nieves (2002); USA | Quasi-experimental study with matched control group.  Assertive Community Treatment (ACT) vs. Continuing Day Treatment Program (CDTP). | N = 60 Medical diagnosis: SMI Age:   - ACT group = 38.57 years; - CDTP group = 40.97 years   Sex:   - ACT group = 52.2% male; - CDTP group = 47.8% male   Groups:   - N ACT group = 30 - N CDTP group = 30 | - Diagnosis of chronic mental illness. - At least one prior psychiatric hospitalization (acute or long-term). - History of nonadherence to traditional outpatient treatment. - Difficulty maintaining stable housing and employment. | Urban | N.D. | - Number of psychiatric hospitalizations - Days of hospitalization - Housing stability - Employment status - Progress on personal goals | In this study, ACT was not more effective than traditional outpatient care (CDTP). It did not lead to reductions in hospitalizations or improvements in housing or employment stability. Methodological deviations from the original PACT model may have limited its effectiveness, and the urban setting of the South Bronx, along with constrained resources, likely influenced the outcomes. |
| Nishio et al. (2012); Japan | Uncontrolled prospective pre-post study.  Assertive Community Treatment (ACT) — ACT‑Japan (ACT‑J) model. | N = 41 Medical diagnosis: SMI   - Schizophrenia = 72.1% - Mood disorder = 18.6% - Others = 9.3%   Age: Mean = 35.8 years, SD = 10.5 Sex: 44.2% men  Groups: N/A | - Psychiatric hospitalization at Kohnodai Hospital between May 2003 and April 2004 (excluding acute intoxications or medical complications) - Age 18–59 years - Primary diagnosis of schizophrenia or mood disorder (excluding intellectual disability, dementia, drug/alcohol abuse, and personality disorder) - Resident in Ichikawa, Matsudo, or Funabashi - Use of mental health services in the past 2 years - History of antisocial or socially disruptive behavior in the past 2 years (violence, substance abuse, homelessness, suicide attempts) - Difficulty performing daily activities in the past year - Treating psychiatrist’s consent to participate in ACT - Informed consent from the patient | Urban | May 2003 – April 2004. Follow-up: 1 year after hospital discharge | - Total number of hospitalization days before and after ACT. - Frequency of hospital readmissions. - Number of times the patient required urgent psychiatric care. - BPRS (Brief Psychiatric Rating Scale). - Social functioning: GAF (Global Assessment of Functioning) scale. - Antipsychotic medication dosage: Chlorpromazine equivalents (CP-equivalents). - Quality of life: QOLI (Quality of Life Inventory, short version). - Employment status: Number of participants employed before and after ACT. | ACT led to a significant reduction in hospitalization days and admissions, improved social functioning, and lowered antipsychotic medication dosage. Negative symptoms decreased, while positive symptoms increased. No significant changes were observed in quality of life or emergency service use. A slight increase in employment was noted following the intervention. |
| Nordentoft et al. (2010); Denmark | RCT, 5-year follow-up  Specialized Assertive Community Treatment (OPUS) vs. Standard CMHC Care vs. Hospital‑Based Rehabilitation. | N = 94 Medical diagnosis: Schizophrenia spectrum psychosis (ICD-10) Age:   - Hospital-based rehabilitation: Mean = 24.97 years, SD = 5.06 - OPUS treatment: Mean = 23.83 years, SD = 4.01 - Standard treatment: Mean = 23.24 years, SD = 4.21   Sex:   - Hospital-based rehab = 64.5% male - OPUS = 64.7% male Standard treatment = 69% male   Groups:   - N Hospital-based rehabilitation group = 31 - N OPUS treatment group = 34 - N Standard treatment group = 29 | - Diagnosis of schizophrenia spectrum psychosis according to ICD-10. - No more than 12 weeks of antipsychotic treatment. - Ability to communicate in Danish without an interpreter. - Residence in the study catchment area. - Informed consent. | Urban | March 1998 – December 2000. Follow-up: 5 years after group assignment | - Number of days in psychiatric hospital over 5 years - Number of days in supervised residences - Scale for the Assessment of Positive Symptoms (SAPS) - Scale for the Assessment of Negative Symptoms (SANS) - Global Assessment of Functioning (GAF) | Patients in the OPUS program had significantly fewer hospital days during the first three years compared to those in inpatient rehabilitation. Even after treatment, patients in hospital-based rehabilitation spent more time in institutional settings such as hospitals or supervised housing. By the five-year follow-up, there were no significant differences in psychotic or negative symptoms between groups. However, OPUS participants achieved greater independence than those in other treatment models. |
| Nordentoft et al. (2015); Denmark | RCT  Integrated treatment = ACT‑based early‑intervention model with family involvement and social skills training. | N = 547 Medical diagnosis: First episode of psychotic illness Age:   - Integrated treatment group: Mean = 26.6 years, SD = 6.4 - Standard treatment group: Mean = 26.6 years, SD = 6.3   Sex:   - Integrated treatment group = 58% male - Standard treatment group = 60% male   Groups:   - N intervention group (Integrated treatment) = 275 - N control group (Standard treatment) = 272 | - Diagnosis of schizophrenia, schizotypal disorder, delusional disorder, brief psychotic disorder, schizoaffective disorder, or unspecified psychosis. - No more than 12 weeks of continuous antipsychotic medication. - Age between 18 and 45 years. - Participants selected from psychiatric hospitals and outpatient centres in Copenhagen and Aarhus. | Urban | January 1998 – December 2000. Follow-up at 1, 2, and 5 years after inclusion | - Psychotic dimension: SAPS - Negative dimension: SANS - Disorganised dimension: SANS - Substance use disorder: - Suicide attempt (lifetime): % - Suicide attempt (past year): GAF – symptoms, GAF – function. - Psychotic dimension: SAPS - Negative dimension: SANS - Disorganised dimension: SANS - GAF symptoms - GAF function - Client Satisfaction Questionnaire | At the 2-year mark, the OPUS group showed fewer psychotic and negative symptoms, better adherence to antipsychotic medication, reduced substance abuse, less need for psychiatric hospitalization, and greater satisfaction with treatment. However, by year 5, most of these positive effects were no longer observed, except for a lower need for institutionalization and fewer cumulative days of psychiatric hospitalization. OPUS was not more expensive than standard care and was, in some cases, more cost-effective. |
| Nugter et al. (2016); Netherlands | Observational. Prospective study.  Flexible Assertive Community Treatment (FACT) — Dutch FACT model. | N = 372 Medical diagnosis: SMI   - Schizophrenia or other psychotic disorders: 60.4% - Bipolar disorder: 7.72% - Depressive disorders: 7.38% - Personality disorders: 10.07%   Age: Mean = 44.12 years, SD = 12.21 Sex: 55.38% men  Groups:   - N participants with complete clinical data = 298, 56.37% men - N participants without complete clinical data = 74, 51.35% men | Individuals with SMI treated by specialized case management teams, included in FACT teams | Rural | July 2009 - December 2011 | - HoNOS (psychosocial functioning) - MANSA (quality of life) - CANSAS (unmet needs) - Clinical referral (based on PANSS) - Days of hospitalization, number of admissions, outpatient contacts - Satisfaction with care, social inclusion (housing, employment, social network) | There was an increase in clinical referrals, along with a reduction in both the number of hospital admissions and days of hospitalization. Significant improvements were observed in quality of life and treatment adherence. Unmet needs were reduced, and a slight increase in employment was noted, while a high rate of independent living was maintained. Greater stability was achieved among patients managed under FACT who did not require intensive ACT-level care. |
| Pérez de León et al. (2011); USA | Uncontrolled prospective pre-post study.  Adapted Assertive Community Treatment (ACT) combined with Integrated Treatment (IT) for homeless men with substance use or dual diagnosis. | N = 103 Medical diagnosis: Substance use disorder or dual diagnosis   - Alcohol abuse: 47% - Major depressive disorder: 44%   Age: Mean = 42 years, SD = 9.9 Sex: 100% men  Groups: N/A | Men, ≥18 years old Homeless for ≥1 year Diagnosis of substance use disorder or dual diagnosis confirmed by a program psychiatrist | Urban | N.D. | - ASI (Psychiatric composite) - BSI (Global Severity Index) - Substance use: days of use (alcohol, drugs), hospitalization, ASI (alcohol, drugs) - Housing stability: % with stable housing - ASI physical composite and hospitalizations - Employment: employment status, ASI (Employment composite) - Legal issues: ASI legal composite - Fidelity to the ACT model: DACTS | Significant improvements were observed in mental symptom severity, substance use, hospitalizations related to substance use, legal issues, physical health, and housing stability—rising from no stable housing at baseline to over half of participants in stable housing at 12 months. No significant improvement was found in employment overall, although participants with longer engagement showed gains. Most outcomes did not differ significantly between minority and non-minority groups. |
| Petersen et al. (2005); Denmark | RCT  Assertive Community Treatment (ACT) — enriched ACT for first‑episode psychosis (OPUS model, high‑intensity ACT with structured add‑ons). | N = 547 Medical diagnosis: First episode of psychotic illness Age:   - Integrated treatment group mean = 26.6 years, SD = 6.4; - Standard treatment group mean = 26.6 years, SD = 6.3   Sex:   - Integrated treatment group = 58% male; - Standard treatment group = 60% male   Groups:   - N intervention group (Integrated treatment) = 275 - N control group (Standard treatment) = 272 | - Age between 18 and 45 years. - Diagnosis within the schizophrenia spectrum according to ICD-10. - No continuous antipsychotic treatment for more than 12 weeks. | Urban | 1998 – December 2000. Follow-up at 1 and 2 years | - Scale for Assessment of Positive Symptoms (SAPS) - Scale for Assessment of Negative Symptoms (SANS) - Global Assessment of Functioning (GAF) - Number of hospital days - Number of admissions - Abuse or dependence diagnosis according to ICD-10 – SCAN - Client Satisfaction Questionnaire (CSQ-8) - Treatment adherence | The OPUS group showed a greater reduction in both psychotic and negative symptoms over one and two years, along with significantly higher treatment satisfaction. Substance use was lower in OPUS during the first year, and participants had fewer hospitalization days on average, although this difference was marginally significant. Treatment adherence was also better in OPUS, with fewer interruptions and more outpatient visits. |
| Petersen et al. (2007); Denmark | RCT  Assertive Community Treatment (ACT) — enriched ACT for first‑episode psychosis with integrated early‑intervention components (OPUS model). | N = 547 Medical diagnosis: First-episode schizophrenia spectrum disorders Age:   - No substance abuse group mean = 26.8 years, SD = 6.4 - Substance abuse group = 26.0 years, SD = 6.3   Sex:   - No substance abuse group = 64.4% male; - Substance abuse group = 78.8% male   Groups:   - N no substance abuse = 401 - N substance abuse = 146 | - Age between 18 and 45 years. - Diagnosis of schizophrenia spectrum psychosis according to ICD-10. - No continuous antipsychotic treatment for more than 12 weeks. - The condition must not be due to acute intoxication or withdrawal syndrome. | Urban | January 1998 – December 2000. Follow-up: 2 years | - SCAN (Schedules for Clinical Assessment in Neuropsychiatry) - SAPS (Scale for Assessment of Positive Symptoms) - SANS (Scale for Assessment of Negative Symptoms) - SAPS/SANS (disorganized dimension) - GAF – symptoms and function - Days of psychiatric hospitalization (bed days): - Clinical record - Treatment adherence (no outpatient visits): Clinical record. - Proportion: “non-adherent” applied to any patient with no recorded outpatient visit during the last year of follow-up. - Premorbid functioning: PAS (Premorbid Adjustment Scale) - Duration of untreated psychosis (DUP): Structured interview - Social integration (number of friends and family): Specific questionnaire | The OPUS model led to reduced substance abuse, fewer hospitalization days, and better treatment adherence compared to standard care. Improvements were observed in negative and disorganized symptoms, though not in psychotic symptoms. No significant differences were found in social functioning or quality of life. Incorporating a targeted substance abuse treatment module may enhance outcomes further. |
| Pope et al. (2014); Canada | Observational. Retrospective pre-post study.  High‑fidelity ACT model (DACTS‑verified) | N = 29 Medical diagnosis: SMI   - Schizophrenia: 48.3% - Schizoaffective disorder: 17.2% - Bipolar disorder: 31% - Depression: 3.4%   Age: Median = 43 years (range 20–66) Sex: 82.8% men  Groups: N/A | ACT program users, contacted via purposive sampling | Rural | N.D. | - Psychiatric hospitalization days - Emergency room visits (ER) - Program satisfaction scale (CMHEI) | The ACT program led to a significant reduction in hospitalization days and emergency room visits. Participants reported high satisfaction with the program, with the most valued aspects being emotional support, interpersonal interaction, instrumental assistance, and the team's availability. |
| Poremski et al. (2016); Canada | RCT  Housing First with Assertive Community Treatment (ACT) for high‑needs participants; Intensive Case Management (ICM) for moderate‑needs participants. | N = 2148 Medical diagnosis: mental illness Age:   - High needs (HF ACT): group mean = 38.9 years, SD = 10.8; Control group = 39.9 years, SD = 11.2 - Moderate needs (HF ICM): group mean = 42.2 years, SD = 11.1; Control group = 42.1 years, SD = 11.3   Sex:   - High needs ACT group = 68% male; Control group = 68% male - Moderate needs ICM group = 65% male; Control group = 68% male   Groups:   - N intervention group (HF): High needs ACT = 469; Moderate needs ICM = 689 - N control group: High needs = 481; Moderate needs ICM = 509 | - Legal adult age (18 years or older; 19 in British Columbia). - Housing status: Homeless or precariously housed. - Presence of a mental disorder, with or without a co-occurring substance use disorder, diagnosed according to DSM-IV criteria using the MINI interview at intake. | Urban | October 2009 – June 2011. Data collection continued until June 2013 | - Employment status (frequency, type of work, duration, income) - Income sources (employment, government support, street activities, illegal activities) - Community functioning (Multnomah Community Ability Scale – MCAS) | Housing First did not lead to a significant increase in employment compared to the control group. In fact, participants were less likely to obtain competitive employment. Access to subsidies and government support may have reduced the incentive or need to work. No significant differences in income were observed between the groups. |
| Razali et al. (2015); Malaysia | Observational. Retrospective comparative study.  Modified Assertive Community Treatment (modified ACT) — reduced‑fidelity ACT model in Malaysia. | N = 88 Medical diagnosis: Schizophrenia Age: Not specified Sex: Not specified  Groups:   - N Intervention group (modified ACT) = 44 - N Control group = 44 | - Diagnosis of schizophrenia (DSM-IV-TR) - Age: 23–60 years - GAF score between 51–70 (mild to moderate symptoms) - ≥2 psychiatric hospitalisations lifetime Illness duration ≥5 years - Matched by age and gender with control group - Exclusion: homelessness, substance abuse, alcoholism, moderate-to-severe intellectual disability, or severe medical illness | Urban | October 2010 – January 2011 | - Number of hospitalizations (2 years before vs. after ACT). - Average days of hospital stay per admission (ALOS). | The modified ACT group experienced a significant reduction in readmissions, although no differences were found in the length of hospitalization. While less intensive than the classic model, modified ACT proved to be a useful intervention. |
| Rezansoff et al. (2017); Canada | RCT  Scattered‑site Housing First with Assertive Community Treatment (ACT) vs. Congregate Housing First with on‑site supports vs. Treatment as Usual (TAU). | N = 165 Medical diagnosis: Schizophrenia Age:   - Scattered-site group mean = 40.4 years, SD = 11.0 - Congregate-site group mean = 38.7 years, SD = 11.1 - TAU group mean = 41.5 years, SD = 11.1   Sex:   - Scattered-site group = 78% male - Congregate-site group = 75% male - TAU group = 73% male   Groups:   - N Scattered-site Housing First (SHF + ACT) = 51 - N Congregate Housing First (CHF) = 57 - N Treatment as Usual (TAU) = 57 | - Canadian citizenship. - Aged at least 19 years. - Meeting criteria for homelessness or precarious housing. - Diagnosis of schizophrenia within the past 10 years according to administrative ICD-9 records (codes 295.0–295.9). - Antipsychotic treatment initiated prior to consent. | Urban | October 2009 – June 2011. Study end: March 31, 2013. Mean follow-up: 2.6 years | - Antipsychotic adherence: Medication Possession Ratio (MPR) - Treatment duration - Number of pharmacy visits - Physical and mental health status: SF-12 - Multnomah Community Ability Scale (MCAS) | Adherence to antipsychotic medication was significantly higher in the Scattered-Site Housing First (SHF) group compared to Treatment as Usual (TAU), while no significant differences were observed between the Congregate Housing First (CHF) group and TAU. SHF participants also had more pharmaceutical transactions annually. The study concludes that the SHF model combined with ACT is more effective in improving medication adherence than the CHF model. |
| Rohenkohl et al. (2022); Germany | Observational. Prospective longitudinal study.  Integrated care with Therapeutic ACT (TACT) — long‑term ACT‑based model in a cross‑sectoral system. | N = 329 Medical diagnosis: SMI Age: Mean = 36.70 years, SD = 14.32 Sex: 47.1% men Groups: N/A | - Age ≥12 years - Diagnosis of severe psychotic disorder - Informed consent - BPRS score ≥ 40 | Urban | May 2007 – June 2021 | - QoL: Q-LES-Q-18 - Psychopathology: BPRS - Functioning: GAF - Clinical severity: CGI-S (global, depression, cognitive, positive, negative) - Traumatic factors: traumatic events before age 18 - Duration untreated psychosis (DUP) - Comorbidities: psychiatric, somatic, addictive Z diagnoses (ICD-10) - Evaluations every 6 months for 5 years. | Quality of life (QoL) improved significantly during the first six weeks of intervention, followed by a period of stabilization. Childhood trauma before age 18 was linked to lower QoL over the five-year follow-up. Early reductions in depressive and positive symptoms predicted greater QoL improvements. Factors such as age, sex, diagnosis, or duration of untreated psychosis (DUP) did not significantly influence QoL trajectories. The most responsive domains were physical health and subjective well-being, while social relationships and leisure activities showed less change. |
| Salkever et al. (1999); USA | RCT  Program for Assertive Community Treatment (PACT) — full‑fidelity ACT model. | N = 144 Medical diagnosis: SMI Age:   - PACT group: Mean = 35.0 years - Control group: Mean = 36.3 years   Sex:   - PACT group: 57.1% male - Control group: 50.9% male   Groups:   - Intervention group (PACT) = 91 - Control group = 53 | - Age between 18 and 65 years. - Diagnosis of schizophrenia, schizoaffective disorder, bipolar disorder, or other psychotic disorder (DSM-III-R). - History of multiple hospitalizations or high risk of intensive service use. - Difficulties with treatment, independent living, or daily functioning. | Urban | August 1989 – July 1991. Follow-up: 18 months after each patient’s admission | - Psychiatric hospital use during the 18-month follow-up - Number of hospitalizations - Total number of days hospitalized - Brief Psychiatric Rating Scale (BPRS) - Global Assessment of Functioning (GAF) - Psychiatric hospitalization costs: Data from Medicaid and hospital records | PACT reduced the likelihood of hospitalization by approximately 40%, although it did not decrease the number of hospital days among those who were admitted. The program's effect was more pronounced in older patients, and lower staff-to-patient ratios further enhanced its impact on reducing hospitalizations. No significant differences were observed based on patient race. While hospital costs were lower in the PACT group, total program costs were not analyzed. |
| Salyers et al. (2011); USA | Observational. Retrospective cohort study.  Assertive Community Treatment (ACT) teams with integrated Illness Management and Recovery (IMR) — ACT plus curriculum‑based recovery intervention. | N = 498 Medical diagnosis: SMI Age: Mean = 40.6 years, SD = 12.2 Sex: 57% men  Groups: N/A | - Participation in one of five ACT teams providing Medicaid services between 2003 and 2008 - Available data in state records and Medicaid claims - Enrolled in ACT and had (or had not) received the Illness Management and Recovery (IMR) intervention | Mixed | July 2003 – June 2008 | - Psychiatric inpatient days. - Psychiatric emergency department visits | Users who participated in Illness Management and Recovery (IMR) had 50% fewer hospitalization days compared to those receiving ACT alone. Program graduates also had 64% fewer psychiatric emergency room visits. These positive effects remained significant after adjusting for age, diagnosis, substance use, and other variables. Program dropout was more common among younger individuals, those with lower education levels, and those with substance use disorders. |
| Santos et al. (1993); USA | Observational. Retrospective pre-post study.  Assertive Community Treatment (ACT) — full‑fidelity continuous treatment team model. | N = 51 Medical diagnosis: Schizophrenia or schizoaffective disorder   - Schizophrenia: 76% - Schizoaffective disorder: 24%   Age: Mean = 33.4 years, SD = 11.77 Sex: 53% men  Groups: N/A | - Patients aged 18–65 years with: - Primary diagnosis of schizophrenia or schizoaffective disorder - High use of psychiatric hospitalization - History of nonadherence to medication and missed appointments | Mixed | 5 years prior to program entry - follow-up during intervention: minimum 12 months | - Days of hospitalization per month/patient. - Residential status (hospital, supervised living, family living, independent living) - Estimated costs of hospital vs. community treatment. | After the implementation of ACT, there was a substantial reduction in hospitalization days. Most patients who had been living in hospitals or nursing homes transitioned to independent living, with only a very small number requiring more restrictive settings. The program also led to a notable decrease in overall treatment costs, primarily due to reduced hospitalization expenses, while maintaining or improving community-based care. |
| Schöttle et al. (2018); Germany | Uncontrolled prospective longitudinal study.  Integrated care model including Therapeutic Assertive Community Treatment (TACT) — high‑fidelity ACT embedded in a multi‑sectoral system. | N = 115 Medical diagnosis: Schizophrenia or Bipolar I Disorder Age: Mean = 41.8 years, SD = 12.9 Sex: 44.3% men  Groups: N/A | - Diagnosis of a schizophrenia-spectrum disorder or bipolar disorder with psychotic features, assessed using the Structured Clinical Interview for DSM-IV Axis I Disorders (SCID-I). - Aged ≥18 years. - Current hospitalization due to an acute illness state, assessed by a psychiatrist. - Severity criteria: total score ≥40 on the Brief Psychiatric Rating Scale (BPRS), along with fulfilment of additional severity criteria for specific symptoms. | Urban | May 2007 - December 2013 | - BPRS scale - CGI-S scale - GAF scale - Q-LES-Q-18 - CSQ-8 - Treatment adherence: assessed by the clinical team - Days of hospitalization - Involuntary admissions | Sustained improvements were observed in psychopathology, illness severity, functioning, and quality of life. Hospitalization days and involuntary admissions declined significantly over time, while treatment adherence increased markedly. Long-term patient satisfaction with care remained high throughout the follow-up period. |
| Schöttle et al. (2019); Germany | Uncontrolled prospective longitudinal study.  Integrated care model including Therapeutic Assertive Community Treatment (ACCESS with ACT) — high‑fidelity ACT embedded in a diagnosis‑specific, multi‑sectoral system. | N = 171 Medical diagnosis: SMI Age: Mean = 42.31 years, SD = 13.36 Sex: 42.7% male  Groups: N/A | - Diagnosis of a schizophrenia-spectrum disorder (i.e., schizophrenia, schizophreniform disorder, schizoaffective disorder, delusional disorder, or psychotic disorder not otherwise specified) or bipolar disorder with psychotic  features, all assessed with the Structured Clinical Interview for DSM-IV Axis I Disorders (SCID-I) (62) - Aged ≥18 years - Present hospitalization because of an acute illness state as assessed by a psychiatrist - Presence of a certain severity of illness as assessed with the Brief Psychiatric Rating Scale, 24-item version  (BPRS),(63) with (1) BPRS total score ≥40 points and (2) fulfillment of 1 of the following eight criteria: ≥6 points  on item 10 (hallucinations); ≥6 points on item 11 (unusual thought content); ≥6 points on item 15 (conceptual  disorganization); ≥ 10 total points on items 3 and 4 (depressive-suicidal syndrome); ≥6 points on item 4  (suicidality); ≥15 total points on items 8, 9, and 21 (manic syndrome); ≥15 total points on items 6, 12, and 20  (disruptive behavior syndrome); or ≥15 total points on items 13, 16, and 17 (negative syndrome) | Urban | May 2007 – March 2012. 48-month follow-up | - Rate of involuntary admissions - BPRS - CGI-S - GAF - Q-LES-Q-18 - CSQ-8 P - Treatment adherence: defined as taking 80% or more of prescribed medication - Treatment disengagement: defined as dropping out of the program without practical reasons | The ACCESS program led to a significant reduction in involuntary admissions, along with improvements in symptom severity, global functioning, and treatment adherence. Participants reported high satisfaction with care, and the program maintained a low dropout rate. |
| Schröter et al. (2022); Germany | Observational. Prospective longitudinal study.  Integrated care model with Therapeutic Assertive Community Treatment (IC‑TACT) — high‑fidelity ACT variant within a multi‑sectoral network. | N = 418 Medical diagnosis: SMI   - Affective psychosis: 29.9% - Non-affective psychosis: 70.1%   Age: Mean = 36.17 years, SD = 14.03 Sex: 47.8% men  Groups: N/A | - Aged ≥12 years, DSM-IV-TR diagnosis of a psychotic disorder (affective or non-affective), informed consent. - Exclusions: substance-induced or medically induced psychosis, intellectual disability | Urban | May 2007 – September 2019 | - Qol (Q-LES-Q-18) - Negative symptoms (BPRS items 13, 16, 17, 18) - Depression (BPRS item 3) - Anxiety (BPRS item 2) - Functioning (GAF), - Severity (CGI) - Longitudinal correlations (cross-panel models) | Significant improvements in quality of life and symptomatology were observed after six months of intervention. Negative symptoms were identified as a key mediator of the intervention's effect on quality of life, with improvements in depression occurring indirectly through reductions in negative symptoms. Longitudinal analysis showed that the initial gains were maintained but stabilized by 12 months. |
| Siskind et al. (2009); USA | Observational. Cross-sectional comparative study.  Assertive Community Treatment (ACT) — comparison of rural vs. urban ACT implementations; both operate as ACT programs with variable fidelity. | N = 502 Medical diagnosis: Psychiatric disorder Age: Mean = 44 years Sex: Not specified  Groups:   - N NC group = 440 - N MA group = 62 | Population served by ACT teams in two regions | Mixed | 2006-2007 | - Diagnosis (DSM) - GAF scale - Employment, housing, hospitalization, emergency visits - Education, judicial supervision, tobacco use - Family support - Frequency and type of ACT team contacts | Diagnoses and global functioning were comparable across both regions. The rural North Carolina (NC) region showed greater family support, while the urban Massachusetts (MA) region had more frequent contacts, greater specialization, and higher fidelity to the ACT model. Hospitalization and employment outcomes were largely similar, though MA had a higher rate of admissions to detoxification services. NC reported much lower visit and meeting frequency, with reduced model fidelity attributed to limited resources, geographic distance, and staffing constraints. |
| Siskind et al. (2021); Australia | Observational retrospective.  Hybrid Assertive Community Treatment (ACT) model with intensive psychosocial rehabilitation — Mobile Intensive Rehabilitation Team (MIRT). | N = 167 Medical diagnosis: SMI   - Schizophrenia: 76% - Schizoaffective disorder: 15% - Bipolar disorder: 7.8% - Post-traumatic stress disorder: 1.2%   Age: Mean = 32.5 years, SD = 11.2 Sex: 58.78% men  Groups: N/A | All consumers who were working with MIRT at any time between 1 January 2017 and 30 August 2019 were included in the analysis. MIRT: people with severe and persistent mental illness with significant psychosocial functioning deficits, with a willingness (or absence of overt refusal) to engage in psychosocial rehabilitation services, with a goal of building on personal strengths. achieving recovery goals and improving illness self management. | Urban | January 2017- August 2019 | - Age at entry to MIRT - Primary clinical diagnosis - Mental Health Act (MHA) status at entry, including community treatment order, forensic order, voluntary status - Medication status at entry and discharge: Clozapine use, Depot antipsychotic use - HoNOS - Score at referral to MIRT, Score at discharge from MIRT - Duration of contact with MIRT (in months) - Psychiatric hospitalization during the period with MIRT - Discharge destination, categorized as: out of case-management (e.g. primary care, private psychiatrist, clozapine clinic without case-management), ongoing case-management (e.g. continuing care team, sub-acute residential service) | About half of the participants were discharged from case management. Hospitalization during the program was associated with a longer duration in MIRT. Patients prescribed clozapine or those with higher symptom severity at discharge, as measured by HoNOS, were less likely to be discharged from case management. |
| Slade et al. (2013); USA | Quasi-experimental (cost-effectiveness analysis)  Assertive Community Treatment (ACT) — VA Mental Health Intensive Case Management (MHICM) programs operating as high‑fidelity ACT teams. | N = 6030 Medical diagnosis: Schizophrenia or Bipolar Disorder Age:   - ACT group: Mean = 50.1 years - Control group: Mean = 51.3 years   Sex:   - ACT group: 90.4% male - Control group: 90.5% male   Groups:   - N intervention group (ACT) = 2010 - N control group = 4020 | - Diagnosis of schizophrenia or bipolar disorder - ≥3 admissions or >30 days of psychiatric hospitalization in the previous 12 months - Resident within ≤60 miles of a VA hospital | Mixed | 2001 – 2004 | - Mental health care costs (total, hospital, residential, outpatient) - Number of psychiatric hospitalization days - Use of residential services and partial hospitalization - Comparisons with non-ACT group using IV methods and matching | Enrollment in ACT led to a moderate reduction in hospitalization days but did not significantly lower the probability of hospital admission. On average, total costs were higher for ACT participants compared to those not enrolled in the program. Cost neutrality was only achieved among patients with a high number of prior hospitalization days. Over time, the general decline in hospital use reduced the number of patients likely to benefit financially from ACT. |
| Smith et al. (2010); USA | Observational. Prospective long-term follow-up study.  Forensic Assertive Community Treatment (FACT) within a forensic continuum‑of‑care model. | N = 91 Medical diagnosis: Dual diagnosis Age: Mean = 36.4 years Sex: 97.8% men  Groups: N/A | - Individuals with dual diagnosis - Judicial status of “Not Guilty by Reason of Insanity” (NGRI) - High risk, previously considered “too dangerous to be released” - Exclusions: low-risk individuals discharged directly to the community | Urban | February 1996 – December 2004. Follow-up: until July 2006 | - Criminal recidivism (rearrests and reincarcerations). - Re-hospitalization (residential or state) - Maintenance of abstinence - Significant activity (≥10h/structured week) - Residential stability - Length of residential and community stay” | Among participants who completed the program, most remained in the community for an extended period. Rates of re-arrest, re-incarceration, and hospital readmission were very low. The majority maintained abstinence, achieved stable housing, and engaged in meaningful activities. Overall, the program demonstrated high success rates, with nearly half of participants meeting the most stringent criteria for success. |
| Somers et al. (2013); Canada | RCT  Housing First with Assertive Community Treatment (HF‑ACT) — ACT delivered as part of a Housing First intervention for homeless adults with severe mental illness and high needs. | N = 497 Medical diagnosis: Mental disorder   - Psychotic Disorder/Schizophrenia = 53% - Major Depressive Episode = 40% - PTSD = 26% - Manic or Hypomanic Episode = 19% - Panic Disorder = 21% - Mood disorder with psychotic features = 17% - Substance Dependence = 58% - Alcohol Dependence = 24%   Age: Mean = 40.8 years, SD = 11.0 Sex: 73% male  Groups:   - N High needs group = 297 (Sex: 74% male) - N Moderate needs group = 200 (Sex: 71% male) | - Minimum age of 19 years. - Current diagnosis of a mental disorder. - Homelessness or precarious housing conditions. | Urban | Not specified. Follow-up: 24 months post-randomization | - Mini-International Neuropsychiatric Interview (MINI) - Colorado Symptom Index (CSI) - EuroQol 5D (EQ-5D - SF-12 Health Survey - Multnomah Community Ability Scale (MCAS) - Medical comorbidities - Residential Time-Line Follow-Back (RTLFB): Housing history, frequency of moves, type of housing - Observer-rated Housing Quality Scale (OHQS) - Vocational Time-Line Follow-Back (VTLFB) - Health, Social, and Justice Service Use Inventory (HSJSU) - Health Service Access Items (ACC): - Community Integration Scale (CIS) - Quality of Life Index (QoLI-20) - Assessment Scale (RAS-22) - Global Appraisal of Individual Needs – Substance Problem Scale (GAIN-SPS) - Maudsley Addiction Profile (MAP): Alcohol and drug use, frequency and related harms | The study achieved a high follow-up rate over 24 months. No significant differences were found between the intervention and treatment-as-usual (TAU) groups in key outcomes such as housing stability, service use, quality of life, substance use, or interactions with the justice system. Both groups showed high rates of substance use and physical health issues. While Housing First was successfully implemented, it did not lead to better outcomes compared to other forms of support. Conditions of vulnerability remained prevalent, including high rates of hospitalization, psychiatric disorders, and substance dependence across all groups. |
| Sood et al. (2014); United Kingdom | Observational. Retrospective mirror-image study.  Assertive Community Treatment (ACT) — high‑fidelity ACT team within UK NHS. | N = 93 Medical diagnosis: SMI   - Schizophrenia: 71% - Bipolar affective disorder: 6.5% - Schizoaffective disorder: 22.6%   Age: Mean = 37.7 years, SD = 11.7 Sex: 70% men  Groups: N/A | - Had contact with the ACT team during its first 10 years (2000–2011) - History of severe psychotic disorder - Problems with service engagement | Mixed | September 2000 – February 2011 | - Total number of admissions. - Involuntary admissions (under Mental Health Act) - Bed days per person per year | The program resulted in a significant reduction in psychiatric hospitalizations, including fewer total and involuntary admissions. The number of hospital days per year also declined notably, with improvements sustained over both one-year and longer-term follow-up periods. Median hospitalization days decreased substantially, indicating consistent benefits across participants. |
| Sood et al. (2017); United Kingdom | Observational. Retrospective pre-post comparative study.  Assertive Community Treatment (ACT) — standard, full‑fidelity ACT model. | N = 475 Medical diagnosis: Psychosis Age:   - ACT group: Mean = 45.3 years - CMHT group: Mean = 47.7 years   Sex:   - ACT group: 66% men - CMHT group: 54.2% men   Groups:   - N intervention group (ACT) = 95 - N control group (CMHT) = 380 | - History of psychosis - Referred from ACT or CMHT teams - Transitioned to FACT teams | Mixed | December 2014 - January 2016 | - Number and duration of face-to-face contacts - Use of Crisis Resolution and Home Treatment teams (CRHT) - Days of hospitalization - Number of hospital admissions | Patients from CMHT showed a significant reduction in the use of crisis services and hospitalizations, while those from ACT experienced a smaller decline. There was an increase in the proportion of contacts carried out by support workers, along with a higher percentage of contacts taking place in community settings. |
| Stein et al. (1999); USA | Observational. Cross-sectional comparative study.  Assertive Community Treatment (ACT) — standard PACT‑model ACT program. | N = 59 Medical diagnosis: SMI   - Schizophrenia or schizoaffective disorder: 77.9%   Age:   - ACT group: Mean = 42.3 years, SD = 6.0 - Clubhouse group: Mean = 42 years, SD = 9.3   Sex: 54.2% men  Groups:   - N ACT group = 21 - N Clubhouse group = 38 | - Aged 18–55 years - Participation in the program for ≥1 year - Primary diagnosis of schizophrenia, schizoaffective disorder, bipolar disorder, or major depression - No hospitalizations or crises in the past year - Clinically approved for participation (not destabilizing) | Urban | N.D. | - Type of activity (employment, volunteering, program, leisure) - Hours worked - Preference and satisfaction with the activity - Number of friends - Satisfaction with relationships - Importance of support - Loneliness Scale (Young Loneliness Scale) - Community Integration: participation in church, cinema, social activities | Individuals stabilized in ACT and Clubhouse programs demonstrated similar levels of vocational activity, social support, and perceived loneliness. Although Clubhouse participants engaged more frequently in structured activities, overall functional profiles were comparable. These findings support the feasibility of transitioning stabilized ACT users to less intensive programs like Clubhouses without negatively impacting their psychosocial functioning. |
| Stobbe et al. (2014); Netherlands | RCT  Assertive Community Treatment for the Elderly (ACTE) — specialized ACT model adapted for older adults. | N = 62 Medical diagnosis: SMI Age:   - ACTE group mean = 74.4 years, SD = 7.0; - TAU group mean = 75.1 years, SD = 9.3   Sex:   - ACTE group = 50% male - TAU group = 33.3% male   Groups:   - N intervention group (ACTE) = 32 - N control group (TAU) = 30 | - Age ≥65 years (later lowered to 60). - Suspected severe mental illness (schizophrenia, major affective disorders). - Problems in at least four domains (daily functioning, daytime activities, addiction, financial issues, housing, somatic health, police contact). - Difficulty participating in treatment (history of involuntary admissions or abandonment of mental health care). | Urban | July 2008 – July 2010 | - Mental health system contact within the first 3 months - Number of dropouts during follow-up - Psychosocial problems over time - Number of unmet needs - Number and duration of psychiatric hospitalizations - Number of crisis contacts | The ACTE group was more effective in establishing early contact with patients and demonstrated a lower dropout rate compared to treatment as usual (TAU). Participants in ACTE also showed improved psychosocial functioning and a greater reduction in unmet needs than those receiving standard care. |
| Svensson et al. (2018); Sweden | Quasi-experimental longitudinal study.  Flexible Assertive Community Treatment (FACT) — Dutch‑model Flexible ACT implemented in Sweden. | N = 96 Medical diagnosis: SMI   - F20–F29 (schizophrenia, schizotypal, and delusional disorders): 92%   Age: Mean = 48 years, SD = 15 Sex: 49% men  Groups:   - N participants in long-term follow-up (n = 84) - N participants not in follow-up (n = 12) | - In contact with a Flexible ACT team with good fidelity - Need for intensive care due to: risk of relapse, suicidal risk, and medication nonadherence - Exclusions: current inpatient admission or new patients | Urban | 2014–2016: 18-month follow-up | - Objective Social Outcome Index (SIX) - Employment, housing, family relationships, friendships - Practical and Social Functioning Scale (PSF) - Hospitalizations, inpatient days - Psychiatric outpatient visits. | Significant improvements in daily functioning were observed at 18 months, along with gains in the "friendship" domain. A positive association was found between social relationships and functional outcomes. However, a longer duration in ACT was correlated with lower levels of daily functioning, possibly reflecting greater clinical severity. There was a slight but significant increase in days of admission and outpatient visits, though the rise in hospital admissions was limited to two teams with specific challenges such as high staff turnover or high rates of somatic comorbidity. |
| Sytema et al. (2007); Netherlands | RCT  Assertive Community Treatment (ACT) — standard, full‑model ACT. | N = 118 Medical diagnosis: SMI Age:   - ACT group: Mean = 41.5 years, SD = 11.6 - Standard care group: Mean = 41.6 years, SD = 10.9   Sex:   - ACT group: 75% male - Standard care group: 63% male   Groups:   - N Intervention group (ACT) = 59 - N Control group = 59 | - Patients with severe mental illness (Health of the Nation Outcome Scales [HoNOS] ≥15). - Long-term care in mental health services. - At risk of losing contact with healthcare services. | Rural | April 2004 – June 2005. Follow-up until August 2006 (maximum 2 years) | - Retention in mental health services (continued engagement) - Housing stability (number of homeless patients at study endpoint) - Number of hospitalization days (from clinical records) - Social Functioning Scale (SFS) - Brief Psychiatric Rating Scale (BPRS) - Camberwell Assessment of Needs (CANSAS) - Manchester Short Assessment of Quality of Life (MANSA) - Dartmouth Assessment of Lifestyle Interview (DALI) - Client Satisfaction Questionnaire (CSQ) | Assertive treatment was more effective at maintaining contact with patients but did not significantly reduce hospital days compared to standard care. No differences were observed in housing stability, psychiatric symptoms, social functioning, or quality of life. Patients receiving ACT tended to report higher satisfaction with their care. |
| Sytema et al. (2014); Netherlands | RCT  Assertive Community Treatment (ACT) — standard ACT team with added evidence‑based interventions (ACT‑Plus). | N = 159 Medical diagnosis: SMI Age: Mean = 36.9 years, SD = 10.8 Sex: 68% male  Groups:   - N intervention group (ACT plus) = 74 - N control group (standard ACT) = 85 | - HoNOS score >14. - Diagnosis of SMI - Difficulty adhering to treatment. | Urban | November 2008 – June 2009. Follow-up: 24 months. Data collection completed in November 2012 | - Hospitalization days - Outpatient contacts - Crisis contacts - PANSS (positive, negative, and general symptoms) - MADRS - MANSA - CANSAS - SOFAS - Participation in interventions (family, psychoeducation, cognitive-behavioral therapy, vocational support) | No significant differences in hospitalization outcomes were found between ACT Plus and standard ACT. Despite a high eligibility rate for the additional interventions, participation and completion rates were low. The high dropout rate limited the effectiveness of the enhanced interventions in the ACT Plus group. |
| Tempier et al. (2012); United Kingdom | RCT  Assertive Community Treatment (ACT) — specialized early‑intervention ACT model (LEO Team). | N = 107 Medical diagnosis: First-episode non-affective psychosis Age:   - Specialized care group mean = 25.7 years, SD = 6.1; - Standard care group mean = 26.0 years, SD = 6.7   Sex:   - Specialized care group = 53% male - Standard care group = 78% male   Groups:   - N intervention group (Specialized care) = 57 - N control group (Standard care) = 50 | - First-episode diagnosis of non-affective psychosis. - Age between 16 and 35 years. - Residents of Lambeth, London. | Urban | Subsample from a randomized study including patients with first-episode psychosis between January 2000 and October 2001 | - Positive and Negative Syndrome Scale (PANSS) - Global Assessment of Functioning (GAF) - Number of significant people in the patient’s life - Quality of life and treatment satisfaction | The ACT group showed significant improvement in negative symptoms, better global functioning, and a larger social support network compared to controls. |
| Thoegersen et al. (2019); Denmark | Quasi-experimental study with non-randomised control group.  Assertive Community Treatment (ACT) — standard, high‑fidelity ACT model. | N = 366 Medical diagnosis: SMI Age:   - ACT group: mean = 42.6 years, SD = 11 - Control group: mean = 43.4 years, SD = 12   Sex:   - ACT group = 59% male; - Control group = 62% male  Groups: - N intervention group (ACT) = 213 - N control group = 153 | - Aged 18–65 years - Diagnosis of SMI (ICD-10: F20, F22, F25, F31) - Difficulty engaging with standard community care - Resident in one of the four study catchment areas - At least one of the following criteria: - ≥4 hospital admissions in the past 2 years - ≥40 days of hospitalization in the past 2 years - Secondary diagnosis of substance abuse - Repeated failure to attend outpatient appointments (>25%) - Poor adherence to pharmacological treatment | Urban | September 2003 - February 2005. 24-month follow-up. | - Loss of contact with mental health services (defined as more than 6 months without contact) - Days of psychiatric hospitalization - Days of involuntary admission - Number of outpatient contacts (appointments, home visits, team meetings) - Adherence to antipsychotic medication - GAF - DTES - PANSS - CSQ - MANSA | ACT demonstrated better treatment adherence, with lower rates of lost contact with mental health services and higher medication adherence compared to controls. Hospitalization days were significantly reduced, and outpatient contacts increased in the ACT group. Social functioning improved, and patient satisfaction was higher with ACT. No significant differences were found in involuntary hospitalization days, substance abuse, or quality of life. |
| Tibbo et al. (1999); Canada | Observational. Retrospective pre-post observational study without a control group  Assertive Community Treatment (ACT) — standard PACT‑model ACT program. | N = 295 Medical diagnosis: SMI   - Schizophrenia = 35.6% - Bipolar disorder = 19.7% - Major depression = 10.2%   Age: Mean = 40.7 years, SD = 10.3 Sex: 45.4% male  Groups: N/A | - Aged 18-65 years. - Diagnosis of a severe and persistent mental illness. - Meeting at least 3 of the following criteria: - History of multiple psychiatric hospitalizations - Difficulty functioning in the community (due to lack of skills or support) - Nonadherence to pharmacological treatment - Refusal of traditional outpatient services - High risk of psychiatric readmission without intervention | Urban | April 1992 - Martch 1996 | - Number of psychiatric hospitalizations - Total number of hospitalization days - Number of emergency visits for psychiatric reasons - Number of patients who visited the emergency department - Average length of hospital stay | ACT treatment significantly reduced hospitalizations and emergency service use among individuals with severe mental disorders. Key outcomes included notable decreases in psychiatric hospital admissions, average length of hospital stays, total hospitalization days, and psychiatric emergency visits. Patients experienced fewer and shorter hospitalizations as well as fewer emergency visits. However, improvements in quality of life and psychiatric symptoms were not assessed, indicating the need for further research in these areas. |
| Tibbo et al. (2001); Canada | Observational. Prospective longitudinal study.  Assertive Community Treatment (ACT) — standard ACT program. | N = 411 Medical diagnosis: SMI   - Schizophrenia: 46% - Bipolar disorder: 23.8% - Major affective disorder: 11.2% - Schizoaffective disorder: 10.0%   Age: Mean = 40.1 years (range: 18–64) Sex: 49.6% men  Groups: N/A | - Aged 18–65 years - Severe and persistent mental illness - Meeting ≥3 of the following criteria: - Multiple psychiatric hospitalizations - Difficulty functioning in the community - Treatment nonadherence - Refusal of outpatient follow-up - High risk of prehospitalization without intensive community care | Urban | N.D. | GAF (Global Assessment of Functioning) | Enrollment in ACT was associated with significant functional improvements in patients with severe mental illness, especially those with low initial global functioning and certain diagnoses such as schizophrenia, schizoaffective disorder, dysthymia, or bipolar disorder. Not all diagnostic groups showed the same level of improvement, with patients starting at higher functioning levels benefiting less, possibly due to a ceiling effect. ACT demonstrated a sustained positive impact on global functioning over three years. |
| Tinland et al. (2020); France | RCT  Housing First with Assertive Community Treatment (HF‑ACT) — Housing First program delivered with a high‑fidelity ACT team. | N = 703 Medical diagnosis: Schizophrenia or bipolar disorder Age:   - HF group mean = 38.1 years, SD = 9.7 - TAU group mean = 39.4 years, SD = 10.3   Sex:   - HF group = 80.2% male - TAU group = 84.9% male   Groups:   - N intervention group (HF) = 353 - N control group (TAU) = 350 | Diagnosis of schizophrenia or bipolar disorder, moderate to severe disability (MCAS ≤62), and meeting at least one of the following conditions: two or more psychiatric hospitalizations in the last 5 years, alcohol or substance use disorder, or history of arrest or incarceration in the past 2 years. In addition, participants had to be covered by the French state health insurance and be capable of providing informed consent. | Urban | August 2011 – April 2014 | - Number of emergency room visits, psychiatric hospital admissions, and hospital days at 24 months - Recovery (Recovery Assessment Scale) - Mental health symptoms (MCSI) - Quality of life (PCS, MCS, SQOL-18) - Medication adherence (MARS) - Addiction problems: alcohol and other substances - Days in stable housing - Costs: total, healthcare, housing, and justice system | There were no differences between groups in the number of hospital admissions or emergency visits; however, the Housing First group had significantly fewer hospital days. Additionally, participants in the Housing First group experienced greater housing stability and reported better psychological well-being and autonomy, as measured by the S-QOL scale. No differences were found in physical health, mental health symptoms, or substance and alcohol dependence. Economically, Housing First was more cost-effective, leading to overall savings per patient over two years, particularly in healthcare and housing expenses. |
| Tomar et al. (2020); USA | Observational. Retrospective cohort study.  Assertive Community Treatment (ACT) — standard ACT and Forensic ACT (FACT) teams operating under national fidelity standards. | N = 671 Medical diagnosis: SMI Age:  Non-transition group: Mean = 41.18 years, SD = 12.6  Transitioned group: Mean = 42.39 years, SD = 12.11 Sex:  Non-transition group: 61.38% male  Transitioned group: 62.96% male  Groups: N Non-transitioned group = 347 N Transitioned group = 324 | - Diagnosis of SMI (mainly schizophrenia or bipolar disorder) - Clear functional impairment - At least two hospitalizations within one year | Urban | N.D. | - Diagnosis (schizophrenia vs. mood disorders) - SSI/SSDI recipient (SSI (Supplemental Security Income) y SSDI (Social Security Disability Insurance)) - Previous hospitalizations - Previous incarceration - Recidivism post-transition (hospital or incarceration) - Quality of life scales (12 items), - Empowerment, Community functioning (COS) | Patients with mood disorders, a history of incarceration, or without SSDI benefits were more likely to transition to less intensive programs. Those with schizophrenia or receiving SSDI benefits were less likely to transition. Post-transition recidivism was higher among individuals with prior hospitalizations but not among those with previous incarcerations. No significant differences were found in quality of life, empowerment, or community functioning between those who transitioned and those who did not. |
| Udechuku et al.(2005); Australia | Observational. Retrospective cohort study.  Assertive Community Treatment (ACT) — standard, high‑fidelity ACT team (Madison‑model/mobile outreach ACT). | N = 43 Medical diagnosis: SMI   - Schizophrenia: 79% - Schizoaffective disorder: 19% - Bipolar affective disorder: 2%   Age: Mean = 38 years (Range: 23–62) Sex: 56% men  Groups: N/A | - Individuals with severe and persistent mental illness - Treated by NEMSTS for at least 12 months (for effectiveness analysis): North East Mobile Support and Treatment Service - DSM-IV diagnosis and complete clinical follow-up | Urban | September 2001: analysis period 12 months before and 12 months after ACT entry. | - Number of psychiatric readmissions. - Days of hospitalization - Economic cost of the service - Psychopharmacological medication used - Functional characteristics (GAF), sociodemographic data and comorbidities. - Number of hospital admissions - Days of hospitalization - DSM-IV diagnosis - Global functioning scale (GAF) - Hospital costs (AUD) - Type of psychiatric medication used (typical, atypical, mood stabilisers, etc.) | The ACT model implemented by NEMSTS led to a substantial reduction in psychiatric inpatient days, with a notable clinical and economic impact despite a non-significant decrease in admissions. The model demonstrated feasibility and sustainability under real-world clinical conditions. Economic savings were considerable, and improvements were sustained post-intervention. Only a small proportion of patients required ACT follow-up beyond four years. |
| Valenstein et al. (2013); USA | Observational. Retrospective cohort study.  Assertive Community Treatment (ACT) — standard ACT delivered within the U.S. Veterans Affairs (VA) system (VA Mental Health Intensive Case Management). | N = 1526 Medical diagnosis: Schizophrenia Age: Mean = 50.2 years, SD = 9.7 Sex: 89% male  Groups:   - N intervention group (ACT) = 763, sex 90% male - N control group (non-ACT) = 763, sex 88% male | - Diagnosis of schizophrenia in the past 12 months - High previous hospital use (≥3 psychiatric admissions or ≥30 days of psychiatric hospitalization in the past year) - Living within 60 miles of an ACT team - ≥90 days of outpatient care in each of five consecutive six-month periods - At least one antipsychotic prescription 6–12 months before the index date | Mixed | October 2000 - September 2004. 24-months follow-up | - Adherence to antipsychotics (Medication Possession Ratio (MPR)) - Level of participation in ACT (full, partial, none) - Number of psychiatric admissions | Participation in ACT programs is associated with significant and sustained improvements in antipsychotic medication adherence among veterans with schizophrenia. Higher levels of participation correspond to a greater likelihood of good adherence. However, these adherence improvements were not directly linked to reductions in psychiatric hospital admissions during follow-up. |
| van Vugt et al. (2011); Netherlands | Observational. Prospective longitudinal study.  Assertive Community Treatment (ACT) — standard ACT teams with partial‑fidelity implementation (pre‑FACT era, Dutch ACT). | N = 530 Medical diagnosis: SMI   - Schizophrenia: 77%   Age: Mean = 41.6 years, SD = 11.3 Sex: 71% men  Groups: N/A | Meeting at least 2 of the following:   - Episode of homelessness in the past year - ≥6 outpatient contacts per month in the past year - GAF score ≤ 40 - ≥2 admissions or ≥50 days of hospitalization in the past year | Mixed | 2005–2008 (24-month follow-up) | - HoNOS (total functioning and sub-scales) - CANSAS (needs met/not met) - Days of psychiatric hospitalization - Homeless days - Socio-demographic data and use of services | Greater fidelity to the ACT model was associated with improvements in symptoms and reductions in days spent homeless. No significant association was found with hospitalization days or unmet needs. The team structure, including shared caseloads, daily meetings, and strong leadership, was the component most strongly linked to better outcomes. |
| van Vugt et al. (2012); Netherlands | Observational. Prospective cohort study.  Assertive Community Treatment (ACT) — standard Dutch ACT teams with partial‑fidelity implementation, including optional integration of consumer‑providers. | N = 530 Medical diagnosis: Psychiatric diagnosis   - Schizophrenia: 77%   Age: Mean = 41.6 years, SD = 11.3 Sex: 71% men  Groups: N/A | Meeting at least 2 of the following:   - Episode of homelessness in the past year - ≥6 outpatient contacts per month in the past year - GAF score ≤ 40 - ≥2 admissions or ≥50 days of hospitalization in the past year | Mixed | 2005-2008 | - HoNOS - CANSAS - Working Alliance Scale - Psychiatric hospitalization days - Days in homelessness - Presence of consumer-provider in the team | The involvement of consumer-providers was linked to improved functioning, fewer days spent homeless, and greater fulfillment of personal needs, along with fewer unmet personal needs. However, it was also associated with an increased number of psychiatric hospitalization days. No significant effects were observed on the total number of needs met, total unmet needs, or therapeutic alliance. |
| van Vugt et al. (2014); Netherlands | RCT  Assertive Community Treatment (ACT) — standard Dutch ACT teams with partial‑fidelity implementation | N = 530 Medical diagnosis: Psychiatric diagnosis   - Schizophrenia: 77%   Age: Mean = 41.6 years, SD = 11.3 Sex: 71% men  Groups: N/A | Meeting at least 2 of the following:   - Episode of homelessness in the past year - ≥6 outpatient contacts per month in the past year - GAF score ≤ 40 - ≥2 admissions or ≥50 days of hospitalization in the past year | Mixed | 2005-2008 | - HoNOS item 3 - Days of hospitalization due to addiction - HoNOS (except addiction item) - CANSAS - Days in prison - Period of homelessness | Significant improvements were observed over time in addiction problems and hospitalization days related to addiction. However, no association was found between fidelity to the ACT model (either overall or specific to addiction) and these improvements. Patients with greater psychosocial deterioration and a history of homelessness experienced less improvement. |
| Vidal et al. (2020); Switzerland | Observational. Retrospective cohort study.  Assertive Community Treatment (ACT) — standard ACT with moderate fidelity and local adaptations (Geneva ACT model). | N = 55 Medical diagnosis: SMI   - Schizophrenia: 55.2% - Other psychotic disorders: 13.8% - Schizoaffective disorders: 20.7% - Bipolar or major depressive disorder: 10.3% - Current substance abuse or dependence: 20.7%   Age: Mean = 48.8 years, SD = 10.6 Sex: 56.4% men  Groups:   - N patients who participated in the long-term follow-up (n = 29) - N patients who did not (n = 26) | All patients from the beginning of the program, both at inclusion and after 6 months. | Urban | October 2007 - August 2009. Long term follow-up: September 2016 - August 2017 | - Total number of hospitalizations - BPRS - MARS - WHOQOL-BREF - MCAS - RAS | There was a significant reduction in the average number of hospitalizations per year. Sustained improvements were observed in symptoms, quality of life, social functioning, and attitudes toward medication. At follow-up, the majority of participants remained in treatment, with over half receiving care from a private psychiatrist. |
| Wasmer et al. (1999); USA | Observational. Retrospective cohort study.  Assertive Community Treatment (ACT) — standard ACT adapted to a flexible‑duration, intermittent‑access model (Thresholds Bridge ACT). | N = 64 Medical diagnosis: SMI Age: Not specified Sex: Not specified  Groups: N/A | - High utilization of state hospital beds - History of multiple psychiatric admissions - Resident in the “Back of the Yards” neighborhood | Urban | N.D. | - Number of state psychiatric hospitalizations - Total days of state hospitalization - Participation in other community services after - ACT discharge | State hospitalization use decreased drastically during participation in ACT. Although hospitalization rates increased after discharge, they did not return to pre-ACT levels. The flexible ACT model allowed more individuals to be treated while maintaining good outcomes. It was concluded that the model can be both sustainable and effective even with limited duration, provided that follow-up is maintained. |
| Waynor et al. (2020); USA | Observational. Cross-sectional study.  Assertive Community Treatment (ACT) — standard, fully model‑conforming ACT teams (U.S. state‑funded ACT programs). | N = 72 Medical diagnosis: SMI   - Schizophrenia spectrum disorder: 54.2% - Bipolar disorder: 22.2% - Major depressive disorder: 9.7% - Other: 4.2% - Not reported/refused: 9.7%   Age: Mean = 47.8 years, SD = 12.9 Sex: 52.8% men  Groups: N/A | - ACT users (diagnosed with schizophrenia, bipolar disorder or major depression) - High service use (frequent hospitalizations) - Functional deficits for independent living | Urban | N.D. | - Work-related self-efficacy (WSS-37) - BSI - Internalized stigma (ISMI) - Recent psychiatric hospitalization (past year, dichotomous variable) | Internalized stigma was found to be negatively associated with work-related self-efficacy, while recent hospitalization showed a positive association. Psychiatric symptoms did not have a significant relationship with work-related self-efficacy. The explanatory model indicated a small to moderate effect size. These findings suggest that stigma may pose a greater barrier to vocational integration than clinical symptoms. |
| Wilk et al. (2013); Canada | Observational. Prospective longitudinal study.  Assertive Community Treatment (ACT) — standard, high‑fidelity ACT teams operating under Ontario’s provincial ACT model. | N = 216 Medical diagnosis: SMI   - Schizophrenia: 58.8% - Schizoaffective disorder: 26.39% - Bipolar disorder: 14.81%   Age: Mean = 44.31 years Sex: 60.65% men  Groups:   - N participants who participated in the long-term follow-up (n = 191) - N participants who did not (n = 25) | - Primary diagnosis of severe psychosis (schizophrenia, schizoaffective disorder, severe bipolar disorder, or other major psychotic disorder) - Multiple comorbidities - High hospital utilization - Chronic illness - High level of needs Low functioning - Exclusions: primary diagnosis of alcoholism, substance use disorder, intellectual disability, or personality disorder (these were allowed as comorbidities) | Mixed | 2000-2009. 24-month follow-up | - ODPS: Overall Degree of Problem Severity - COLF: Current Overall Level of Functioning - COSR: Current Overall Strengths and Resources - Primary diagnosis - No. of comorbidities - Days of hospitalization in the last 5 years - Years since diagnosis. | Growth Mixture Modeling identified two trajectory classes: the majority exhibited low and stable performance without significant improvement (normative class), while a smaller group showed better baseline functioning and significant improvement (best evolution class). Predictors of membership in the normative group included having two or more comorbidities and a diagnosis of bipolar disorder compared to schizoaffective disorder. Most patients achieved successful community tenure without hospitalization or incarceration for two years. The average duration of illness was nearly two decades, with substantial hospitalization days over five years. |
| Wolff et al. (1997); USA | RCT (cost-effectiveness analysis)  Assertive Community Treatment (ACT) — standard ACT (one arm), plus a modified ACT variant with added community workers (second arm). | N = 85 Medical diagnosis: SMI   - Schizophrenia: 67.1% - Delusional/paranoid disorder: 3.5% - Psychosis not otherwise specified: 15.3% - Bipolar disorder: 14.1% - Major depression: 11.8%   Age: Mean = 33.6 years, SD = 10.3 Sex: 58.8% men  Groups:   - N ACT with Community Workers group (n = 35) - N ACT Only group (n = 28) - N Brokered Case Management group (n = 22) | - Severe Axis I psychiatric diagnosis - Homeless or at risk of homelessness - No recent history of serious offences (rape, homicide, assault) - Willingness to receive treatment and participate in the study | Urban | N.D. (18-month follow-up) | - Contacts with the program - Patient satisfaction - Days in stable housing (last 30 days) - BPRS - Service costs: mental health, physical health, housing, income and subsidies | ACT, both with and without community workers, improved program contact, patient satisfaction, and psychiatric symptoms. Although stable housing showed a positive trend in the ACT groups, no significant differences were found. Total costs over 18 months per patient did not differ significantly, but ACT with community workers was less expensive, and ACT reduced hospitalizations more effectively than brokered case management. |
| Wood et al. (1995); New Zealand | Quasi-experimental study with matched control group.  Assertive Community Treatment (ACT) — standard ACT (Madison/PACT‑derived model) integrated with general practitioners (shared‑care adaptation). | N = 118 Medical diagnosis: SMI Age:   - 20–29 years: 22% experimental group, 20% control group - 30–39 years: 32% experimental group, 32% control group - 40–49 years: 20% experimental group, 17% control group - 50–59 years: 19% experimental group, 20% control group - 60–65 years: 7% experimental group, 10% control group   Sex:   - Experimental group: 63% men; - Control group: 63% men   Groups:   - N intervention group = 59 - N control group = 59 | - Aged 18–65 years - Two or more psychiatric hospitalizations in the past two years - Or ongoing contact with at least two psychiatric services - Exclusion: severe cognitive impairment, primary diagnosis of substance or alcohol abuse, or measured IQ below 65 | Mixed | N.D. | - Days of hospitalization - Day hospital attendance - Time to first rehospitalization | The experimental group experienced a significant reduction in hospitalization days and a longer time to rehospitalization compared to the control group. Additionally, there was no compensatory increase in day hospital attendance. |
| Zavradashvili et al. (2010); Georgia | Observational. Prospective pre-post study.  Assertive Community Treatment (ACT) — standard ACT adapted for a low‑resource setting (Georgian ACT pilot). | N = 26 Medical diagnosis: SMI Age: Mean = 43.2 years, SD = 10.6 Sex: 50% men  Groups:   - N patients who participated in the long-term follow-up (n = 26) - N patients who did not (n = 4) | - Aged 18–65 years - Diagnosis of schizophrenia or schizoaffective disorder (ICD-10) - Basic life skills - Lack of social contacts - Lack of regular contact with community psychiatric services | Urban | March 2006 – December 2006 | - SAPS and SANS - Adherence to treatment (no / partial / complete) - Number of hospital admissions - Number of relapses - Access to social benefits (disability pension or state aid) - Number and type of contacts with the team - Estimated costs of the service | Significant decreases were observed in positive and negative symptoms as well as hospital admissions. Only a small number of patients required hospitalization. The intervention was feasible, sustainable, and clinically effective in reducing hospitalization and enhancing social and clinical contact. Despite methodological limitations, ACT is proposed as a valuable component of mental health reform in low-income countries such as Georgia. |
| Zhao et al. (2015); China | Quasi-experimental study with non-randomized control group.  Assertive Community Treatment (ACT) — standard ACT with culturally adapted, family‑integrated enhancements (China ACT pilot). | N = 31 Medical diagnosis: Schizophrenia Age: Mean = 31.7 years, SD = 6.9 Sex: 64.5% men  Groups:   - N ACT group = 15 - N Control group = 16 | Patients:   - Aged ≥18 years - DSM-IV diagnosis of schizophrenia - Significant functional impairment - High need for psychiatric services   Family caregivers:   - Aged ≥18 years - Living with the patient - Providing care for at least 3 months | Urban | 2011-2012. 6-month follow-up | Patients:   - PANSS - SDSS - PSP - UPSA-B   Caregivers:   - SCL-90 - FBIS - SSQ | The ACT group experienced no rehospitalizations or relapses, while the control group had several hospitalizations and relapses. Significant improvements in psychiatric symptoms were observed in the ACT group across total and subscale PANSS scores. Functional measures, including overall disability, personal and social functioning, and self-care, also improved significantly. No significant differences were found in social activities, capacity assessments, employment rates, or outcomes for family caregivers. |

| **Reference; Year; Country** | **Study type** | **Review objective** | **Population** | **Intervention / Control** | **Search date** | **Study Characteristics (Number, Design and Date Range)** | **Main results** |
| --- | --- | --- | --- | --- | --- | --- | --- |
| Abufarsakh et al. (2023); Australia | Systematic review | To evaluate the impact of ACT involvement on substance use outcomes among people with SMI | Individuals with SMI, such as schizophrenia, bipolar disorder, and major depression, receiving ACT services. | Intervention: ACT. Comparators: Usual care or other treatment conditions in controlled studies; pre-post comparison in cohort studies. | April 2021 | 29 (23 primary studies)   - 8 cohort studies - 15 RCTs   1997-2020 | Multiple studies reported reductions in substance use severity, though study quality varied. Moderate improvements were observed in stages of change, while effects on prevalence and remission were inconsistent. Some studies noted reductions in hospital days related to substance use. Higher fidelity to the ACT model was associated with better outcomes. |
| Burns (2010); United Kingdom | Meta-analysis | To analyze the effectiveness of ACT in reducing hospitalizations and to understand the variability of results through a systematic review and meta-regression of 64 trials. | Individuals with severe mental illness treated in community-based services. | Intervention: ACT (teams with high fidelity to the model). Control: Standard care, low-intensity case management, or CMHT teams. | 2000 | 64  RCTs  1988 - 2005 | Reductions in hospitalization were mainly influenced by differences in hospital use among control groups, with greater decreases observed where controls had higher baseline hospital use. Organizational fidelity to the ACT model was linked to reduced hospitalization, while fidelity in staffing and caseload was not. Well-organized CMHT teams may achieve similar outcomes at a lower cost, raising questions about the widespread implementation of high-fidelity ACT. |
| Burns et al. (2007); United Kingdom | Meta-analysis | To evaluate the effects of Intensive Case Management (ICM) in individuals with severe mental illness compared to standard care or non-intensive case management. Additionally, to assess whether fidelity to the ACT model and baseline hospital use influence the effects of ICM. | Adults (18–65 years) with SMI (schizophrenia, psychotic disorders, bipolar disorder, depression with psychotic symptoms) living in the community. | Intervention: Intensive Case Management (caseload ≤20). | January 2007 | 29  RCTs  1988 - 2005 | ICM is more effective at reducing hospital days when patients have a history of high hospital use. Team organization aligned with the ACT model is also associated with fewer hospital days, while fidelity to ACT staffing does not show a significant effect. The impact of ICM is smaller for patients with already low hospital use. |
| Coldwell et al. (2007); USA | Meta-analysis | To evaluate the effectiveness of ACT in the rehabilitation of homeless individuals with severe mental illness, compared to other models. | Homeless individuals with severe mental illness. | Intervention: ACT or ACT-based models. Control: Standard treatment. | 2003 | 10   - 6 RCTs - 4 observational studies   1992-2001 | In randomized controlled trials, ACT reduced homelessness and improved symptom severity compared to standard case management, though no significant differences were found in hospitalization rates. Observational studies reported even greater reductions in homelessness and symptom severity compared to baseline. However, results are limited by study heterogeneity and variable quality. |
| Dieterich et al. (2017); United Kingdom | Systematic review (+ meta-regression) | To evaluate the effects of ICM in individuals with severe mental illness compared to standard care or non-intensive case management. Additionally, to assess whether fidelity to the ACT model and baseline hospital use influence the effects of ICM. | Adults aged 18 to 65 with severe mental illness (schizophrenia, psychotic disorders, personality disorders) treated in community settings. | Intervention: Intensive Case Management (ICM) with fewer than 20 clients per case manager. Control: Standard care or non-intensive case management (more than 20 clients per case manager). | April 2015 | 40  RCTs  1985-2010 | ICM slightly reduces hospital days per month compared to standard care and improves retention in follow-up. Its effects on suicide, employment, and quality of life remain uncertain. Greater fidelity to the ACT model and higher baseline hospital use are linked to better outcomes in reducing hospitalization. |
| Goulet et al. (2022); USA | Meta-analysis | To assess the effectiveness of Forensic ACT in individuals with severe mental illness involved in the justice system, analyzing both forensic and health-related outcomes. | Adults with severe mental illness involved in the criminal justice system. | Intervention: FACT programs (hybrid model combining ACT with forensic rehabilitation). Control: Usual treatment or pre-intervention conditions. | February 2020 | 16   - 6 RCTs - 10 observational studies   1994-2018 | FACT significantly reduced jail days in forensic populations, demonstrating moderate effect sizes compared to both pre-post measures and control groups. Its effectiveness on health outcomes was limited, with no significant differences in hospitalization and increased outpatient visits. High heterogeneity and program variability were noted. The positive impact on justice-related outcomes was primarily driven by reduced incarceration time. |
| Herdelin et al. (1999); USA | Meta-analysis | To assess the effectiveness of the PACT model (Program of Assertive Community Treatment) compared to standard hospital or outpatient care in individuals with severe mental illness, using six key outcome indicators. | Individuals with severe mental illness (aged 18–65). Primary diagnoses of addiction, intellectual disability, and organic syndromes were excluded. Included populations such as homeless individuals and military veterans. | Intervention: PACT and similar community-based, interdisciplinary programs. Control: Traditional hospital-based or outpatient treatment. | N.D. | 19 RCT / CCT  1973-1997 | PACT reduced the number of hospitalizations, length of stay, and symptom severity while improving social functioning and patient satisfaction. Costs were generally lower with ACT, although variability was high and few studies were suitable for cost analysis. Statistical power was highest for patient satisfaction and lowest for symptom severity. Limitations included variability in outcome measures, small effect sizes for some indicators, and potential attrition bias. |
| Nordén et al. (2012); Sweden | Meta-analysis | To evaluate the effectiveness of the RACT model (Resource Group Assertive Community Treatment) compared to standard treatment in individuals with severe mental illness. | Individuals with severe mental illness (particularly psychosis), although other diagnoses were accepted. RACT was implemented following the Integrated Mental Health Care Workbook, ensuring that the Resource Group model was applied, with patients and their support networks actively participating in the therapeutic team. | Intervention: RACT (Resource Group Assertive Community Treatment; patient and support network actively participate in the therapeutic team). Control: Standard mental health care. | 2011 | 17   - 6 RCT studies - 11 observational studies   1980-2011 | The RACT model, which actively involves patients and their support networks in care, is effective for treating severe mental illness, especially psychosis. A meta-analysis of 17 studies, including both randomized and observational designs, demonstrated a large overall effect. Significant improvements were found in functioning and well-being, with moderate effects on symptoms. Positive outcomes were observed across various diagnoses, indicating broad applicability. Patient empowerment through active involvement appears to be a key factor in therapeutic success. No significant differences were identified based on study duration, design, or analysis type, and no publication bias was detected. |
| Randall et al. (2015); United Kingdom | Meta-analysis | To determine whether early intervention programs for psychosis (based on ACT) reduce hospital service use (hospital admissions and bed-days) compared to standard care. | Individuals with first-episode psychosis treated in early intervention programs. | Intervention: Early intervention programs with an ACT approach (multidisciplinary teams, home visits, case management). Control: Standard treatment or usual community care. | August 2013 | 15   - 3 RCTs - 6 historical control - 2 matched historical control - 3 cohort - 1 cohort studies with historical control   1996-2012 | The meta-analysis showed a significantly lower probability of hospitalization and a reduction in bed-days for the intervention group compared to standard care. However, high heterogeneity was observed across studies. While these programs are generally more effective than standard care, their impact varies depending on context and program quality. |
| Smith et al. (2007); Australia | Systematic review | To assess the available evidence from the last 10 years (since the Cochrane reviews of 1996 and 1998) on types of case management in mental health, focusing on outcomes and methodological quality. | Individuals with severe mental illness in community care settings; some studies included subgroups such as homeless persons or individuals with low intellectual functioning. | Intervention: Various forms of case management (ACT, ICM, strengths-based models, intensive supervision, generic models, outreach). Control: Standard care, conventional case management, or alternative models. | 2006 | 60   - 39 analytic studies - 21 descriptive studies   64  1974-2005 | ACT and ICM are more effective than standard case management in reducing hospital days and improving adherence, independence, treatment compliance, and patient satisfaction. However, results are inconsistent regarding symptoms, number of admissions, social functioning, and quality of life. There is limited evidence of impact on suicide, mortality, or unmet needs. Frequent methodological issues include poorly defined control and experimental groups, lack of power calculations, high heterogeneity, and absence of blinding. |
| Vanderlip et al. (2017); USA | Systematic review | To evaluate the impact of ACT programs on general medical outcomes, healthcare costs, and access to general medical care among individuals with severe mental illness. | Adults with severe mental illness; some studies included individuals who were homeless or at risk of homelessness. | Intervention: ACT or similar ICM models (with fidelity to the ACT model or equivalent structures). Control: Usual treatment. | November 2015 | 10   - 4 RCTs - 5 cohort - 1 case-control   1995-2015 | Half of the studies reported reduced emergency room use, and most found increased access to primary care. Most studies found no differences in total medical costs. Some studies evaluating quality of life or satisfaction reported moderate improvements. No clinical or mortality outcomes related to general medical conditions were reported. |
| Vijverberg et al. (2017); United Kingdom | Systematic review | To assess the effects of youth ACT on psychiatric symptom severity, overall functioning, and psychiatric admissions. | Children and adolescents (6–18 years) with severe mental illness, poorly engaged with community services. | Intervention: Youth-ACT (meeting ≥6 of the 9 core elements of the ACT model). No standardized control group across studies; designs include pre-post and RCTs. | August 2016 – March 2017 | 13   - 8 pre-post (no control group) - 4 RCTs - 1 quasi-experimental   2002 - 2016 | Youth-ACT shows promising effects including reductions in psychiatric symptoms (particularly psychotic and emotional symptoms), improvements in overall functioning, and decreases in psychiatric admissions and hospital days. Effects vary depending on diagnosis, and further randomized controlled trials are recommended. |

**Abbreviations:** ACT, Assertive Community Treatment; SMI, severe mental illness; SPMI, severe and persistent mental illness; RCT, randomized controlled trial; ED, emergency department; N.D., not described/not reported; NR, not reported; ICD, International Classification of Diseases; DSM, Diagnostic and Statistical Manual of Mental Disorders; MANSA, Manchester Short Assessment of Quality of Life; GAF, Global Assessment of Functioning; HoNOS, Health of the Nation Outcome Scales; BPRS, Brief Psychiatric Rating Scale; PANSS, Positive and Negative Syndrome Scale; CGI, Clinical Global Impression.

**Table S3. Core ACT characteristics and fidelity-related elements across included studies.**

| **Study (Year); Country** | **Ratio / Caseload** | **Multidisc. Team** | **In-vivo / Community Care** | **24/7 Availability** | **Duration / Continuity** | **Fidelity tool/score reported (DACTS/TMACT/Other)** | **Key Characteristics & "Nice to Haves"** |
| --- | --- | --- | --- | --- | --- | --- | --- |
| **Aagaard et al.** (2011); Denmark | 11.7:1 | Yes (psychiatrist + psychologist + work consultant + nurses/OT/social worker) | Yes | Yes(hotline) | Unlimited | McGrew fidelity schedule: 27/31 points = 87% | Key: High-fidelity “classic ACT” in rural Denmark + structured psychosocial add-ons (multifamily psychoeducation, social skills training, crisis homes, supported employment) |
| **Aagaard et al.** (2015); Denmark | 11–17:1  (South ~11.8, North ~11.0, Thy/Mors ~17.1) | Yes (psychiatrist + psychologist + work consultant + nurses/OT/social worker) | Yes | Yes (hotline) | Unlimited | McGrew (fidelity differed by team): 90.3% / 77.4% / 48.4%. | Key: Danish classic ACT teams implemented at regional level |
| **Aagaard et al.** (2017); Denmark | Low ratio (N.D) | Yes | Yes | Yes | Long-term | N.D. | Key: Classic ACT |
| **Ahrens et al.** (2007); USA | N.D. | Yes | Yes | Yes | Ongoing | N.D. | Key: PACT model applied to youth (15–21).  Nice to have: stronger family/foster care coordination, |
| **Albert et al.** (2017); Denmark | ≤12:1 (raised to 15:1 in the extended 5-year | Yes( psychiatrists, psychologists, nurses, social workers, physiotherapists, and vocational therapists) | Yes | No | N.D. | N.D. | OPUS Specialized Early Intervention Model.  Nice to have: Family intervention and social skills training. |
| **Arahanthabailu et al.** (2022); India | Case coordinators managed 4–5 clients each | **Yes(psychiatrist + psychiatric social worker + nurse + trainee postgraduate staff)** | Yes | No (Phone follow-up) | N.D. | DACTS: 2.9 (low fidelity) | Adaptation: M-ACT modified for resource-limited settings. |
| **Aubry et al.** (2015); Canada  **Aubry et al.** (2016); Canada  **Aubry et al.** (2019); Canada | 10:1 | **Yes** | Yes | Yes | N.D. | N.D. | Key: Housing First with ACT-based intensive community support for homeless adults  Nice to have: Integrated |
| **Baier et al.** (2013); Switzerland | 10:1 | Yes (child psychiatrist + social worker + nurse) | Yes | No | Time-limited | N.D. | Assertive and Intensive Mobile Team for Adolescents (AIMA)  Key: Early tailored assertive community case management for adolescents (13–18) + family involvement; |
| **Bertelsen et al.** (2008); Denmark | 10:1 | **Yes** | Yes | No (Mon–Fri 8am–5pm) | Time-limited (2 years) | ACT fidelity index reported: 70% fidelity | OPUS model: Early-intervention program Key: combining ACT + psychoeducational family treatment + social skills training |
| **Bodén et al.** (2010); Sweden | 10–20:1 | **Yes (psychiatrist, psychologist, nurse, social worker, mental health assistant, and occupational therapist.)** | Yes | No | N.D. | Only mentions Dartmouth criteria and modifications | mACT (modified Assertive Community Treatment)  Key: first-episode psychosis with high assertiveness  Nice to have: family psychoeducation. |
| **Bond et al.** (1990); USA | 10:1 | **Yes** | Yes | N.D. | Continuous | N.D. | Key: State-funded classic ACT. Stronger client engagement/ |
| **Bond et al.** (1991); USA | **10:1–15:1** | **Yes** | Yes | N.D. | Continuous | N.D. | Key: ACT for young adults with SMI + substance abuse |
| **Bond et al.** (1994); USA | ≤15:1 | **Yes** | Yes | Yes | Continuous | DACTS: 3.6–4.2 | Key: State-funded classic ACT. Money-management support, and ongoing fidelity monitoring/feedback. |
| **Bonsack et al.** (2005); Switzerland | 10:1 | **Yes (2.8 FTE nurses, 1 FTE social worker, and 1.8 FTE psychiatrists)** | Yes | No (Mon-Fri) | Time-limited | N.D. | Key: Time-limited ACT. Approach focused on engagement, motivation, and social networks. |
| **Botha et al.** (2010); South Africa  **Botha et al.** (2014); South Africa | 35:1 | Yes (psychiatrist + social worker + psychiatric nurse) | Yes | No | N.D. | DACTS: 3.1 (moderate fidelity) | Adaptation: Modified ACT for low/middle-income countries; high caseload. |
| **Calsyn et al.** (1998); USA | 10:1 | Yes | Yes | Yes | Unlimited | N.D. | ACT – Homeless population  Key: High fidelity to community-based care. |
| **Carton et al.** (2010); USA | 10:1 | Yes | Yes | Yes | Continuous | DACTS:  ≈ 4.0/5 (high fidelity) | ACT – Formerly homeless population  Key: Services delivered directly by the team (not outsourced). |
| **Chandler et al.** (1999); USA  **Chandler et al.** (2002); USA | 6.1-10:1 | Yes | Yes | Yes | Yes | Community Program Philosophy Scale (CPPS): program classified as ACT‑consistent (no Dartmouth scores) | STRIDES Model. Nice to have: Planned transition to lower intensity (Step-Up). |
| **Chow et al.** (2011); Canada/Japan | 10:1 | Yes | Yes | Yes | Continuous | Dartmouth ACT Fidelity Scale: Yes (Toronto 4.5/5; Japan 3.9/5; good fidelity) | Nice to have: Cultural assessment, bilingual staff (Canada); high family involvement (Japan). |
| **Clark et al.** (1998); USA | 12:1 | Yes | Yes | N.D. | N.D. | N.D. | Nice to have: Direct substance abuse intervention; stage-based therapy groups. |
| **Clarke et al.** (2000); USA | 10:1 | Yes | Yes | Yes | Unlimited | Dartmouth ACT Fidelity Scale: Yes (scores N.D.) | Nice to have: Peer providers on the team. |
| **Clausen et al.** (2015); Norway  **Clausen et al.** (2016); Norway  **Clausen et al.** (2016); Norway  **Clausen et al.** (2020); Norway | N.D. | Yes | Yes | Yes | Continuous | TMACT: Yes (mean fidelity ranged from low–moderate to moderate–high over time) | Nice to have: Integrated dual diagnosis and vocational specialists. |
| **Coetzee et al.** (2022); South Africa | 30–35:1 | Yes (: psychiatrist, medical officer, social worker, psychiatric nurses; access to psychology/OT, dual‑diagnosis, PSR, SUSD) | Yes | No | N.D. | N.D. | Adaptation: Access to psychosocial rehabilitation and SUSD centers. |
| **Commander et al.** (2005); UK | 10:1 | Yes (psychiatrist, nurses, social workers; psychology/OT/support as available) | Yes | Yes | Continuous | Set up with high PACT fidelity per policy; no formal fidelity scale reported. | Key: High fidelity to PACT. |
| **Cusack et al.** (2010); USA | Low ratio (N.D.) | Yes | Yes | Yes | Continuous | DACTS: scores 4.5–4.6 (high fidelity) | FACT (Forensic): Nice to have: Active judicial supervision and coordination with correctional system. |
| **Dahlan et al.** (2013); Malaysia | 40:1 | Yes | Yes | No | Continuous | N.D. | Adaptation: High caseload, needs-focused services adapted to local context. |
| **Daubney et al.** (2021); Australia | 8:1 | Yes (psychology, social work, mental health nursing, and therapy, supported by a Consultant Psychiatrist and a Team Leader) | Yes (Parks) | No | Limited (maximum of 2 years) | N.D. | Assertive Mobile Youth Outreach Service (AMYOS) for adolescents  Nice to have: Youth-focused; training in Metallization-Based Therapy (MBT). |
| **de Bruijn et al.** (2023); Netherlands | 200/team | Yes (psychiatrist, psychologist, employment specialist, social worker, peer counselor, nurses/case managers/SPN, others) | Yes | N.D. | N.D. | N.D. | Flexible TAC  Key: recovery-oriented and discharge is an explicit final  Nice to have: Digital care and focus on functional recovery and autonomy. |
| **Dekker et al.** (2002); Netherlands | 13:1 | Yes (project manager + psychiatrist part-time + 3 case managers/social psychiatric nurses + 5 rehabilitation workers/psychiatric nurses) | Yes | No | N.D. | N.D. | Key: Dutch ACT-style intensive home treatment with high community contact + practical in-vivo support; Working Apart Together model (case manager + rehab worker per patient |
| **Dincin et al.** (1993); USA | 10:1 | Partial (core team of 6 generalist practitioners; plus psychiatric consultant for intensive medication monitoring 7 hrs/week; hospital liaison role rotated among staff) | Yes | N.D. | N.D. | N.D. | Nice to have: Psychiatric consultant available for home visits; hospital liaison. |
| **Dixon et al.** (1997); USA | N.D. | Yes | Yes | N.D. | N.D. | N.D. | Key: homeless adults, voluntary care. |
| **Doré-Gauthier et al.** (2020) ; Canada | 10–15:1 | Yes (psychiatrists, nurses, social workers, occupational therapists) | Yes | N.D. | N.D. | N.D. | Intensive Assertive Community Intervention Team – Homeless Youth with Psychosis and Substance use (IACIT-HYPS) Nice to have: Specialized ACT-style intensive outreach added to early psychosis services for homeless youth with SUD; housing support. |
| **Drake et al.** (1998); USA | 10:1 | Yes (psychiatrist + nurses + case managers; team approach with shared caseload; staff with substance use treatment expertise) | Yes | Yes | N.D. | N.D. | Nice to have: Integrated dual diagnosis treatment. |
| **Drukker et al.** (2011); Netherlands  **Drukker et al.** (2014); Netherlands | N.D. | Yes | Yes | N.D. | Continuous | DACTS: Yes (good fidelity reported) | FACT (Function ACT)  Key: Flexible care (transition between levels without changing teams). |
| **Essock et al.** (1998); USA  **Essock et al.** (2006); USA | 10-15:1 | Yes (nurses and at least one half-time psychiatrist + staff coverage for shifts/weekends; “mini-team/shared caseload” organization) | Yes | Yes | N.D. | N.D. | Key: outreach/community delivery + stage wise integrated substance treatment. |
| **Fam et al.** (2007); Singapore | N.D. | Yes (psychiatrist, medical officer, medical social worker, 6 community psychiatric nurses, occupational therapist) | Yes | N.D. | N.D. | N.D. | Key: Mobile multidisciplinary, in-vivo care focused on the patient’s environment, with strong family/caregiver involvement. |
| **Fekete et al.** (1998); USA | 10:1 | Partial/limited (rural teams were small: 2 case managers) | Yes | No | Unlimited | McGrew 17-item ACT Fidelity Scale (0–1). Rural teams mean 0.35 (low fidelity) vs urban benchmark 0.61. | Adaptation: Rural ACT with small teams of 2 professionals. |
| **Firn et al.** (2013); UK  **Firn et al.** (2016); UK | N.D. | Yes | Yes | No | N.D. | N.D. | Key: Flexible ACT within CMHT (step-up/step-down + shared caseload)  Adaptation: Comparison between Assertive Outreach and Flexible ACT (FACT). |
| **Gold et al.** (2012); USA | 9-11:1 | Yes (RN, psychologist, social workers, SUD specialists, OT, vocational specialists; + part-time psychiatrist 0.2 FTE) | Yes | N.D. | Long-term | DACTS: 4.4 (1996), 4.4 (1997), 4.5 (1998) (moderately high/high fidelity) | Key: Direct mentorship from PACT founders. Nice to have: Vocational specialists. |
| **Hackman et al.** (2009); USA | Low ratio(N.D) | Yes | Yes | Yes | N.D. | Mentions DACTS conceptually (N.D.) | Key: Annual assessment for step-down referral. |
| **Hambridge et al.** (1994); Australia | 9–10:1 | Yes (nurses + social worker + OT + psychologist; psychiatrist/registrar cover) | Yes | Partial: Mon–Fri 8:00–21:00; weekends 9:00–17:00; after-hours covered by Extended Hours Team/24h on-call) | N.D. | N.D. | Mobile Community Management Team (MCMT)  Nice to have: High integration with housing services and social support + family interventions + early relapse detection |
| **Hamernik et al.** (1999); Australia | 7:1 | Yes (psychiatrist + 2 psychiatric nurses + social worker + psychologist) | Yes | Partial: **7-day service** + weekday extended hours (8:30–18:00); **24-h mental health service** covers outside hours | N.D. | N.D. | Key: Very intensive ACT; weekly team meetings; primary + backup worker. Support with hygiene, nutrition, and basic life skills. |
| **Hamilton et al.** (2015); UK | Low ratio (N.D.) | Yes | Yes | N.D. | N.D. | N.D. | **Assertive Outreach Teams**  Adaptation: Use of mental health nurses as primary care coordinators. |
| **Hansen et al.** (2023); Denmark | 10:1 | Yes | Yes | N.D. | N.D. | N.D. | OPUS Model.  Key: modified ACT + **Social Skills Training** + psychoeducation + family involvement |
| **Harvey et al.** (2011); Australia/UK | 6.3:1 (Melbourne) / 9.5:1 (UK) | Yes | Yes | Yes (Melbourne) | N.D. | DACTS total: Melbourne 3.7; London Cluster A 3.6, Cluster B 3.4, Cluster C 2.9 | Key: Shared caseload and high psychiatrist involvement. |
| **Hastrup et al. (2013)**; Denmark | 10:1 | Yes (psychiatrist, nurse, psychologist, social worker, OT, secretary; plus vocational guide) | Yes | N.D. | N.D. |  | OPUS Model.  Key: enriched ACT + family psychoeducation + social skills training (FEP). |
| **Hastrup et al. (2015)**; Denmark | 10:1 | Yes (**7 case managers**: 5 nurses + SW + OT) | Yes | Partial (Hotline) | N.D. | McGrew’s fidelity schedule: 27/31 points (87%) | Key: Rural ACT + multi-family groups + social skills training + crisis centers + supported employment. |
| **Herinckx et al. (1997)**; USA | 10:1 | Yes (shared psychiatrist + nurse practitioner + clinical director; case manager team structure | Yes | Yes | Unlimited | N.D. | Adaptation: Teams composed of peer providers vs. professional teams. |
| **Horiuchi et al. (2006)**; Japan | N.D. | Yes (nurses, PSW, psychologists + full-time psychiatrist) | Yes | N.D. | N.D. | DACTS: 4.1/5 (excluding dual-diagnosis items) | ACT-J: Key: Focus on self-care, housing, and social skills. |
| **Huguelet et al. (2012)**; Switzerland | 10:1 | Yes (3 FTE nurses, 1 social worker, 1 psychologist, 0.5 FTE psychiatrists) | Yes | No | N.D. | Dartmouth: 3.82 (moderate implementation | Key: Focus on therapeutic engagement with family and caregivers. |
| **Ito et al. (2009)**; Japan  **Ito et al. (2011);** Japan | N.D. | Yes | Yes | Yes | N.D. | DACTS = 4.1/5 (high fidelity) | Key: ACT-J with 24/7 coverage + outreach; high fidelity |
| **Jerrell (1995)**; USA | 15–20:1 | Yes (multidisciplinary + psychiatrist + supervisor; daily briefings) | Yes | N.D. | N.D. | N.D | Key: PACT adaptation including  multidisciplinary continuous-care team + daily meetings + direct services (med monitoring/SUD/vocational/crisis). |
| **Jones (2002)**; UK | 15:1 | Yes (team manager nurse; nurse practitioners; social worker; residential support workers; secretary) | Yes | Partial: Mon–Fri 09:00–17:00 + links to 24-h crisis response service | N.D. | Mentions Dartmouth ACT scale conceptually, no score | Key: Daily meetings and practical support (shopping, bill payments). |
| **Kane et al. (2004)**; USA | N.D. | Yes (typical PACT team + Advanced Practice Nurses + consumer peer providers) | Yes | N.D. | N.D. | N.D. | NPACT:  Key: PACT enhanced with advanced practice psychiatric nurses + peer providers (focus on physical health + symptom management). |
| **Karow et al. (2012–2020)**; Germany  **Karow et al. (2020)**; Germany | 15-25:1 | Yes (consultant psychiatrist + psychiatrists + psychologists + nurses; psychotherapy training) | Yes | Yes | Unlimited | N.D. | Hamburg ACCESS model  Key: Manualized IC-TACT within integrated care (high-intensity outreach + no-dropout).  Nice to have: Staff with advanced psychotherapeutic training (CBT/psychodynamic). |
| **Kenny et al. (2004)**; USA | 10–15:1 | Yes | Yes | Yes | N.D. | N.D. | Homeless ACT.  Nice to have: Paraprofessionals for recreational activities. |
| **Killaspy et al. (2006)**; UK  **Killaspy et al. (2009)**; UK  **Killaspy et al. (2014)**; UK | 12:1 | Yes | Yes | 8:00–20:00 | Unlimited | N.D. | REACT Model.  Key: No-dropout policy and long-term engagement. |
| **Kim et al. (2015)**; South Korea | 5:1 | Yes (psychiatrist lead; team leader; nurse; social worker) | Yes | Yes | N.D. | N.D. | Key: Very low ratio; focus on work environment and family involvement. |
| **Kortrijk et al. (2010)**; Netherlands  **Kortrijk et al. (2012)**; Netherlands  **Kortrijk et al. (2010–2014)**; Netherlands | N.D. | Yes | Yes | N.D. | N.D. | DACTS total 3.5 (2.9–3.8) | Key: ACT teams; outcomes tracked via **Routine Outcome Monitoring** (ROM) |
| **Kortrijk et al. (2019)**; Netherlands | N.D. | Yes | Yes | N.D. | N.D. | N.D. | Flexible ACT Key: step-up/step-down, shared caseload during instability. |
| **Kreindler et al. (2010);** Canada | N.D. | Yes | Yes | N.D. | N.D. | High-fidelity ACT service (N.D.) | Key: High-fidelity ACT linked to improved housing stability after ~6 months and increased independent living |
| **Lafave et al. (1996)**; Canada | N.D. | Yes (psychiatrist, nurses, social workers, vocational counselor, support worker/peer survivor) | Yes | Yes | N.D. | N.D. | Nice to have: Peer support worker with lived experience on the team + living-skills support |
| **Lambert et al. (2010);** Germany | Max 15:1 | Yes (consultant psychiatrist, psychiatrist, psychologists, psychiatric nurse, social worker; psychosis experts with psychotherapy training) | Yes | Yes | Unlimited | **DACTS: 4.5/5 (good** fidelity) | ACCESS trail  Key: ACT embedded in integrated psychosis care; includes individual + family psychotherapy. |
| **Lamberti et al. (2017)**; USA | N.D. | Yes (high-fidelity ACT team with “criminal justice–savvy staff”) | Yes | N.D. | Continuous | DACTS: baseline 4.75 (high fidelity).  Other (FACTS): 4.69 and 4.61 (6 and 15 months; high fidelity). | FACT (Forensic)  Key: high-fidelity ACT + targets criminogenic risks + legal leverage (judicial monitoring) + mental health–justice collaboration.  Nice to have: Weekly judicial supervision and justice collaboration. |
| **Lang et al. (1999)**; USA | N.D. | Yes (nurses, psychiatrists, social workers, psychologist + paraprofessional mental health workers; includes a peer counselor) | Yes | N.D. | N.D. | N.D. | Key: Team-based ACT with peer counselor. Crisis intervention and intensive family/group therapies. |
| **Latimer et al. (2020)**; Canada | 8:1 / 10:1 | Yes (ACT team + psychiatrist; housing specialists supporting rent supplements/housing retention) | Yes | N.D. | N.D. | Other: fidelity assessments of local implementation (2 time points: ~1 year and 3 years). N.D. | Nice to have: Scattered-site Housing First + ACT with rent supplements + housing specialists; intensive recovery-oriented support. |
| **Lee et al. (2015)**; Hong Kong, China | 15:1 | Yes (consultant psychiatrist lead; psychiatrists, psychologists, community psychiatric nurses, social workers, occupational therapists) | Yes | Yes | N.D. | Similar to Dartmouth ACT Fidelity Scale (N.D.) | Key: Violence assessment and same psychiatrist follow-up throughout. |
| **Lehman et al. (1997)**; USA  Lehman et al. (1999); USA | 10–12:1 | Yes (program director, full-time psychiatrist/medical director, 6 clinical case managers (SW/psych nurses/rehab counselors), 2 consumer advocates, secretary, part-time family outreach worker, part-time nurse practitioner) | Yes | Yes | Unlimited | N.D. | Key: ACT-based ACT for homeless SMI  Nice to have: Consumer advocates and nurse practitioner for medical care. |
| **Liem et al. (2013);** Hong Kong, China | 15:1 | Yes (consultant psychiatrist lead; 7 full-time case managers [nurses/OT]; part-time psychologist, medical social worker, 2 resident psychiatrists, supervising consultant) | Yes | Yes | N.D. | N.D. | Key: ACT for frequent readmitters |
| **López-Santiago et al. (2012)**; Spain | N.D. | Yes (nurse + nursing assistant + part-time social worker + psychiatrist + clinical psychologist) | Yes | No | Unlimited | N.D. | Key: Spanish ACT-style program, assertive engagement/continuity; medication adherence + crisis support; community-oriented (but no full 24/7) |
| **Low et al. (2013)** Singapore | Low caseload (N.D.) | Yes (doctors + community psychiatric nurses + social workers + occupational therapists) | Yes | No | N.D. | N.D. | **Key:** ACT-modeled multidisciplinary outreach |
| **Luo et al. (2019)**; China | N.D. | Yes (psychiatrist team leader + part-time psychiatrist + part-time psychiatric nurse + 2 psychologists + 3 social workers + part-time employment specialist) | Yes | N.D. | N.D. | TMACT: 3.8/5 (low–moderate fidelity); revised 4.1/5 after removing 6 culturally less relevant items | Key: Key: culturally adapted ACT + monthly family psychoeducation.  Nice to have: Recovery-oriented care with specialized employment specialists. |
| **Mantzouranis et al. (2019)**; Switzerland | N.D. | Yes (psychiatrists, nurses, social workers) | Yes | N.D. | Unlimited | N.D. | Key: Youth ACT with no-dropout policy, high visit frequency (up to 10×/week), intensive community outreach + family involvement; flexible interventions (early intervention/transition/support for hard-to-engage). |
| **Manuel el al. (2011)** USA | 1:10 | Yes | Yes | Yes | N.D. | N.D. | Key: ACT used to deliver integrated dual-disorder treatment (psychosis + SUD);  Nice to have: structured stagewise dual-diagnosis interventions |
| **Marquant et al. (2018b)** ; Belgium | Low caseload (N.D.) | Yes (psychiatrists, social workers, psychiatric nurses, vocational specialist, expert service user; part-time team leader; embedded psychiatrists) | Yes | Yes | Continuous | Other (study-created fidelity scoring): scored presence of ACT elements (0–6) + forensic rehab elements (0–2); ForACT group mean 5.95/6 ACT elements and 2/2 forensic elements | Forensic ACT. Nice to have:  Key: integrated SUD + vocational support; structured risk assessment/management and close coordination with justice/probation. |
| **McCrone et al (2009);** UK | 12:1 | Yes | Yes | N.D. | Long-term | N.D. | REACT  Key: assertive engagement; long-term attempts to maintain engagement; team approach; frequent team meetings; staff deliver skills internally (not external agencies) |
| **McFarlane et al. (1996)**; USA | N.D. | Yes | Yes | N.D. | Long-term | N.D. | Nice to have: Multifamily psychoeducational groups (biweekly). |
| **McGrew et al. (1995)**; USA | 8–12:1 | Yes | Yes | N.D. | Continuous | Fidelity monitored via statewide coordinator; no formal score reported. | Key: Ongoing training and supervision by state coordinator. |
| **McGuire et al. (2004)**; USA | N.D. | Yes | Yes | N.D. | N.D. | N.D. | Adaptation: homeless individuals with criminal justice involvement |
| **Meisler et al. (1997)**; USA | Low caseload (N.D.) | Yes psychiatrist 0.5 FTE, MSW leader, 2 nurses, 7 MS/BS staff (MH + substance use + vocational) | Yes | Yes | Long-term | N.D. | Training in Community Living (TCL) Model. Key: Integrated substance use care with no abstinence requirement. |
| **Mohamed et al. (2010)**; USA  Mohamed  (2016); USA | Low caseload (N.D.) | Yes (psychiatric, medical, rehabilitation, and social support roles) | Yes | N.D. | Long-term | High fidelity to ACT on formal scales (N.D.) | Veterans-only national ACT‑like program (VA MHICM). |
| **Morrisey et al. (2013)** USA | Low caseload (N.D.) | Yes | Yes | N.D. | Long-term | TMACT (high fidelity: no numerical score reported) | Key: High‑fidelity, recovery‑oriented ACT; |
| **Morse et al. (1997)**; USA | ~10:1 | Yes | Yes | N.D. | Unlimited | N.D. | Key: homeless‑focused outreach, and prioritized housing/benefits.  Nice to have: Assigned community workers. |
| **Mowbray et al. (1997)**; USA | N.D. | Yes (a master’s-level team leader, an occupational or recreational therapist, a nurse, five bachelor’s-level or paraprofessional staff advocates, plus psychiatric support when needed) | Yes | Yes | Unlimited | N.D. | Key: Harbinger model, Classic early PACT replication. |
| **Nielsen (2021)**; Denmark | 20-30:1 | Yes | Yes | N.D. | N.D. | FACT Fidelity Scale assessed 9 months after implementation; 4 teams “good fidelity”, 1 team insufficient (no total score stated in main text) | Flexible ACT  Key: Flexible intensity via FACT board (step-up/step-down). |
| **Nielsen (2023)**; Denmark | FACT: 20-30:1  ACT: 15-15:1 | Yes  FACT: nurses, social workers, psychiatrists, psychologists, occupational therapists, peer workers. ACT: nurses, social workers, psychiatrists, psychologists, occupational therapists | Yes | N.D. | N.D. | N.D. | Flexible ACT  Key: flexible step-up/step-down model: stable patients receive individual case management with higher caseload (20–30); during deterioration/crisis care is stepped up to shared team-based ACT support. |
| **Nieves et al. (2002)** USA | 10:1 | Yes (psychiatrist + nurse + generalist staff; no dedicated substance abuse specialist / vocational specialist) | Yes | Yes | N.D. | PACT fidelity instrument (brief version) used to assess model fidelity (no total numeric score reported) | Key: Mini-teams/shared caseload; daily planning/meetings; staff travel in pairs (safety); max intake 3/month |
| **Nishio et al. (2012)**; Japan | 10:1 | Yes (nurses, social workers, occupational therapists + team leader; + part-time employment staff; + team psychiatrist; + program assistant) | Yes | Yes | N.D. | N.D. | ACT Japan. Key: Twice-daily team meetings; shared team approach; broad practical support (med delivery, ADL support, housing/food, employment support, leisure skills) |
| **Nordentoft et al. (2010);** Denmark  **Nordentoft et al. (2015);** Denmark  **Petersen et al. (2005);** Denmark  **Petersen et al. (2007);** Denmark | 10:1 | Yes (psychiatrist, psychologist, nurses, OT, social worker; vocational/education support) | Yes | No | Time-limited (2 years) | ACT fidelity index ~70% (lower mainly due to lack of 24h coverage / intensity criteria) | OPUS Model. Key: Modified ACT for first-episode psychosis + family involvement (psychoeducation/multi-family) + structured social skills training. |
| **Nugter et al. (2016)** Netherlands | N.D. | Yes (ACT team includes psychiatrist, case managers, psychologist, peer specialist, supported employment specialist) | Yes | Yes | Continuous | FACT fidelity scale (FACTs, 60 items, 1–5); total scores improved to 4.2 / 4.3 / 3.6 at 2 years (good–excellent) | Key: Flexible step-up/step-down intensity; stable phase = individual case management, crisis phase = shared caseload ACT; daily review via FACT board. |
| **Pérez de León et al.** (2011); USA | N.D. | Yes (licensed master’s clinician + 3 case workers + 2 outreach workers + RN + psychiatrist + benefits case worker + employment specialist when available) | N.D. | N.D. | Time-limited (up to 12 months) | DACTS (28 items); overall mean fidelity 3.59/5 (moderate fidelity) | Key: ACT integrated with the IT (Integrated Treatment) model. Nice to have: Cultural emphasis and specialized staff (public benefits caseworker and employment specialist). |
| **Pope et al.** (2014); Canada | N.D. | Yes (social work, rehabilitation, counselling, nursing, psychiatry) | Yes | N.D. | N.D. | DACTS used; high model fidelity (score not specified) | Key: Rural ACT; dual-diagnosis focus (high concurrent substance use); strong instrumental support (transport/errands) + med adherence support |
| **Poremski et al.** (2016); Canada | N.D. | Yes (specialised multidisciplinary mobile teams) | Yes | N.D. | Time-limited (trial period) | N.D. | Nice to have: Housing First + ACT (High Needs): scattered-site housing with rent supplements (≤30% income on rent); |
| **Razali et al. (2015)**; Malaysia | 30:1 | Yes | Yes | No | N.D. | N.D. | Adaptation: higher caseload, weekend call center instead of home visits. |
| **Rezansoff et al.** (2017); Canada | Low caseload (unspecified) | Yes | Yes | Yes | N.D. | N.D. | Key: Intensive community-based ACT support. Nice to have: Housing First (scattered-site private-market housing) + weekly substance-use support. |
| **Rohenkohl et al. (2022);** Germany  **Schöttle et al. (2018);** Germany  **Schöttle et al. (2019);** Germany  **Schröter et al. (2022);** Germany | 15-25:1 | Yes (psychiatrists, psychologists, nurses, social worker) | Yes | Partial/Yes (extended hours + 24h crisis telephone + 24h emergency service) | Unlimited | DACTS yearly; baseline 4.5; range 4.2–4.6 (good fidelity) | Hamburg ACCESS Model. Nice to have: Group and family psychotherapy. |
| **Salyers et al. (2011)**; USA | N.D. | Yes (psychiatrist, nurse, vocational specialist, substance abuse specialist; peer specialist involved) | Yes | Yes | N.D. | N.D. | Nice to have: Complemented by Illness Management and Recovery (IMR) modules. |
| **Salkever et al.** (1999); USA | Low ratio (varied 6.5-10.3:1) | Yes (Team approach) | Yes (Intensive) | Yes | N.D. | N.D. | Key: PACT model with shared caseload and intensive community care. |
| **Santos et al. (1993)**; USA | ~10-11:1 | Yes (4 nurses, 6 social workers, 0.5 psychiatrist | Yes (75%) | Yes | Unlimited | N.D. | Key: Controlled admissions (4–6/month); financial management support. |
| **Siskind et al. (2009)**; USA | 8-6:1 | Yes (team leader, master-level clinician(s), RN, substance abuse counselor, peer support, community support; psychiatrist consultation hours) | Yes | Partial/external: rural NC shared some overnight/weekend phone contact with co-located mental health services. | Unlimited | N.D. | Key: Urban MA closer to ACT fidelity (daily meetings; vocational + housing specialist). Nice to have: Peer support integration (different roles across sites). |
| **Siskind et al. (2021)**; Australia | 12:1 | Yes (team leader, 3 OTs, neuropsychologist, 2 social workers, 4 nurses, half-time psychiatry consultant + full-time psychiatry registrar) | Yes | N.D. | Time-limited | N.D. | MIRT. Nice to have: Hybrid ACT with rehabilitation-focused psychosocial programs; time-limited and discharge-oriented (“graduation” model). |
| **Slade et al. (2013)**; USA | 12:1 | Yes (team leader + nurses + psychiatrist + substance abuse specialist) | Yes | Yes | Unlimited | N.D. | Adaptation: ACT model adapted to the VA (veterans) system. |
| **Smith et al. (2010)**; USA | N.D. | Yes (forensic psychiatrists, nurses/RNs, counselors, social workers, recreational therapists, addictions counselors; aftercare case managers on-site daily) | Yes | Yes | Continuum model | N.D. | Key: Forensic continuum-of-care model (secure residential rehabilitation - step-down levels - conditional release). Nice to have: Court/legal monitoring with ACT-based community aftercare. |
| **Somers et al.** (2013); Canada | N.D. | Yes | Yes | Yes | Unlimited | N.D. | Key: Intensive community-based intervention. Nice to have: Integrated with Housing First; scattered-site housing and weekly substance use support. |
| **Sood et al. (2014)**; UK | N.D. | Yes | Yes | N.D. | Unlimited | DACTS used (twice):  March 2004: DACTS 4.4 (high fidelity)  July 2008: DACTS 4.2 (high fidelity) | Key: High-fidelity UK ACT team |
| **Sood et al. (2017)**; UK | N.D. | Yes (specialist psychosis/recovery teams delivering FACT; includes psychiatry + psychology input) | Yes | Yes | N.D. | N.D. | Key: Flexible step-up/step-down model with shared caseload during crises/high-need periods. |
| **Stein et al. (1999)**; USA | N.D. | Yes | Yes | Yes | N.D. | N.D. | Key: Original PACT-based model; continuous monitoring. |
| **Stobbe et al. (2014)**; Netherlands | 10:1 | Yes (substance-abuse specialist, rehabilitation worker, social worker, psychiatric nurse, somatic care nurse, community mental health nurse, psychiatrist (elderly specialist) | Yes | No | Unlimited | DACTS total mean: 3.6 (moderate fidelity). | Key: ACT-E (Elderly): Adaptation: Specialized geriatric care team. |
| **Svensson et al. (2018)**; Sweden | N.D. | Yes | Yes | N.D. | Flexible follow-up | Flexible ACT Fidelity Scale used; included teams had ≥3.5/5 (good fidelity) | Adaptation: Based on Dutch FACT model. Flexible ACT “board”: shared caseload + daily team discussion during intensive phases; continuity maintained by returning to individual case management once stabilized. |
| **Sytema et al. (2007)**; Netherlands | 10:1 | Yes | Yes | No | Unlimited | DACTS: 3.8 (team structure) and 3.9 (team organization); later 4.1 (organization) after adding client-worker. | Key: shared caseload with daily + weekly team meetings; office-hours ACT with centralized crisis support. |
| **Sytema et al. (2014)**; Netherlands | N.D. | Yes | Yes | N.D. | N.D. | DACTS:  ACT Plus team: 4.0 / 3.3  Standard ACT team: 3.6 / 3.3 | ACT Plus: Nice to have: ACT Plus added evidence-based interventions delivered independently from ACT team (psychoeducation, family intervention, IPS, CBT). |
| **Tempier et al.** (2012); UK | N.D. | Yes (Specialized: Early intervention) | Yes (Patient's environment) | N.D. | Flexible | N.D. | Key: Early Intervention-Focused ACT. Nice to have: Specific focus on family involvement. |
| **Thoegersen et al. (2019)**; Denmark | 10:1 | Yes | Yes | No (Monday-Saturday) | Unlimited | IFACT (Index of Fidelity to ACT). Fidelity in both ACT teams: 91.4% ( | Key: “No drop-out” engagement policy with frequent (up to daily) team meetings. |
| **Tibbo et al. (1999)**; Canada | < 20:1 | Yes | Yes | Yes | Unlimited | N.D. | Key: High contact frequency and mobile availability. |
| **Tibbo et al. (2001)**; Canada | Low ratio (N.D.) | Yes | Yes | Yes | Unlimited | N.D. | Key: High contact outreach and mobile support. |
| **Tinland et al. (2020)**; France | 10:1 | Yes (social worker, nurse, doctor, psychiatrist, and peer worker) | Yes | N.D. | Unlimited | N.D. | Nice to haves: ACT-based intensive support within Housing First. |
| **Tomar et al. (2020)**; USA | 10:1 | Yes | N.D. | N.D. | N.D. | N.D. | Key: High fidelity to the U.S. national ACT model. |
| **Udechuku et al. (2005)**; Australia | 7:1 | Yes (9 clinicians: six community psychiatric nurse/case managers, one occupational therapist/case manager, half-time psychiatry registrar, and consultant psychiatrist input) | Yes | No | N.D. | N.D. | Key: Focus on basic life skills and psychosocial integration. |
| **Valenstein et al.** (2013); USA | Low ratio (N.D.) | Yes | Yes | N.D. | N.D. | N.D. | Key: Team-based approach, low patient-to-staff ratio, and individualized services in Veterans Affairs (VA) settings. Nice to have: Practical medication management (including medication delivery) and adherence support; high contact intensity defined as ≥42 ACT visits/year (partial ≤41). |
| **van Vugt et al. (2011);** Netherlands | Small caseload (N.D.) | Yes (Includes vocational spec.) | Yes | N.D. | Unlimited | Mean DACTS total score: 3.3 (SD 0.4) at baseline; range 2.8–4.1. Two-year follow-up mean 3.3 (SD 0.3); range 2.7–4.0 | Key: Shared caseload and multidisciplinary team. Nice to have: Daily team meetings and vocational specialist inclusion. |
| **van Vugt et al. (2012);** Netherlands | N.D. | Yes (Consumer-providers) | N.D. | N.D. | N.D. | DACTS used (item 28); DACTS total score N.D. | Key: High-fidelity label. Nice to have: Use of consumer-providers (peers) in the team. |
| **van Vugt et al. (2014);** Netherlands | N.D. | N.D. | N.D. | N.D. | N.D. | DACTS total fidelity mean: 3.3 (SD 0.4) at baseline; 3.3 (SD 0.3) | Nice to Haves: Integrated substance abuse treatment |
| **Vidal et al.** (2020); Switzerland | N.D. | Yes (Mobile team) | N.D. | No | Unlimited | DACTS: 3.82 (moderate fidelity). | Key: Geneva’s ACT mobile team. Nice to have: unlimited care but designed as specific and transitory to facilitate transition to standard psychiatric care |
| **Wasmer et al.** (1999); USA | 10:1 | Yes | Yes (In vivo) | N.D. | Flexible (not time-limited) | N.D. | Key: Assertive intervention and community-based approach. Nice to have: Short-term intensive care with follow-up in community centers (no "no-discharge" policy) |
| **Waynor et al. (2020)**; USA | Low ratio | Yes | Yes | Yes | N.D. | N.D. | Nice to have: Mandatory inclusion of a vocational specialist. |
| **Wilk et al. (2013)**; Canada | Low ratio (N.D.) | Yes | Yes | Yes | Unlimited | Mean DACTS across teams: 4.12/5 (good fidelity); team range: 3.81–4.42. | Key: Strictly based on the original 1980 PACT model. |
| **Wolff et al. (1997)**; USA | 10–15:1 | Yes | Yes | Yes (24-hour emergency services) | N.D. | N.D. | Adaptation: No assigned psychiatrist/nurse (uses community services). |
| **Wood et al. (1995)**; New Zealand | N.D. | Yes (psychiatrist, social worker, and domiciliary nurse). | Yes (home visits; community outreach; phone contacts). | Yes (Provided by the existing psychiatric emergency service (outside routine working hours)) | Long-term | N.D. | Key: Weekly team meetings and coordination with general practitioners. |
| **Zavradashvili (2010)**; Georgia | 4.3:1 | Yes (two psychiatrists, two part-time psychologists, two nurses, and one social worker) | Yes (home visits, outpatient visits, telephone contacts, and community outreach/meetings) | N.D. | N.D. | N.D. | Adaptation: 3 phases with decreasing intensity. |
| **Zhao et al. (2015)**; China | N.D. | Yes (psychiatrists, psychiatric nurses, clinical psychologists, social workers, and a vocational specialist). | Yes (2–3 home/community visits per week). | N.D. | N.D. | DACTS: 3.7/5 (moderate fidelity)  Modified DACTS: 4.1/5 (good fidelity after excluding substance abuse items). | Nice to have: Culturally adapted family psychoeducation delivered every 2 weeks (12 sessions) |

**Note:** This table reports structural and organizational components commonly considered “critical ingredients” of ACT and used as proxies of fidelity, including caseload/ratio, multidisciplinary team configuration, in vivo/community-based service delivery, 24/7 crisis availability, and continuity of support. When formal fidelity instruments or scores were reported by the original studies (e.g., DACTS, TMACT, FACT fidelity scales, or other tools), these are specified; otherwise, they are indicated as Not reported. **Abbreviations:** ACT, Assertive Community Treatment; PACT, Program of Assertive Community Treatment; FACT, Flexible Assertive Community Treatment; OPUS, specialized early intervention model for first-episode psychosis (Denmark); MIRT, Mobile Intensive Rehabilitation Team; DACTS, Dartmouth Assertive Community Treatment Scale; TMACT, Tool for Measurement of Assertive Community Treatment; N.D., not described/not reported; NR, not reported; 24/7, 24 hours/7 days per week.
